# Sequence‐Encoded Frustration Directs the Formation of Abridged G‐Quadruplex Architectures

**DOI:** 10.1002/anie.8309343

**Published:** 2026-05-04

**Authors:** Yuncheng Qian, Mohamed Y. Ali, Andreas I. Karsisiotis, Paul Dillon, Scarlett A. Dvorkin, Peter A. C. McPherson, Mateus Webba da Silva

**Affiliations:** ^1^ Biomedical Sciences Research Institute Ulster University Coleraine UK

**Keywords:** abridged G‐quadruplex, competitive capping, density functional theory, folding frustration, G‐triplex, metadynamics, SRCD

## Abstract

Frustration—competing interactions that cannot be simultaneously optimized—shapes energy landscapes in proteins and soft matter, but has rarely been exploited as a programmable design principle in nucleic acids. Here we demonstrate that sequence‐encoded frustration programmes DNA G‐quadruplex folding, yielding stable “abridged” architectures with fewer guanine tetrads than the sequence would nominally permit. Using an integrated structural, spectroscopic, thermodynamic, and computational approach, we map a frustration‐biased folding landscape featuring a thermodynamically stabilized G‐triplex intermediate, whose identity we assign via TD‐DFT computed electronic circular dichroism spectra, and resolve the dominant unfolding pathway at atomic resolution. These results demonstrate programmable frustration as a predictive design principle for controlling nucleic acid topology and dynamics, offering new strategies for engineering functional DNA‐based systems and for interpreting genomic G‐quadruplex plasticity.

## Graphical abstract

Programmable frustration reshapes the free‐energy surface (FES) of guanine‐quadruplex (G4) folding, stabilizing an abridged G4 and a G‐triplex intermediate.

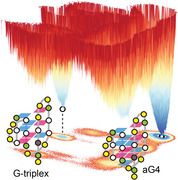

## 1 Introduction

Frustration—the inability to simultaneously satisfy competing interactions—is a fundamental organizer of energy landscapes across chemistry and biology. In proteins, frustration sculpts rugged energy landscapes, stabilizes non‐native intermediates, and can be exploited to engineer functional metastability [1, 2]. Extending this paradigm to nucleic acids, however, remains largely unexplored, despite the potential to programme folding pathways and control functional dynamics in DNA‐based systems.

DNA G‐quadruplexes (G4s)—stacks of Hoogsteen‐paired guanine tetrads—offer an ideal platform to test this concept because their topologies emerge from a balance of intrinsic, sequence‐encoded constraints and environmental factors [3, 4, 5, 6, 7, 8]. Yet predicting that balance remains difficult: guanine‐rich sequences often fail to adopt the “maximally stacked” tetrad stem that a naïve guanine count would suggest. Instead, stable architectures frequently form fewer tetrads than they could in principle support, implying that some guanines are diverted into competing terminal stabilization motifs through capping and loop organization. Despite many individual examples [4, 9, 10, 11, 12], this behavior has not been formulated as a general, quantitative consequence of an underlying energetic competition—and thus we still lack design rules that map sequence and conditions onto a specific G4 architecture. The same gap is reflected in the uneven reliability of current structure–prediction methods (including AlphaFold3) for noncanonical and context‐dependent G4 folds [13], underscoring the need for an interpretable, physics‐based framework for G‐quadruplex design and prediction.

Here, we propose that these “abridged” G‐quadruplexes arise from sequence‐encoded folding frustration: a quantifiable competition between extending the tetrad stem and optimizing terminal stabilization through capping and loop organization. While conformational heterogeneity is a recognized feature of nucleic acid landscapes, a fundamental demonstration has been lacking: the rational design of a G‐quadruplex where encoded frustration deterministically programmes a stable architecture with fewer tetrads (N‐1) than the sequence could nominally support (N). In this framework, abridgement is not a folding error but an engineerable outcome. We introduce programmable frustration—a concept that transforms frustration from a descriptive feature of folding landscapes into an active, tunable design parameter for nucleic acid engineering. We encode this frustration into a designed sequence to bias the competition, capture an abridged architecture structurally, and uncover a thermodynamically stabilized G‐triplex intermediate on the folding landscape. By integrating experiment with free‐energy analysis, we extract a predictive, quantitative framework for tuning frustration via sequence and environment, linking specific design variables to G4 topology and dynamics. Together, our findings establish frustration as a programmable principle of DNA architecture, providing a conceptual and predictive framework not only for interpreting G‐quadruplex plasticity in genomic contexts, but also for rationally engineering nucleic acid architectures with designed folding pathways and controlled metastability.

## 2 Results and Discussion

### 2.1 Programming an Abridged G‐Quadruplex via Encoded Frustration

**FIGURE 1:**
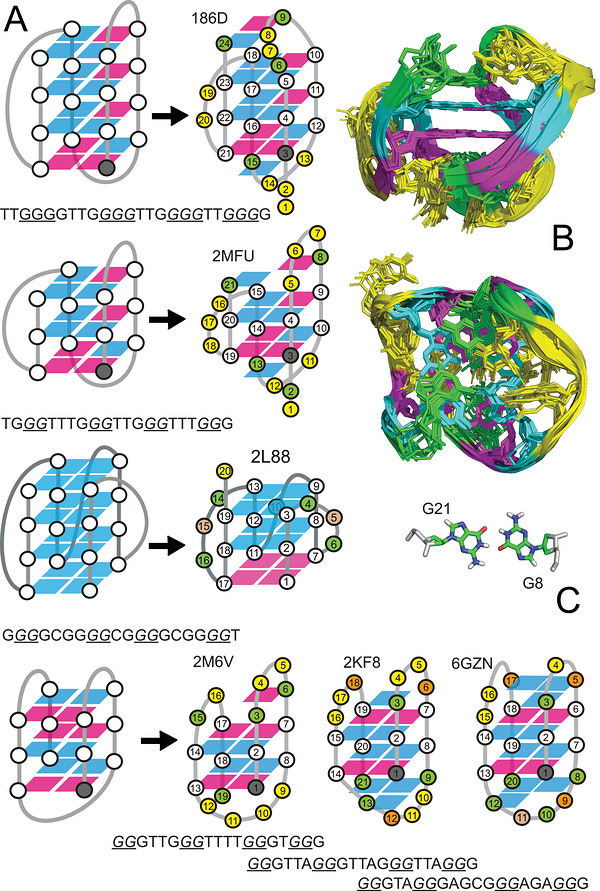
Frustrated folding in G‐quadruplex DNA architectures. (A) Schematic representations illustrate frustrated transitions between ideal and experimentally observed topologies using the *n*(L1, L2, L3) notation, where *n* is the number of stacked G‑tetrads and L1–L3 denote the three connecting loops in 5′→3′ order. Loop types are classified as lateral (*L*), propeller (*P*), or diagonal (*D*); for lateral loops, subscripts w/n/m indicate wide/narrow/medium grooves, and ± indicates loop direction around the stem. Sequences show guanines in syn (magenta) or anti (cyan) conformations, non‐stem guanines are shown in green, adenines in orange, and thymines in yellow. (B) Bundles of the 10 lowest‐energy NMR structures of 2MFU in sodium solution reveal a compact two‐stacked stem. (C) A G21:G8 mismatch stacks onto the top tetrad, and antiG13 stacks onto the bottom tetrad, both capping and stabilizing the truncated stem, exemplifying structural frustration as the loss of one G‐tetrad relative to its three‐stack potential.

Stable unimolecular G‐quadruplexes frequently adopt “abridged” architectures (aG4s), forming fewer guanine tetrads than their sequences could in principle support (Figure 1a) [4, 9, 10, 11, 12]. We hypothesized that these abridged folds arise from sequence‐encoded frustration—a competition between extending the tetrad stem and maximizing terminal stabilization through loops and capping motifs. To test whether frustration can be programmed as a design principle, we engineered a model sequence built around a programmable loop–length conflict.

G‐quadruplex folding is governed by a conditional interplay involving loop–length rules, *syn*/*anti* strand patterns, loop–stem interactions, loop–loop interactions, and cation regime: short (1–2 nt) loops tend to enforce propeller loops and parallel/hybrid folds, whereas 3‐nt segments are permissive and 4‐nt segments can act as selectors that realize specific lateral loops in defined groove directions [3, 4, 5, 6, 7, 8]. To encode frustration, we deliberately chose a nonselective loop–length combination (3‐2‐3) that lacks strong directional cues for a unique three‐tetrad fold. Our strategy was to force loop closure by recruiting a guanine into a terminal motif, thereby removing it from the tetrad stem and enforcing abridgement.

As a model for frustration‐driven abridgment, we targeted the 2(−Lw−Ln−P) topology, a two‐tetrad fold with a wide‐groove lateral loop, narrow‐groove lateral loop, and propeller loop in 5′→3′ order (Figure 1A). The ideal three‐tetrad analogue of this topology requires a four‐nucleotide first loop to form the −Lw connection, a one‐ or two‐nucleotide second loop for the −Ln, and a single‐nucleotide third loop for the propeller [4, 5, 8]. By instead choosing a nonselective 3–2–3 T‐only loop programme, we deliberately encoded a loop–length conflict. A functional −Lw loop can then form only via guanine sequestration, converting the first T_3_ segment into an effective TTTG loop and withdrawing that guanine from the stem. The 2‐nt second loop geometrically disfavors propeller and diagonal closures [4], enforcing a narrow‐groove lateral (−Ln), while a 5′ flanking thymine further suppresses diagonal‐loop formation. The third T_3_ loop remains permissive and can readily adopt the propeller conformation once the first two loops are set. The 3–2–3 programme is therefore intrinsically unable to support a three‐tetrad (−Lw−Ln−P) fold, forcing competition between stem completion and terminal capping. This design is particularly effective in Na^+^, where antiparallel families and induced‐fit pathways prevail [7, 14, 15], and a thymine‐only, nonselective loop programme is especially vulnerable to frustration. Under these conditions, relief of loop‐length frustration yields a two‐stacked aG4 by reducing the number of stem‐eligible guanines.

To materialize this design, we synthesized d(TG_3_T_3_G_3_T_2_G_3_T_3_G_3_) (2MFU) and two control sequences with the same 3–2–3 loop programme but different flanking contexts. While the controls yielded polymorphic mixtures in Na^+^, 2MFU formed a single, dominant species (Figures S1 and S2). The solution NMR structure of 2MFU confirmed the designed, compact 2(−Lw−Ln−P) architecture (Figure 1). Crucially, it revealed the predicted mechanism of frustration: guanine G8 was sequestered from the stem to form the first loop as a TTTG8 segment, creating the required selector −Lw motif and directly enforcing the two‐tetrad stem. Furthermore, the structure shows how terminal stabilization outcompetes stem completion: G21 forms a terminal G21:G8 mismatch that stacks neatly onto the top tetrad, while *anti‐*G13 provides complementary stacking at the opposite end (Figure 1C).

This outcome underscores a general principle: frustration and polymorphism are intrinsic to sequences with non‐selective, permissive loop lengths. The 3–2–3 programme—like symmetric 3–3–3 or 4–4–4 combinations—lacks the directional cues needed to define a unique three‐tetrad topology in Na^+^. To illustrate this, we examined the canonical sequence d(G_3_T_4_G_3_T_4_G_3_T_4_G_3_) with a uniform 4–4–4 T‐loop programme. As predicted, it formed a polymorphic mixture in Na^+^ (Figure S5), consistent with unresolved loop–length frustration.

Critically, this frustration can be actively relieved by imposing an external conformational constraint. Introducing a single riboguanosine (rG) into a strategic loop position of the polymorphic 4–4–4 sequence locks the adjacent guanine into an anti‐conformation, propagating a defined *syn*/*anti* pattern and enforcing a specific lateral–loop geometry. This collapses the polymorphic ensemble into a single, well‐defined 3(−LwD + Ln) topology (Figure S5). Thus, modest chemical editing can override frustration by providing the missing selective cue, demonstrating that frustration is not only encodable but also reversibly tunable.

The 2MFU structure, therefore, proves that frustration can be encoded prospectively to achieve a specific abridged architecture: a designed selector loop combined with a permissive T‐only 3–2–3 programme yields a native two‐tetrad fold in which terminal capping outcompetes formation of the third tetrad under Na^+^ conditions. This validates competitive capping as a predictive design lever and raises the central mechanistic question: Does this stability arise from a frustration‐biased folding pathway, equilibrium population reweighting, or both? We address this in the following sections.

### 2.2 Spectroscopic and Computational Identification of a G‐Triplex Intermediate

**FIGURE 2:**
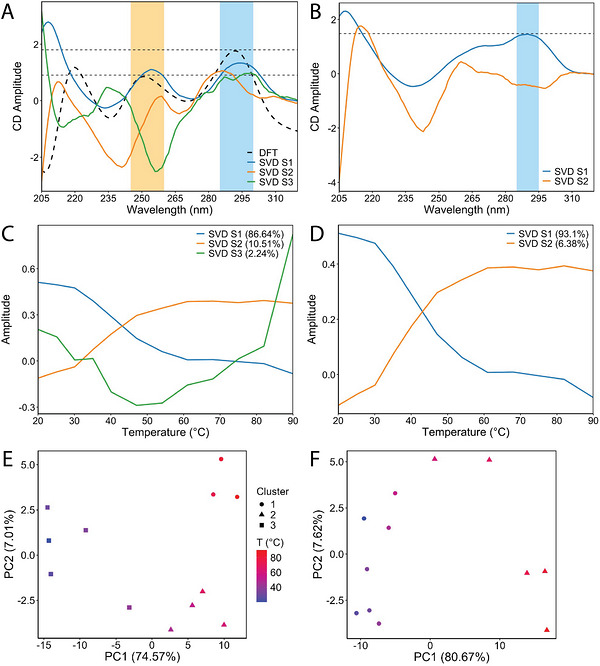
Spectroscopic trajectory of 2MFU G‐Quadruplex unfolding reveals a stable intermediate. (A,B) Truncated SVD of temperature‐dependent SRCD spectra identifies three low‐rank spectral signatures for 2MFU and two for 2JSL. These are interpreted as folded‐associated, intermediate‐associated, and unfolded‐associated signatures for 2MFU, and folded‐associated and unfolded‐associated signatures for 2JSL. Both architectures share the (−Lw−Ln−P) topology, but whilst 2MFU corresponds to an abridged two‐stacked stem, 2JSL retains the complete 3‐stack. The folded‐associated 2MFU spectral signature shows positive bands near 260 and 295 nm and a weakened trough near 235 nm, consistent with the TD‐DFT/ECD‐derived simulated spectrum (dashed line). (C,D) Nonnegative projection weights onto the retained low‐rank subspace show a broad intermediate‐associated contribution for 2MFU (∼47 C–82°C), whereas under the present conditions, 2JSL is adequately captured by a direct folded‐to‐unfolded transition at the resolution of the experiment (∼40 C–50°C). (E,F) PCA independently resolves three ensembles for 2MFU and two for 2JSL, with consistent color coding.

To determine whether the stability of the abridged G4 2MFU arises from a frustration‐biased folding route, we probed its thermal unfolding using synchrotron radiation circular dichroism (SRCD) and compared it to a canonical three‐tetrad control, 2JSL [16], which shares the same (−Lw−Ln−P) topology. The folded states of both sequences exhibit the characteristic peaks (≈260, ≈293 nm) and trough (≈240 nm) of their shared architecture [17]. Truncated SVD of the temperature‐dependent SRCD spectra revealed a fundamental difference: whereas 2JSL was adequately described by two significant low‐rank spectral contributions, 2MFU required three, consistent with a three‐state unfolding description (Figures 2). The intermediate‐associated contribution is prominent across a broad temperature window (∼47 C–82°C), and the associated spectral signature accounts for 10.5% of the total variance. This three‐state trajectory was independently confirmed by principal component analysis (PCA). The retained dimensionalities (rank 3 for 2MFU; rank 2 for 2JSL) are supported by scree analysis, lag‐1 autocorrelation, and rank‐truncated reconstruction/residual diagnostics provided in Figures S7.2–S7.7. Under the present K^+^‐buffered SRCD conditions and within the resolution of the current analysis, the control sequence 2JSL is adequately described by two significant low‐rank spectral contributions and an effective two‐state thermal trajectory. More broadly, the folding of human telomeric G‐quadruplexes is well known to be condition‐ and method‐dependent, with multistate behavior frequently observed in ensemble and single‐molecule studies [18, 19, 20]. We use 2JSL here as a same‐topology comparator, not as a universal mechanistic model for human telomeric G‐quadruplex folding.

The spectral signature of *S*_2_ indicates a partially structured state. It displays a strongly attenuated and blueshifted positive band (*λ*_max_ ≈ 285 nm) and an intensified negative band near 238 nm, which diverges sharply from the canonical folded‐state spectrum (Figure 2A). This pattern—diminished exciton coupling at long wavelengths and a redshifted, muted signal at mid‐range wavelengths—is characteristic of a structure that has lost the coherent *π*‐stacking of a complete G‐stem but retains more order than the fully unfolded chain. The exclusive presence of this stable intermediate in the abridged 2MFU, and its absence in the full‐stemmed control, demonstrates that the engineered architectural frustration actively reshapes the equilibrium energy landscape. Rather than a direct folded–unfolded transition, 2MFU populates a distinct intermediate basin, confirming that the encoded conflict between capping and stem elongation manifests not only in the final architecture but also in its folding pathway.

### 2.3 The Folding Intermediate is a G‐triplex

Because orthogonal SVD factors are not, by themselves, pure component spectra, structural assignment was based on the experimentally derived folded‐state and intermediate‐associated spectral signatures in conjunction with the global three‐state thermodynamic analysis (see Supporting Information), rather than on raw SVD vectors alone. To assign the structural identity of the stable intermediate *S*_2_, we computed electronic circular dichroism (ECD) spectra using time‐dependent density functional theory (TD‐DFT). We constructed two minimal models informed by experimental structures: (i) the native two‐tetrad G‐quadruplex stem of 2MFU, including its *syn*G8:*anti*G21 mismatch cap and anti‐G13 terminal stack, and (ii) a six‐guanine G‐triplex based on the validated NMR structure PDB 2MKM [21].

**FIGURE 3:**
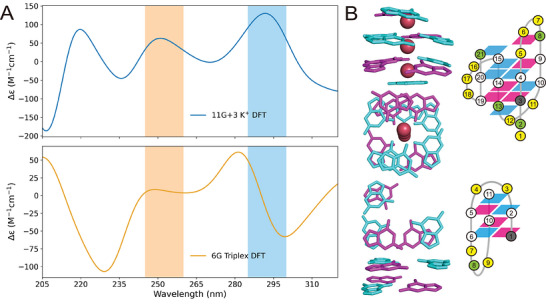
DFT‐optimized G‐quadruplex and G‐triplex models reproduce the experimental SRCD signatures of 2MFU. A) TD‐DFT/ECD spectra were simulated for the ground‐state G‐quadruplex stem of 2MFU with its (synG8:antiG21) mismatch on top, and antiG13 capping at the bottom, as well as for a six‐guanine triplex derived from the G‐triplex solution structure corresponding to PDB 2MKM. In the corresponding schematics, guanosines in syn and anti‐conformation are shown in magenta and cyan, respectively. (B) Optimized geometries (top and side views) illustrate the π‐stacking and triad arrangements underlying the computed spectra. The 2MFU model reproduces the folded‐state experimental signature, whereas the triplex model yields a deep ∼230 nm trough, a ∼245 nm shoulder/hump, and a ∼280 nm positive band consistent with the experimentally derived intermediate‐associated signature assigned to *S*_2_.

The calculated spectra achieve close qualitative agreement in band sign, placement, and overall lineshape after affine wavelength alignment (Figure 3). The G‐quadruplex model for 2MFU captures the experimentally derived folded‐state signature, including its characteristic positive bands near 293 and 250 nm and a weakened trough near 235 nm. Crucially, the triplex model closely mirrors the experimentally derived intermediate‐associated signature: a blueshifted positive band (∼280–283 nm), a deep trough at ∼230–238 nm, and only a shallow feature across 242–253 nm. This close agreement supports the assignment of the intermediate to a triplex‐like ensemble, and is consistent with the first inferred G‐triplex characterization by CD [22].

The spectral differences arise from fundamental architectural contrasts. The G‐quadruplex model features extended, coaxial *π*‐stacking across two tetrads, generating strong exciton coupling. In contrast, the G‐triplex comprises only two stacked G‐triads, resulting in shorter and interrupted *π*‐stacking that explains the attenuated long‐wavelength band and deepened trough of *S*_2_. This assignment integrates 2MFU into the broader mechanistic framework of G‐quadruplex landscapes [14, 23, 24, 25, 26, 27, 28, 29], where G‐triplex‐like states are recognized as likely intermediates [30, 31], and provides spectroscopic support, via TD‐DFT computed electronic circular dichroism spectra, of a thermodynamically populated G‐triplex intermediate on a G‐quadruplex unfolding landscape.

### 2.4 van't Hoff Analysis Reveals a Thermodynamically Stabilized G‐Triplex Basin

The thermal unfolding of the abridged G4 2MFU is quantitatively distinct from a conventional G‐quadruplex, requiring a three‐state model that features a stable, populated intermediate. Global fitting of temperature‐dependent SRCD spectra confirmed a sequential folding scheme (F ⇌ I ⇌ U) for 2MFU, while the control sequence 2JSL was adequately described by an effective two‐state fit to the observed thermal profile under the present SRCD conditions. This analysis quantifies the intermediate (I) as a significantly populated thermodynamic basin. At 25°C, the fits yield Δ*G*_(F vs. U)_ ≈ −7.49 kJ mol^−^^1^ and Δ*G*_(I vs. U)_ ≈ −3.11 kJ mol^−^^1^, indicating that the intermediate—whose experimental spectral signature is consistent with a G‐triplex‐like ensemble—remains appreciably populated under native conditions. Under these conditions, no additional thermodynamically distinct intermediate was resolved for 2JSL at the resolution of the present experiment.

**FIGURE 4:**
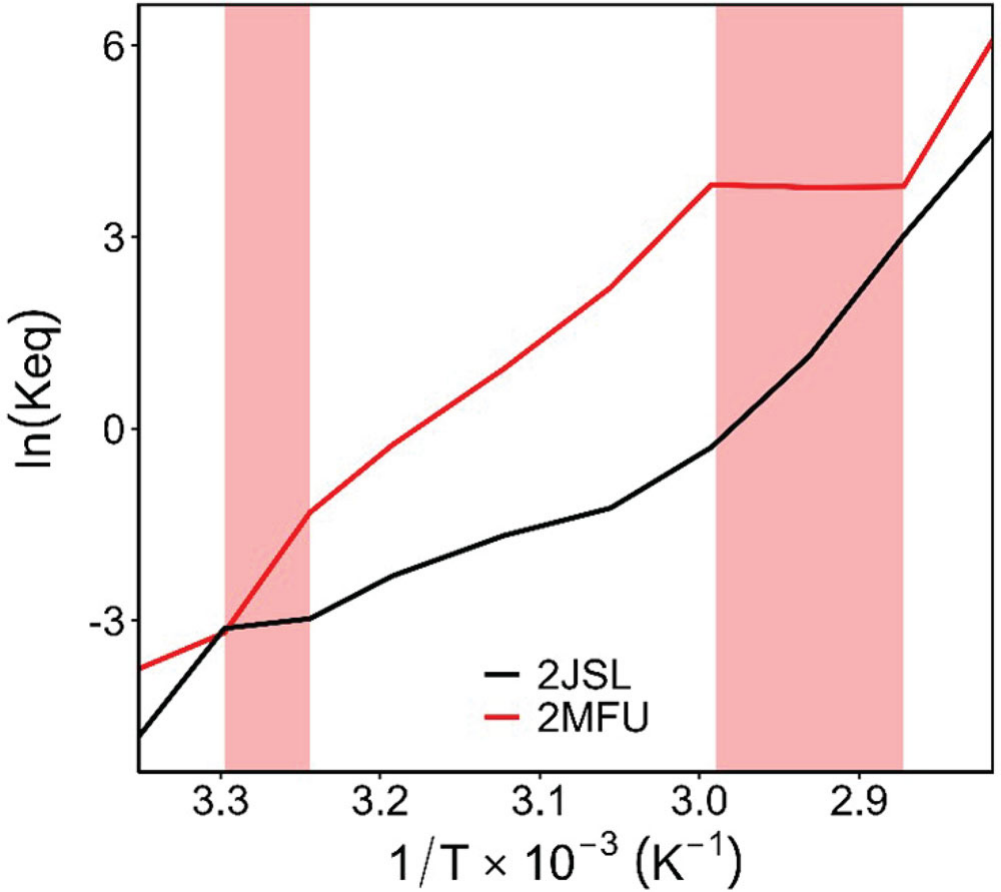
van't Hoff nonlinearity exposes an intermediate stabilization plateau in 2MFU. van't Hoff plots of the unfolding equilibrium (ln Keq) versus 1/T are shown for 2MFU (red) and 2JSL (black), obtained from SRCD‐monitored equilibria. The deviation from linearity and reduced slope in 2MFU within the 61 C–72°C region (shaded) mark a stabilization plateau corresponding to the *S*_2_ intermediate. In contrast, 2JSL shows no resolved stabilization plateau under the present experimental resolution.

The signature of this intermediate is a distinct stabilization plateau in the van't Hoff plot. For 2MFU, a pronounced curvature appears between 61 C and 72°C (Figure 4), where the apparent enthalpy change flattens, marking the temperature window in which the F ⇌ I equilibrium is most thermodynamically favorable. No such plateau is observed for 2JSL, whose van't Hoff trace remains near‐linear. This nonlinearity is robust across different spectral fitting protocols, confirming it reports a genuine intermediate stabilization.

The global thermodynamic analysis thus establishes a non‐two‐state landscape for the frustrated fold 2MFU, with a quantifiably stabilized G‐triplex basin. This result directly links the encoded architectural frustration to a reshaped energy surface, where competition between capping and stem elongation thermodynamically populates an intermediate that is absent in the canonical structure.

### 2.5 Experimentally Anchored Free‐Energy Landscape

**FIGURE 5:**
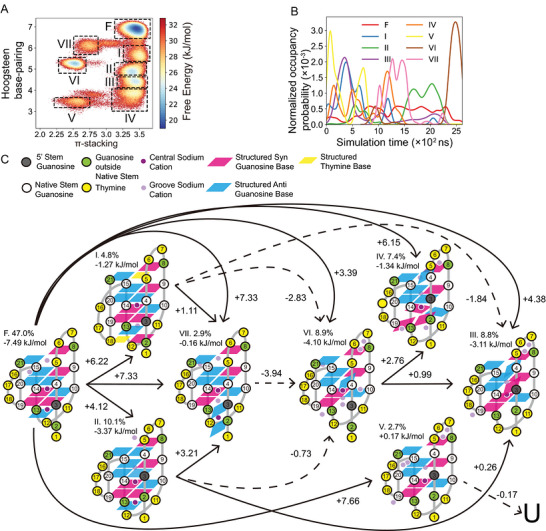
Bias‐exchange metadynamics maps the 5′‐strand‐directed route to the G‐triplex ensemble in 2MFU. (A) Two‐dimensional free‐energy surface (FES) from the neutral replica, projected onto minimal collective variables for *π*‐stacking (*x*) and Hoogsteen pairing (*y*); basins are labeled F (native) and I–VII. (B) Normalized basin occupancies over time show early access to I–II while F remains populated, followed by growth of triplex‐like states III and VI with occasional visits to IV and VII, consistent with progressive partial unfolding. (C) Mechanistic scheme with thermodynamic annotation at 25°C (populations and Δ*G* relative to U; CD‐anchored rescaling with F = −7.49 kJ·mol^−^^1^ and triplex ensemble = −3.11 kJ·mol^−^^1^). Colors/notation: syn guanine magenta, anti‐guanine cyan, thymine yellow, guanosine outside the stem green, central and groove Na^+^ dark and light purple; numbered circles indicate residue order. Solid arrows mark dominant transitions and dashed arrows rarer routes, supporting a 5′‐disengagement pathway F → I/II → III/VI on the way to U.

Having identified a G‐triplex intermediate by spectroscopy and thermodynamics, we next sought an atomistically detailed, experimentally anchored description of how this species emerges during partial unfolding. We therefore performed well‐tempered bias‐exchange metadynamics (WT‐BE‐MetaD) starting from the native NMR structure of 2MFU, using minimal collective variables (CVs) that quantify (i) *π*‐stacking and (ii) Hoogsteen base‐pairing within the G‐stem (definitions and biasing protocol in the Supporting Information). To test directional sensitivity, we performed two WT‐BE‐MetaD datasets in which the same unfolding CVs were applied to either the 3′‐end strand or the 5′‐end strand of the G‐stem (3′‐anchored vs. 5′‐anchored), using otherwise identical protocols (Supporting Information). The resulting two‐dimensional free‐energy surface reconstructed from the neutral replica reveals a rugged, multi‐basin landscape comprising the native fold (F) and seven metastable states (I–VII) (Figure 5A). Time‐resolved basin occupancies show an ordered progression: early excursions from F populate partially disrupted G4‐like states I and II, followed by growth of G‐triplex basins III and VI (with a minor triplex microstate V) and intermittent visits to IV and VII before further loss of native contacts (Figure 5B; see also Supporting Information).

To connect the simulation landscape directly to experiment, we applied a CD‐anchored rescaling of the metadynamics free‐energy surface using the experimentally derived stabilities at 25 °C, fixing the native state at Δ*G*_(F vs. U)_ = −7.49 kJ mol^−^^1^ and the G‐triplex intermediate ensemble at Δ*G*_(I vs. U)_ = −3.11 kJ mol^−^^1^. Because these anchoring free energies were derived from the sodium‐buffered SRCD thermodynamic analysis of 2MFU, the experimentally connected landscape in Figure 5 should be interpreted as the sodium‐conditioned unfolding route for this designed architecture, rather than as a cation‐independent G4 unfolding map. Anchoring requires an order‐preserving (monotonic) rescaling of basin free energies; under identical two‐point anchoring, only the 5′‐anchored surface admits a monotonic mapping, whereas the 3′‐anchored surface would invert non‐native stability ordering (see Supporting Information for the 3′ comparison). After this anchoring, the pooled triplex ensemble (III/V/VI) aligns quantitatively with the spectroscopically observed *S*_2_ intermediate (Figure 5C), identifying the 5′‐disengaged G‐triplex ensemble as the structural counterpart of the thermodynamic intermediate. The resulting mechanistic scheme (Figure 5C) supports a 5′‐strand disengagement pathway: F → I/II → III/V/VI → U, where I/II represent partially disrupted (G4‐like) states on the way to the triplex ensemble. In this pathway, the −Lw(TTTG8) loop geometry positions G8 to stack over the terminal tetrad, structurally predisposing the 5′ end for slippage while maintaining strong terminal capping and cation coordination that stabilize G‐triplex conformers. Together, TD‐DFT/ECD‐assigned spectroscopy, van't Hoff thermodynamics, and Δ*G*‐anchored metadynamics provide a quantitatively connected route from sequence design to intermediate stabilization and pathway selection.

### 2.6 A Design Framework for Programmable Frustration

**FIGURE 6:**
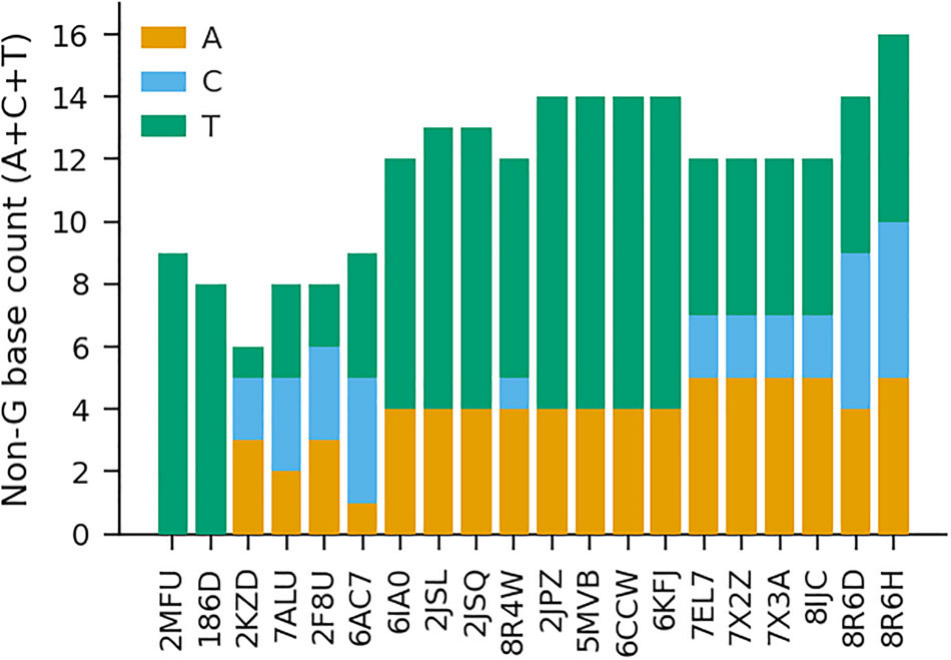
Loop nucleotide composition signatures of (−Lw–Ln–P) G‐quadruplex folds. For each PDB entry, stacked bars report the integer counts of adenine (A), cytosine (C), and thymine (T) within the loop segments; guanines (G) forming the stem are not shown. PDB codes are on the *x*‐axis. With the exception of 2MFU and 186D (abridged architectures), the constructs adopt the maximum number of tetrad stacking permitted by their guanine‐segment composition.

To test whether the stem‐capping competition observed in 2MFU generalizes, we surveyed reported structures adopting the same (−Lw–Ln–P) topology (Figure 6). A clear compositional trend emerges: non‐abridged constructs contain adenines in their loops and achieve maximal tetrad stacking, whereas the abridged exemplars 2MFU and 186D are adenosine‐free and deviate from this limit by recruiting stem‐eligible guanines into terminal capping motifs. This partition supports a simple allocation principle: when adenines are available, they provide efficient aromatic capping that preserves stem completion; in their absence, terminal stabilization competes directly with stem extension, favouring abridged architectures.

This logic can be formalized into a unifying framework in which stem‐capping competition is modulated by four tunable design levers:

1. Loop composition and length. The chemical identity of loop residues determines the availability of non‐stem capping bases (e.g., adenine). Loop–length combinations define the accessible connectivity “menu,” where nonselective programmes (e.g., near 3‐3‐3 or 4‐4‐4) can increase frustration and architectural degeneracy.
2. Solution conditions. Cation identity (Na^+^ vs. K^+^) and concentration are expected to reweight the stability of loop‐cap microstates and intermediate ensembles, thereby modulating the expression of frustration under different environmental conditions. In the present study, the principal 2MFU experiments were performed in Na^+^ buffer, whereas the 2JSL comparator was analyzed under standard K^+^ conditions (Supporting Information).
3. Auxiliary sequence context. Residues 5’ to first G‐tract, or 3’ to fourth G‐tract (pre‐ and post‐flanking residues or segments), can act as a reservoir of capping interactions, alleviating the need to recruit stem guanines and thereby rescuing full stacking even in permissive loop contexts.
4. G‐tract capacity. The number and continuity of guanines set the upper bound of possible tetrads, defining the resource pool for the allocation trade‐off.

Within this framework, abridgement is a designable outcome that becomes favorable when terminal stabilization is achieved by recruiting stem‐eligible guanines. Critically, the explanatory power of this framework is supported not only by the structural survey but also by controlled intervention: altering a single design lever can relieve frustration and restore full stem completion, as demonstrated by the rG editing experiment, which rescues a unique, fully stacked fold from a frustrated, polymorphic ensemble. Thus, the framework rationalizes observed architectural choices and provides a principled basis for converting sequence and solution conditions into testable hypotheses for whether a given G‐quadruplex will abridge or fully stack. Importantly, the framework is prospective in the sense that it yields falsifiable design hypotheses: for example, it predicts that a sequence combining a nonselective 3‐3‐3 loop programme with an adenosine‐free loop composition should be particularly prone to stem‐capping competition and therefore biased toward abridgement (or enhanced intermediate stabilization), a prediction that can be tested directly by designing such a construct and determining its topology by CD/NMR under matched ionic conditions. We do not propose that a triplex‐like intermediate is a universal hallmark of all abridged G‐quadruplexes; rather, in 2MFU it is the experimentally resolved consequence of a specific stem‐capping conflict, and whether comparable intermediates appear in other aG4s is expected to depend on topology, loop programme, and solution conditions.

## 3 Conclusion

Here, we show that frustration can be deliberately encoded as a programmable design variable for nucleic‐acid folding landscapes, extending a framework widely used in proteins and soft matter to DNA architecture. By encoding a stem‐capping conflict, we programme a G‐quadruplex to adopt a stable abridged topology with one fewer guanine tetrad than would nominally be expected from its G‐tract capacity. This designed frustration reshapes the folding landscape, generating a meaningful metastable state—a thermodynamically populated G‐triplex intermediate—beyond simple two‐state behaviour. Together, these findings provide a physics‐based, predictive logic linking tunable sequence and environmental inputs to G‐quadruplex topology and dynamics, complementing data‐driven structure prediction approaches. These findings demonstrate programmable frustration as a predictive design principle in DNA G‐quadruplex architecture, explaining how abridged architectures can emerge and how frustration can be rationally encoded or relieved. This offers a route to interpret genomic G‐quadruplex plasticity and to engineer DNA‐based systems with prescribed folding pathways and condition‐responsive function.

## Author Contributions

**Yuncheng Qian**: investigation, formal analysis, validation, visualization, writing ‐ original draft, writing ‐ review & editing. **Mohamed Y.Ali**: investigation, validation, formal analysis, visualization, methodology, writing – original draft, writing – review & editing. **Andreas I. Karsisiotis**: investigation, formal analysis, writing ‐ review & editing, validation, data curation. **Paul Dillon**: writing ‐ review & editing, investigation, formal analysis, visualization, validation. **Scarlett A. Dvorkin**: writing – review & editing, investigation, formal analysis. **Peter A. C. McPherson**: writing – review & editing, methodology, resources. **Mateus Webba da Silva**: supervision, project administration, visualization, investigation, conceptualization, methodology, funding acquisition, writing ‐ original draft, writing ‐ review & editing, formal analysis, validation, resources.

## Conflicts of Interest

The authors declare no conflicts of interest

## Supporting information




**Supporting File**: anie72460‐sup‐0001‐SuppMat.docx.

## Data Availability

The data that support the findings of this study are openly available in Protein Data Bank as PDBid 2MFU (https://doi.org/10.2210/pdb2MFU/pdb), Biological Magnetic Resonance Bank as BMRB 19572 (DOI:10.13018/BMR19572), and Zenodo (https://doi.org/10.5281/zenodo.15832342)
