# Supplementary material for: Sequence‐Encoded Frustration Directs the Formation of Abridged G‐Quadruplex Architectures
Source: Angew Chem Int Ed Engl. 2026 May 4;65(25):e8309343. doi: 10.1002/anie.8309343 (PMC13266908; doi:10.1002/anie.8309343)
Supplement: Supplementary file 1 — Supporting File: anie72460‐sup‐0001‐SuppMat.docx. [file ANIE-65-e8309343-s001.docx]

**SUPPLEMENTARY MATERIAL**

**Sequence encoded frustration directs the formation of abridged G-quadruplex architectures**

Yuncheng Qian,**^†^** Mohamed Y. M. M. Ali,**^†^** Andreas I. Karsisiotis, Paul Dillon, Scarlett A. Dvorkin, Peter A. C. McPherson, Mateus Webba da Silva*

Biomedical Sciences Research Institute, Coleraine, Cromore Road, BT52 1SA, UK.

(*) mm.webba-da-silva@ulster.ac.uk

Table of Content

[METHODS 3](#_Toc225340514)

[I. Sample preparation 5](#_Toc225340515)

[**Figure S1.** Folding in non-discriminating combinations of loop lengths in DNA putative 3- and 4-stacked G-quadruplex folding sequences by 1D NMR shown in 80 mM NaCl, 20 mM NaH_2_PO_4_/Na_2_HPO_4_, and pH 6.8. 6](#_Toc225340516)

[II. NMR spectral assignments 6](#_Toc225340517)

[**Figure S2.** Solution structure characterization and structural details of DNA sequence d(TG_3_T_3_G_3_T_2_G_3_T_3_G_3_) (2MFU) in 80 mM NaCl, 20 mM NaH_2_PO_4_/Na_2_HPO_4_, and pH 6.8. 7](#_Toc225340518)

[**Figure S3.** A [^31^P-^1^H] HSQC correlation spectrum showing ^31^P assignments for the d(TG_3_T_3_G_3_T_2_G_3_T_3_G_3_) DNA sequence (2MFU) 8](#_Toc225340519)

[**Figure S4.** Exchangeable proton NMR assignments 9](#_Toc225340520)

[**Figure S5.** Combinations of four-residue long segments can be non-selective in canonical quadruplexes. 11](#_Toc225340521)

[III. Structure calculations 12](#_Toc225340522)

[**Table S1**. NMR restraints and structural statistics for 10 selected structures for the unimolecular d(TG_3_T_3_G_3_T_2_G_3_T_3_G_3_) quadruplex. 12](#_Toc225340523)

[IV. SRCD spectroscopy 13](#_Toc225340524)

[**Figure S6**. Evaluation of equilibrium thermodynamics of 2MFU from global multi-wavelength CD (260–300 nm): state populations and temperature-derivatives of ΔG. 16](#_Toc225340525)

[**Figure S7.1.** Unfolding intermediates of 2MFU resolved by t-SNE clustering of dichroic spectra. 17](#_Toc225340526)

[**Figure S7.2**. Scree plot and cumulative explained variance for 2MFU. 19](#_Toc225340527)

[**Figure S7.3**. Scree plot and cumulative explained variance for 2JSL. 19](#_Toc225340528)

[**Figure S7.4**. Lag-1 autocorrelation coefficients for SVD vectors of 2MFU. 20](#_Toc225340529)

[**Figure S7.5**. Lag-1 autocorrelation coefficients for SVD vectors of 2JSL. 20](#_Toc225340530)

[**Figure S7.6**. Rank-3 reconstruction and residual analysis for 2MFU. 21](#_Toc225340531)

[**Figure S7.7**. Rank-2 reconstruction and residual analysis for 2JSL. 22](#_Toc225340532)

[V. Standard MD simulations 22](#_Toc225340533)

[**Table S2.** Summary of simulation systems utilized in standard MD, well-tempered bias-exchange metadynamics (WT-BEMetaD), and pre-DFT empirical MD refinements, DFT geometry refinements. 24](#_Toc225340534)

[**Figure S8.2**. Glycosidic bond angles (GBA) and π-stacking interactions of three consecutive guanines in four G-strand during the 300 ns standard MD simulation of 2MFU 26](#_Toc225340535)

[VI. WT-Bias-Exchange Metadynamics (WT-BEMetaD) 26](#_Toc225340536)

[**Figure S9.** Schematic construction of collective variables (CVs) for π-stacking and Hoogsteen base pairing in the native G-stem topology 28](#_Toc225340537)

[**Table S3.** Definition of residue and atom pairs used for constructing global and minimal collective variables (CVs) in π-stacking and Hoogsteen base pairing. 30](#_Toc225340538)

[**Figure S10**. Rational Switching Functions Defining CVs for WT-Bias-Exchange MetaD of 2MFU (π-Stacking and Hoogsteen Contacts) 31](#_Toc225340539)

[**Table S4.** WT-BEMetaD simulations of all stepwise attempts, differing in bias factors of the global collective variables, and the scopes of the minimal collective variables. 32](#_Toc225340540)

[**Figure S11.** Bias-factor optimization in WT-Bias-Exchange MetaD for unfolding of the native G-stem of 2MFU 32](#_Toc225340541)

[VII. Free-energy reconstruction and convergence 33](#_Toc225340542)

[**Table S5**. Re-scaled energies 5′-end–biased FES (BF 45) (I = pooled {5′‑III, 5′‑V, 5′‑VI}). 34](#_Toc225340543)

[**Table S6**. Re-scaled energies 3′-end–biased FES (BF 45) (I = pooled {3′‑II, 3′‑III}) 35](#_Toc225340544)

[**Figure S12**. 3′-end-directed unfolding of 2MFU at optimal bias factor (BF = 45) in WT-Bias-Exchange MetaD. 36](#_Toc225340545)

[**Figure S13**. The 1D projections and convergence of WT-BEMetaD strategies on the neutral replicas of minimal CV for exploring 5′-end and 3′-end bias mechanisms (bias factor 45) 37](#_Toc225340546)

[**Figure S14**. The free energy statistics of WT-BEMetaD strategies in each basin of minimal CV for exploring 5′-end and 3′-end bias mechanisms (Bias factor 45) 38](#_Toc225340547)

[**Figure S15**. Structures of step-wised unfolding mechanism of 5′-end bias 39](#_Toc225340548)

[VIII. TD-DFT/ECD calculations 40](#_Toc225340549)

[**1. 2MFU model preparation and TD‑DFT calculations** 40](#_Toc225340550)

[**Implementation notes** 41](#_Toc225340551)

[**Table S7**. Pre-DFT optimization on 2MFU G-quadruplex DNA with empirical distance restraints in Amber MD simulation 42](#_Toc225340552)

[**Table S8**. Constrained hydrogen-bond distances used in DFT geometry optimization of 2MFU G-quadruplex DNA structures 43](#_Toc225340553)

[**Figure S16**. K⁺ stabilizes G-quadruplex π-stacking and central ion channels more effectively than Na⁺ in DFT-optimized 2MFU ground states 44](#_Toc225340554)

[**Figure S17**. Optimization of CD spectral broadening highlights Gaussian dynamic broadening as optimal for 2MFU 11G K⁺ system 45](#_Toc225340555)

[**Figure S18**. Optimization of CD spectral broadening highlights Gaussian dynamic broadening as optimal for 2MFU 8G K⁺ system 46](#_Toc225340556)

[**Table S9**. Wavelengths of selected excited states (in nm) computed from TD-DFT for 2 DFT 2MFU systems with potassium 47](#_Toc225340557)

[**2. Triplex model preparation and TD‑DFT calculations** 47](#_Toc225340558)

[**Table S10.** Pre-DFT optimization on G-triplex with empirical distance restraints in Amber MD simulation derived from the architecture of 2MKM 48](#_Toc225340559)

[**Table S11.** Constrained hydrogen-bond distances used in DFT geometry optimization of triplex and hybrid triplex structures. 49](#_Toc225340560)

[**Figure S19.** Raw simulated circular dichroism (CD) and absorption spectrum from TD-DFT excited states for G-triplex 50](#_Toc225340561)

[**Table S12.** Wavelengths of selected excited states (in nm) computed from TD-DFT for G-triplex structure 50](#_Toc225340562)

[**Figure S20**. Final simulated circular dichroism (CD) spectra of representative intermediate structures using optimized dynamic broadening widths for G-triplex 51](#_Toc225340563)

[**Figure S21.** Broadening, redshifted and rescaled circular dichroism (CD) spectra of representative intermediate structures before conversion of units of G-triplex. 51](#_Toc225340564)

[IX. Integrative summary 52](#_Toc225340565)

[**Table S13**. Summary of stacking poses across Guanosine step among NMR-derived, metadynamic basin, and the DFT ground state 53](#_Toc225340566)

[References 54](#_Toc225340567)

# METHODS

**Oligonucleotides 2MFU and 2JSL sample preparation.** DNA oligonucleotides, including 2MFU (d(TG₃T₃G₃T₂G₃T₃G₃)) and 2JSL (d(TAG₃T₂AG₃T₂AG₃T₂AG₃T₂)), were synthesized and reverse-phase HPLC-purified. Samples were exchanged into the specified cation buffers and quantified by UV spectrophotometry. Standard conditions were: Na⁺ buffer (20 mM sodium phosphate, 80 mM NaCl, pH 6.8) for 2MFU, and K⁺ buffer (90 mM total K⁺, pH 7.0) for 2JSL. A secondary sodium condition (40 mM Na⁺; 20 mM NaPi + 20 mM NaCl, pH 6.8) was used for selected comparative NMR analyses.

**NMR spectroscopy and structure determination.** NMR samples contained ~4 mM DNA and spectra were acquired on a Varian 500 MHz spectrometer (5–20 °C). Complete resonance assignments were obtained from standard 2D experiments (NOESY, DQF-COSY, TOCSY, ¹H–³¹P HSQC, and long-range correlations). Structure calculations were performed in XPLOR-NIH using 608 experimental restraints; the 10 lowest-energy conformers showed no NOE violations >0.2 Å and a pairwise heavy-atom RMSD of 1.10 ± 0.18 Å (PDB 2MFU; BMRB 19572).

**Synchrotron radiation circular dichroism.** SRCD spectra were recorded on beamline B23 (Diamond Light Source) from 185–330 nm (0.02 cm path length) over 4.5–90 °C; reversibility was verified by pre-/post-melt overlays. Temperature-dependent spectra were globally fit using a van’t Hoff formalism to a sequential three-state model (F ⇌ I ⇌ U) for 2MFU and a two-state model (F ⇌ U) for 2JSL. Uncertainties were estimated by bootstrap resampling, and dimensionality-reduction / spectral decomposition methods were used as independent validation of state dimensionality.

**Molecular dynamics and enhanced sampling.** All-atom MD simulations were initiated from the lowest-energy NMR structure of 2MFU using the AMBER OL21 force field and TIP3P water in a truncated octahedral box (≥25 Å), with ~100 mM NaCl (Joung–Cheatham parameters). Following minimization and equilibration, three independent 300 ns NPT runs were performed at 300 K and analyzed for global stability and local structural descriptors. To sample unfolding pathways, well-tempered bias-exchange metadynamics (WT-BE-MetaD) employed three replicas (neutral, π-stack-biased, Hoogsteen-biased) exchanging every 30 ps. Two collective variables captured cooperative π-stacking integrity and Hoogsteen base-pairing within the native stem; bias-factor scans identified BF = 45 as providing robust intermediate sampling while retaining the native basin. Aggregate enhanced-sampling time exceeded 2 µs.

**Free-energy reconstruction and experimental anchoring.** Two-dimensional free-energy surfaces were reconstructed from neutral replicas at 300 K by reweighting. Metastable basins were identified by clustering in CV space. To enable quantitative comparison with experiment, FES energies were affinely rescaled so that the free-energy levels of the native basin (F) and a pooled triplex-intermediate basin (I) matched ΔG values obtained from SRCD at 25 °C, with U as the zero reference.

**TD-DFT calculations of CD spectra.** Minimal folded-state models were built from truncated 2MFU core structures (8 or 11 guanosines with three channel cations) extracted from the NMR-refined architecture. For the intermediate assignment, a separate six-guanine G-triplex comparator was constructed from the solution NMR structure PDB 2MKM. TD-DFT/ECD spectra were computed in ORCA using CAM-B3LYP/def2-TZVP with CPCM (water) and converted to continuous spectra by dynamic Gaussian broadening with affine wavelength alignment to the experimental SRCD window.

**Data deposition**. Structure determination details have been deposited in the Biological Magnetic Resonance Bank (BMRB) with identification number 19572- doi:10.13018/BMR19572. The calculated structures we deposited in the Protein Data Bank (PDB) with identification 2MFU- doi.org/10.2210/pdb2MFU/pdb. Computaional aspects of the work have been deposited in Zenodo (DOI: 10.5281/zenodo.15832342).

# I. Sample preparation

DNA oligonucleotides (2MFU: d(TG₃T₃G₃T₂G₃T₃G₃); 2JSL: d(TAG₃T₂AG₃T₂AG₃T₂AG₃T₂); d(G_3_T_3_G_3_T_2_G_3_T_3_G_3_); d(TG_3_T_3_G_3_T_2_G_3_T_3_G_3_A); d(G_4_T_3_G_4_T_2_G_4_T_3_G_4_); d(TG_4_T_3_G_4_T_2_G_4_T_3_G_4_); d(G_3_T_3_G_3_T_3_G_3_T_3_G_3_); d(G_3_T_4_rGG_2_T_4_G_3_T_4_rGG_2_ by reverse-phase HPLC (Phenomenex Clarity Oligo-RP, 10 × 100 mm, 5 μm, C18) using a 100 mM TEAA buffer (pH 6.8) and an acetonitrile gradient (5–40% over 35 min). Fractions were desalted on Sephadex G-15, dialyzed, and lyophilized. Final 2JSL was resuspended in 70 mM of KCl and 20 mM of KPi (pH 7.0). The other sequences were resuspended in 80 mM NaCl and 20 mM NaPi (pH 6.8).

| *GGG*TTT*GGG*TT*GGG*TTT*GGG* |  |
| --- | --- |
| T*GGG*TTT*GGG*TT*GGG*TTT*GGG*A |  |
|  |  |
| *GGGG*TTT*GGGG*TT*GGGG*TTT*GGGG* | ­ |
| T*GGGG*TTT*GGGG*TT*GGGG*TTT*GGGG* |  |
| *GGG*TTT*GGG*TTT*GGG*TTT*GGG* |  |
| *GGGG*TTT*GGGG*TTT*GGGG*TTT*GGGG* |  |

**Figure S1.** Folding in non-discriminating combinations of loop lengths in DNA putative 3- and 4-stacked G-quadruplex folding sequences by 1D NMR shown in 80 mM NaCl, 20 mM NaH_2_PO_4_/Na_2_HPO_4_, and pH 6.8. The imino region of the NMR spectrum shows that none of these sequences demonstrates single architectures formed.

# II. NMR spectral assignments

**Solution NMR experiments were utilized to derive the structure of 2MFU**

NMR samples contained 4 mM DNA in 20 mM sodium phosphate (pH 6.8) with 80 mM NaCl. Spectra were acquired on a Varian 500 MHz spectrometer at 5-20 °C. Experiments included NOESY (70-240 ms mixing, 100% D₂O), JR-NOESY (60/200 ms, 90% H₂O/10% D₂O), DQF-COSY, TOCSY, and [¹H-³¹P] HSQC (100% D₂O), plus JR-HMBC for imino-aromatic correlations (90% H₂O/10% D₂O at 10 °C). Data were processed in FELIXNMR. Representative spectra and connectivities are shown in **Figs. S2-S4**. Structural statistics appear in **Table S1**.


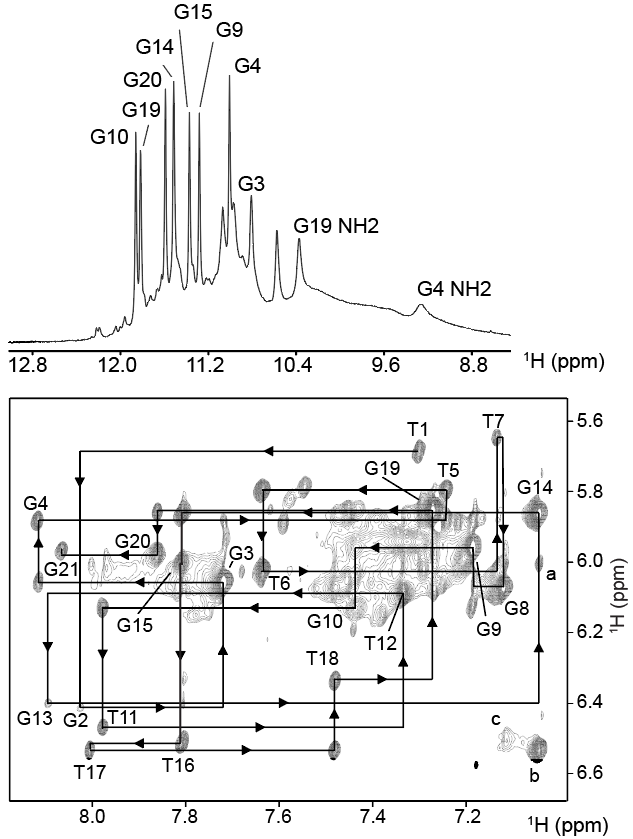


**Figure S2.** Solution structure characterization and structural details of DNA sequence d(TG_3_T_3_G_3_T_2_G_3_T_3_G_3_) (2MFU) in 80 mM NaCl, 20 mM NaH_2_PO_4_/Na_2_HPO_4_, and pH 6.8. In panel (A) expansion of 1D NMR spectrum of imino proton region at 5 ^o^C, and its circular dichroism signal at 20 ^o^C. In panel (B) an expansion of a NOESY spectrum (240 ms mixing time at 20 ^o^C) showing labelled intra- and inter-residual H1′-H6/H8 NOE dipolar couplings and sequential correlations denoted with lines.


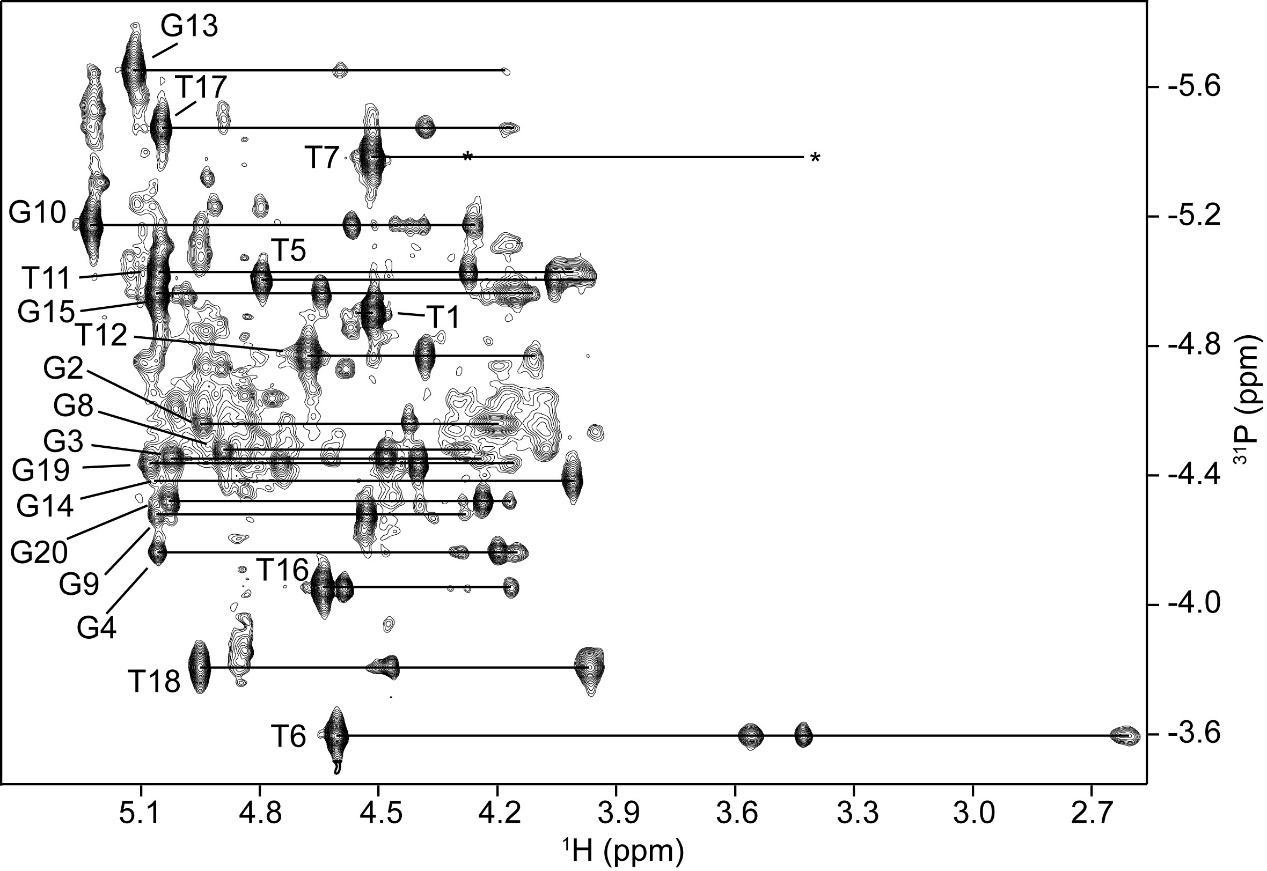


**Figure S3.** A [^31^P-^1^H] HSQC correlation spectrum showing ^31^P assignments for the d(TG_3_T_3_G_3_T_2_G_3_T_3_G_3_) DNA sequence (2MFU) formed in 80 mM NaCl, 20 mM NaPi, pH 6.8 and 20 °C.

**
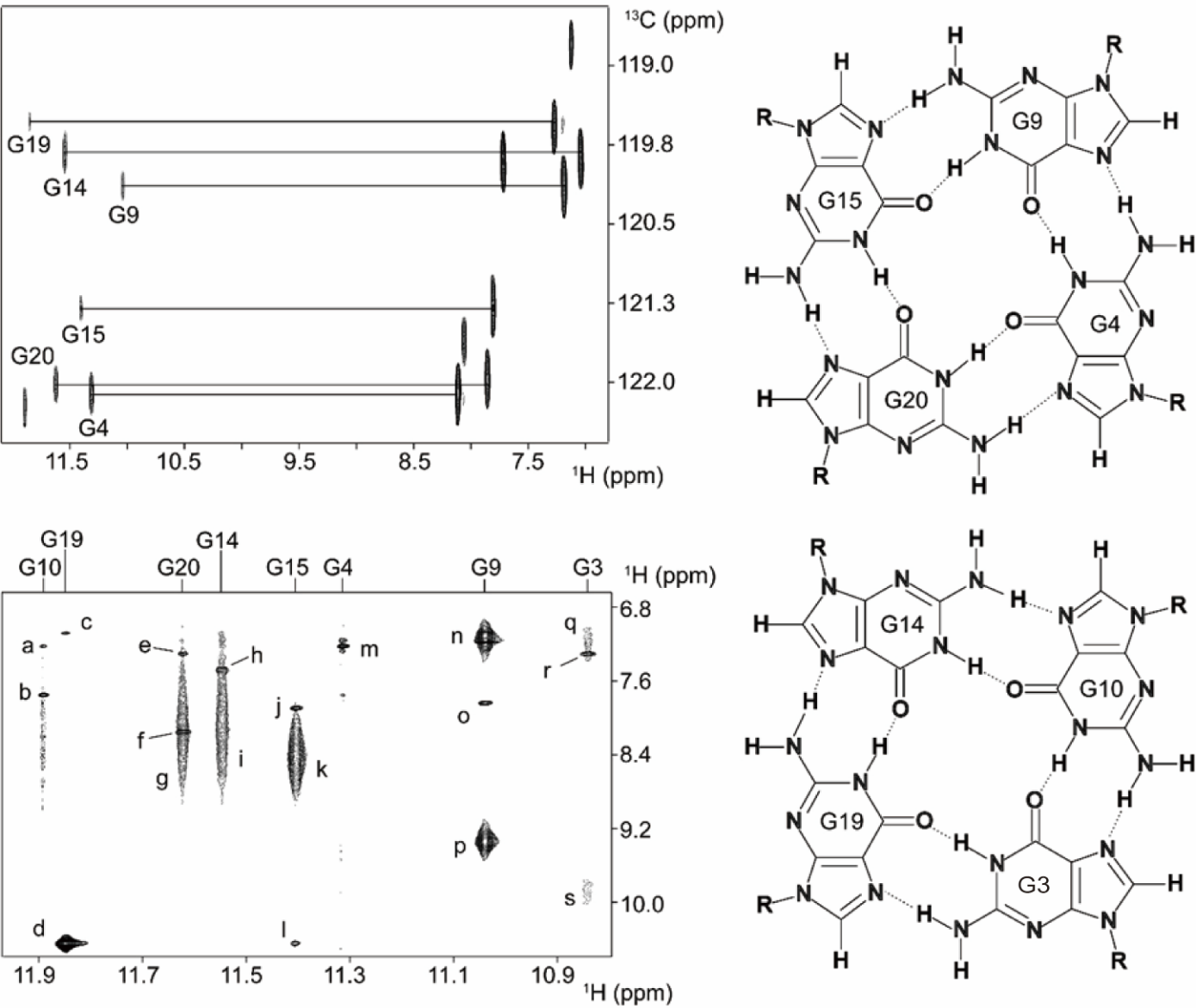
**

**Figure S4.** Exchangeable proton NMR assignments of d(TG_3_T_3_G_3_T_2_G_3_T_3_G_3_) (2MFU) in 80 mM NaCl and 20 mM NaPi buffer at pH 6.8 in ^1^H_2_O at 5 °C. Top spectrum expansion: Aromatic H8 proton to imino proton correlation (JR-HMBC) spectrum at 10 °C. This intraresidue correlation was used to identify individual guanine exchangeable protons from the assigned nonexchangeable aromatic protons. The through-bond correlation path involves the large (5-9 Hz) long-range scalar couplings between ^1^H imino to ^13^C C5, and between ^1^H H8-^13^C C5 pairs. Bottom spectrum: Expansion of NOESY spectrum (200 ms) of the exchangeable proton region. Peaks are assigned as follows: (a) G10H1-G9H8, (b) G10H1-G3H8 , (c) G19H1-G14 H8, (d) G19H1-xb NH2 , (e) G20H1-G19H8, (f) G20H1-G4H8, (g) G20H1-xc NH2 , (h) G14H1-G10H8, (i) G14H1-G14H21 , (j) G15H1-G20H8, (k) G15H1-G15H21, (n) G15H1-G19H21, (m) G4H1-G9H8, (n) G9H1-G9H21, (o) G9H1-G15H8, (p) G9H1-G9H22 , (q) G3H1-G3H22, (r) G3H1-G19H8, and (s) G3H1-G3H22.

**Description of 2MFU structure**

The 2-stacked, canonical quadruplex structure formed by d(TG_3_T_3_G_3_T_2_G_3_T_3_G_3_) bears two sequential lateral loops followed by a propeller; i.e., a 2(-L_w_-L_n_-P) topology- see **Figure 1**. In each strand, the stem consists of guanines adopting alternating *syn*G-*anti*G conformations. The first lateral loop is comprised of four residues, the segment TTTG, which includes the first guanosine of the second G-tract, G8. A guanosine of the third G-tract, is part of the second loop that is comprised of 3 residues, TTG. Only the final loop, a propeller type, is comprised of the 3 thymines as designed. The two remaining guanosines intended for the stem adopt 5’ and 3’ positions beyond the 2-stacked quadruplex stem: G2 and G21. The guanosine at the 5’-end of the stem adopts a *syn* glycosidic torsion angle, and is preceded by a thymine that forms stacking interactions with T18 of the third (propeller) loop and T12 from the second loop (-L_n_). These hydrophobic interactions cover a great portion of the (G3:G10:G14:G19) tetrad, although not stacking to it. Additional intrastrand stacking involving G13 to the same tetrad’s G14 further covers the potential surface exposed area of this tetrad. Guanine *syn*G8 in the first loop forms hydrogen bonds to *anti*G21 beyond the 3’ end of the stem, forming extensive stacking diagonally across the tetrad at the other end of the stem, (G4:G9:G15:G20).

**Modified oligonucleosides can be used to prevent frustrated folding**

Putative canonical G-quadruplex-folding sequences of non-selective loop-length combinations tend to result in the folding of several species; especially in the absence of purines in the putative loops. We have previously evaluated selectivity of loop length-combinations modulated by number of stacking tetrads in the folding of canonical G-quadruplexes.^[1]^ For that we operated under the working hypothesis that sodium solutions should allow for access of greater variety of topologies. Considering this premise, and the lack of purines in the putative loops of the sequence of d(G_3_T_4_G_3_T_4_G_3_T_4_G_3_), it is plausible to assume that it should result in frustrated folding in sodium solutions. However, the sequence already contains two sequential selective loops: a selective first putative loop of four thymines, and a selective putative second diagonal loop of four residues. This combination should result in a -Lw first loop, followed by a diagonal loop. This combination of loops 4-4-4 in sodium does indeed result in a mixture of species (see **Fig. S5A**), possibly containing frustrated folding. We can induce formation of the desired species by judicious inclusion of select modified oligonucleosides/tides. A second diagonal loop is formed if there are no pre-residues to the 5’-end of the stem- as shown in the PDB id 2M6V. Therefore the combination -Lw first loop, followed by a diagonal loop is selective if we ensure that the top tetrad is formed. In order to induce it to fold into the desired topology we utilized rG to induce formation of *anti*G for the bases in this tetrad- see **Fig. S5B**. Therefore, contrasting to 2M6V, here the first -Lw is formed with a selective loop-length.


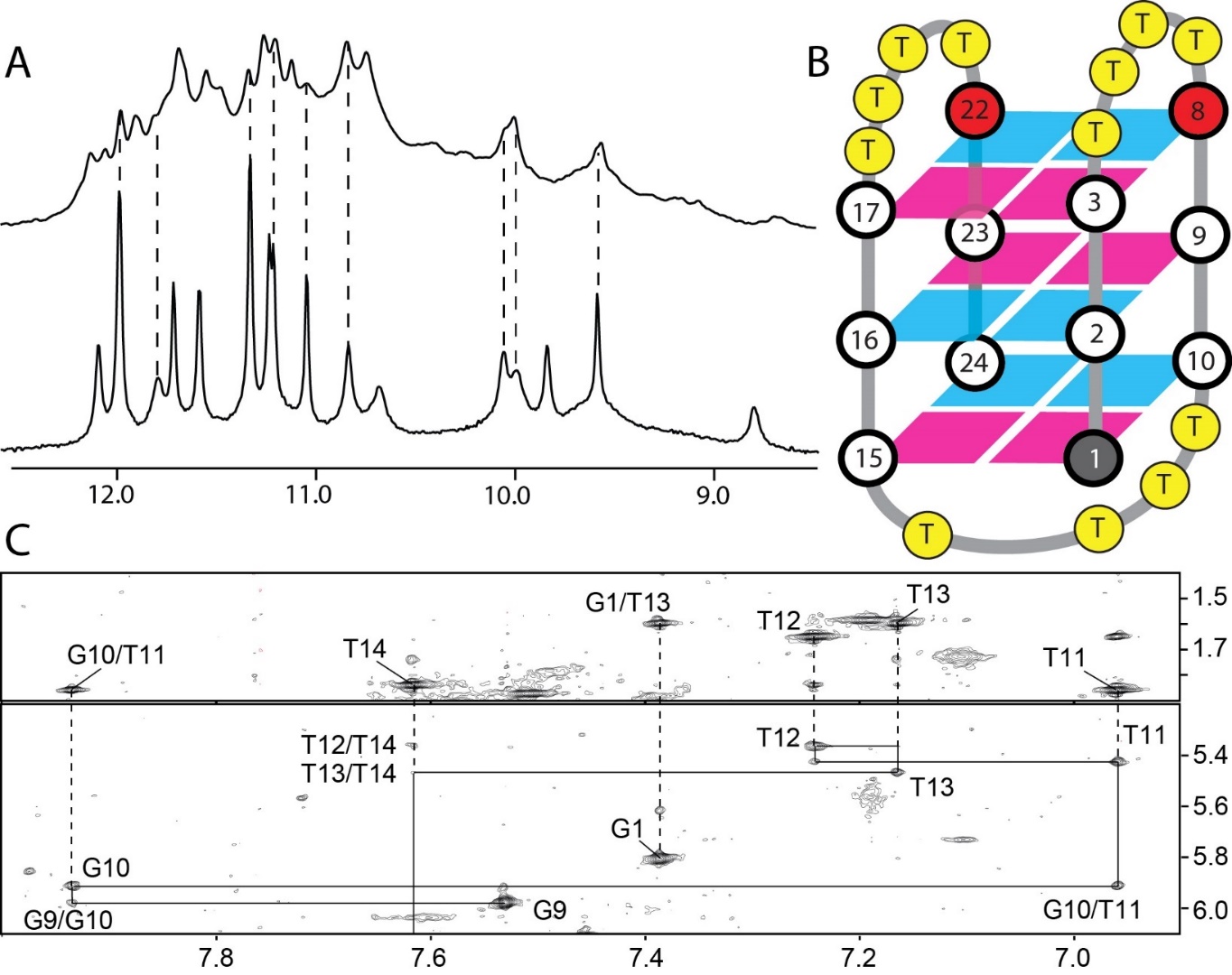


**Figure S5.** Combinations of four-residue long segments can be non-selective in canonical quadruplexes. In (A), imino region expansions of the proton NMR spectra in 0.1 M sodium solutions (pH 6.8) for d(G_3_T_4_G_3_T_4_G_3_T_4_G_3_) top, and d(G_3_T_4_rGG_2_T_4_G_3_T_4_rGG_2_) bottom. In (B) a schematic representation of the topology adopted by the sequence d(G_3_T_4_rGG_2_T_4_G_3_T_4_rGG_2_) in sodium solutions. Guanosines in tetrads comprising the quadruplex stem are displayed as pink (*syn*G) and cyan (*anti*G) squares, with numbered white (deoxyriboguanosine) or red (riboguanosine) circles indicating their position in the primary sequence. Thymines of each loop are shown as yellow circles. (C) Diagonal loop assignments for d(G_3_T_4_rGG_2_T_4_G_3_T_4_rGG_2_). Expansions of proton NOESY spectrum at 250 ms mixing time and 20 °C, illustrating the presence of the characteristic sequential connectivities (*syn*G9-*anti*G10-T11-T12-T13-T14) of the diagonal loop in the 3(-l_w_d+l_n_) topology.^[1]^ The characteristic connectivity between the aromatic H8 of the 5’-*syn*G1 residue of the stem, and the methyl of the third Thymine (T13) in the diagonal loop is illustrated. Considering these assignments, and the reduced number of imino signals, the rGs do not appear to be part of the stem. They however lock the conformation in an *anti* disposition, thus inducing unique architecture. This is possibly by contributing to the cap onto the top tetrad. The contribution of this species to the spectrum with unmodified guanosines is shown by the tracing lines.

# III. Structure calculations

Structures were calculated in XPLOR-NIH using NOE-derived distance/dihedral restraints (608 total). Distance constraints were derived from the volumes of NOE cross-peaks corresponding to the distance between H5–methyl in thymine residues (2.46 Å) in NOESY experiments in “100%” ^2^H_2_O. Exchangeable proton NOEs were categorized by intensity: strong (3.0 ± 0.8 Å), medium (4.0 ± 1.2 Å), weak (5.0 ± 1.8 Å). Simulated annealing generated ensembles, from which the 10 lowest-energy structures are reported (**Table S1**). Coordinates are deposited in the PDB (2MFU) and chemical shifts in BMRB (19572).

## **Table S1**. NMR restraints and structural statistics for 10 selected structures for the unimolecular d(TG_3_T_3_G_3_T_2_G_3_T_3_G_3_) quadruplex.

| NMR Restraints | |
| --- | --- |
| total number of restraints | 608 |
| nonexchangeable protons | 466 |
| exchangeable protons | 142 |
| Intraresidue | 283 |
| sequential (i, i+1) | 116 |
| hydrogen bond restraints (empirical) | 36 |
| Structural Statistics | |
| NOE violations exceeding 0.2 Å | 0 |
| deviations from ideal covalent geometry, rmsd | |
| bond lengths (Å) | 0.016 ± 0.001 |
| bond angles (deg) | 0.638 ± 0.006 |
| impropers (deg) | 0.317 ± 0.006 |
| pairwise all heavy atoms rmsd (Å) | 1.10 ± 0.18 |

# IV. SRCD spectroscopy

SRCD spectra were recorded at the B23 beamline, Diamond Light Source (Module B). Single-scan measurements used 40 μL samples in 0.02 cm path-length cuvettes, with 1 s integration time, 1 nm bandwidth, and 1 nm pitch. Spectra (185–330 nm) were collected between 4.5–90 °C, with ~5 °C increments up to 40 °C and ~7 °C thereafter. After heating to 90 °C, samples were cooled to 4.5 °C and spectra re-acquired; pre- and post-heating spectra overlapped within noise, confirming reversibility (no aggregation or photodamage). Buffer baselines were subtracted. Instrument details follow Hussain et al..^[2, 3]^ Final spectra were converted to Δε (M⁻¹·cm⁻¹) via Δε = θ/(32,980·l·C). See **Fig. S6** for reversibility data. To ensure that the temperature-resolved spectra report equilibrium populations, CD spectra were acquired using four times the dwell time/step size protocol as for SRCD. The extracted state spectra and population profiles (including the S₂ basin) were invariant within experimental uncertainty, indicating that the decomposition reflects equilibrium rather than scan-rate–dependent kinetic trapping.

**Thermodynamics Calculations- main parameters**

Thermodynamics parameters were calculated from thermal data using Van’t Hoff equation derivatization (Eq. 1).

$\Delta G= -RTln\left( Keq \right)= \Delta H- T\Delta S$ Eq. 1

where $\Delta G$ is the free Gibbs energy in joules, $R$ is the ideal gas constant (8.3145 *J.mol^-1^.K^-1^*), $T$ is the temperature in Kelvin, $Keq$ is the equilibrium constant, $\Delta H$ is the change in enthalpy, and $\Delta S$ is change in entropy. Firstly, we evaluated the $Keq$ using Eq. 2.

$Keq= \frac{F}{1-F}$ Eq. 2

where *F* is the folded fraction and was calculated using Eq. 3 while (*1 – F*) is the unfolded fraction.

$F= \frac{[\theta t-\theta u]}{[\theta f-\theta u]}$ Eq. 3

where $\theta t$ is the molar ellipticity at temperature T, while $\theta u$ and $\theta f$ are the molar ellipticity at unfolded and folded states, respectively. We calculated $\Delta H$ and $\Delta S$ by plotting *ln(Keq)* against 1/T using Eq. 4&5, respectively.

$\Delta H= -R\times slope$ Eq. 4

$\Delta S=R\times intercept$ Eq. 5

Since the calculated $\Delta H$ and $\Delta S$ are for the folding, the negatives of the values were used for the unfolding. As $\Delta G$ = 0 at melting temperature ($Tm)$, we calculated $Tm$ using Eq. 6.

$Tm= \frac{\Delta H}{\Delta S}$ Eq. 6

**Kinetics Calculations**

Arrhenius equations were used for the analysis. We plotted the natural logarithm of the unfolding reaction constant (lnKu) against the inverse of the temperatures in Kelvin (1/T) where the unfolding reaction constant (Ku) was calculated from the fraction of unfolded using first order reaction kinetics. Eq. 7 represents the plotted line in Arrhenius plot.

$lnku=-\frac{Ea}{RT}+lnA$ Eq. 7

where activation energy ($Ea$) was calculated from the slope using Eq. 8.

$Ea=-R*slope$ Equation

**Global three-state thermodynamics from multi-wavelength SRCD (2MFU)**

SRCD spectra (260-300 nm) acquired at 12 temperatures (4.5-90 °C) were fit globally to a sequential three-state equilibrium $F\rightleftharpoons I \rightleftharpoons U$. State populations were parameterized by van’t Hoff relations with $\Delta C_{p}= 0$:

$K_{12}\left( T \right)=e^{-\Delta G_{12}\left( T \right)/RT},$ $K_{23}\left( T \right)=e^{-\Delta G_{23}\left( T \right)/RT},$ $\Delta G_{ij}\left( T \right)=\Delta H_{ij}^{\circ}\left( 1-\frac{T}{T_{m,ij}} \right)$,

and constrained to $p_{F} = \frac{1}{Z}$ , $p_{I} = \frac{K_{12}}{Z}$ , $p_{U} = \frac{K_{12}K_{23}}{Z}$ with $Z = 1 + K_{12} + K_{12}K_{23}$.

Each observed spectrum was modeled as a temperature-independent linear combination of state spectra $\theta_{s}\left( \lambda\right)$:

$$\theta_{\text{obs}}\left( \lambda,T \right)=p_{F}\left( T \right)\theta_{F}\left( \lambda\right)+p_{I}\left( T \right)\theta_{I}\left( \lambda\right)+p_{U}\left( T \right)\theta_{U}\left( \lambda\right).$$

Temperatures were treated in Kelvin; $R = 8.314 J \mathrm{mol}^{-1} K^{-1}.$ Parameters $\{T_{m,12}, \Delta H_{12}^{\circ}, T_{m,23}, \Delta H_{23}^{\circ}\}$ and the state spectra $\{\theta_{F}\left( \lambda\right), \theta_{I}\left( \lambda\right), \theta_{U}\left( \lambda\right)\}$ were estimated by least squares over all ($\lambda, T)$ (no spectral smoothing; weights uniform). Uncertainty was assessed by bootstrap resampling of wavelengths (B=300): for each resample we re-estimated $\{T_{m,23}, \Delta H_{23}^{\circ}\}$ fixed to the best fit (robustly determined by the low-T branch), then recomputed $p_{s}\left( T \right)$ and derived quantities.

From the fitted populations we report:

(i) Full unfolding free energy $\Delta G_{\text{unf}}^{\circ}\left( T \right)=-RT\ln\left[ p_{U}\left( T \right)/p_{F}\left( T \right) \right];$

(ii**)** “Native vs rest” stability $\Delta G_{\text{fold(nat vs rest)}}^{\circ}\left( T \right)$ = $-RT\ln\left[ p_{F}\left( T \right)/\left( p_{I}\left( T \right)+p_{U}\left( T \right) \right) \right]$.

For visualization of temperature-dependence independent of bin width we plot the thermodynamic derivative $\Delta G / \Delta T$ between adjacent temperatures (K-normalized), with 95% Cls from the bootstrap.

**Data used**. The 260–300 nm window was chosen to maximize separation of *F*/ *I*/ *U* spectral features and avoid high-noise deep-UV. Baselines and buffer subtraction followed the beamline protocol in Section IV; reversibility was confirmed by pre/post-heating overlays.

**Free energy values of the main species and the intermediate**. Main species (native, S1) at 25 °C: ΔG_F vs U_​≈ −7.49 kJ/mol (“Unfolding” free energies are the same magnitudes with opposite sign, e.g., ΔGu_nfolding_(F)= +7.49 kJ/mol at 25 °C); Intermediate (S2) at 25 °C: ΔG_I vs U_​≈ −3.11 kJ/mol; Intermediate relative to the native: ΔG_I vs F_​≈ +4.38 kJ/mol. These ΔG values were calculated from the 25 °C state populations returned by the global three-state thermodynamic fit to the multi-wavelength SRCD data in the 260–300 nm window, using representative fitted populations pF ≈ 0.82, pI ≈ 0.14, and pU ≈ 0.04. Here, SVD/PCA were used only to support the low-rank dimensionality of the dataset and the presence of an additional intermediate-associated contribution; they were not used to define the physical state spectra or to extract the thermodynamic populations used for ΔG calculation. This was computed through ΔG= −RT ln(*p*_A_​/*p*_B_​). ΔG values derived from population ratios assume a single-strand stoichiometry across species and dilute solution. The temperature-dependence of these fitted state populations, together with the derivative stability of the native relative to the rest, is shown in **Fig. S6**, highlighting that 2MFU unfolds sequentially through an intermediate rather than by a simple two-state transition. These experimentally derived ΔG values at 25 °C (for F and the S₂/I intermediate relative to U) were used as anchoring constraints for affine rescaling of the WT‑BE‑MetaD free‑energy surfaces (see Section VII, “CD‑Anchored Rescaling of Free‑Energy Surfaces”).

**Effective two-state description of 2JSL under the present SRCD conditions**

Under our experimental conditions the thermal unfolding of 2JSL was adequately described by a two‑state model; this does not imply that the sequence is universally two‑state, as the literature documents condition‑dependent complexity.

For 2JSL, spectra (260-300 nm) were fit using an effective two-state description under the present SRCD conditions $F\rightleftharpoons U$ with temperature-independent $\theta_{F}\left( \lambda\right), \theta_{U}\left( \lambda\right)$ and van’t Hoff dependence $\Delta G\left( T \right) ={\Delta H}^{\circ}(1 - T/T_{m})$ $\left( {\Delta C}_{p}= 0 \right)$. Populations, $\Delta G_{\text{unf}}^{\circ}\left( T \right)$, and 95% Cls were obtained analogously by wavelength bootstrap. Free energy values of the folded and unfolded species were derived by a two-state van’t Hoff analysis of the 290 nm CD melting profile of 2JSL. At 25 °C, the folded/native state is stabilized relative to the unfolded baseline with ΔG_F vs U_ ≈ −7.3 kJ·mol⁻¹ (correspondingly, ΔG_unfolding(F) ≈ +7.3 kJ·mol⁻¹ at 25 °C). These values were obtained from the population ratio at room temperature (p_F_ ≈ 0.81, p_U_ ≈ 0.19), using the relation ΔG = −RT ln(*p*_A_/*p*_B_). The fitted parameters from this effective two-state Greenfield model gave T_m_ ≈ 65.4 °C and an apparent van’t Hoff enthalpy ΔH_vH_ ≈ 186.5 kJ·mol⁻¹. As in the standard approach, the calculation assumes a single-strand stoichiometry across species, dilute solution conditions, and ΔC_p_ = 0.


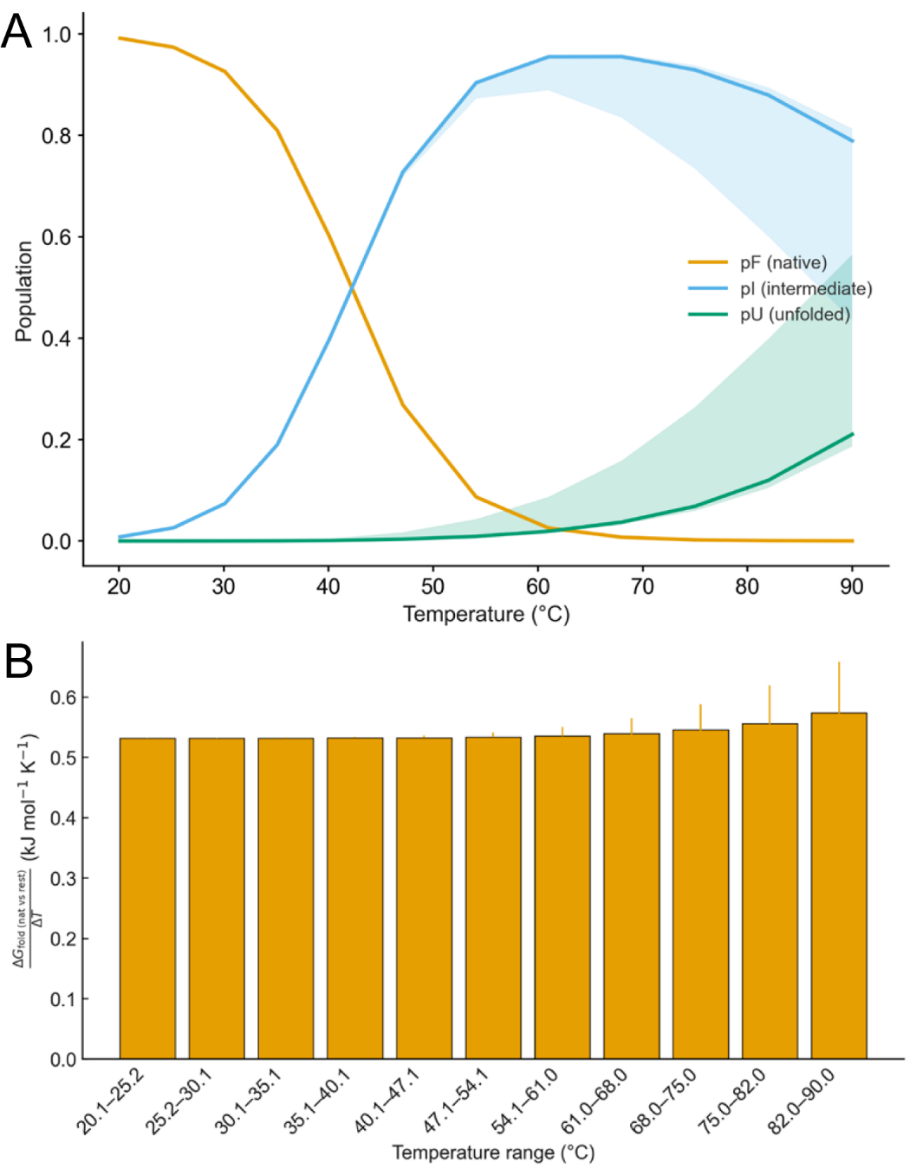


**Figure S6**. Evaluation of equilibrium thermodynamics of 2MFU from global multi-wavelength CD (260–300 nm): state populations and temperature-derivatives of ΔG. Native stability decreases sequentially via an intermediate, underscoring a non-two-state free-energy landscape for 2MFU. (A) *Populations vs temperature*. Native $\left( p_{F} \right)$, intermediate $\left( p_{I} \right)$, and unfolded $\left( p_{U} \right)$ populations obtained by a global three-state sequential model $F\rightleftharpoons I \rightleftharpoons U$ fit to CD spectra collected at 12 temperatures (Kelvin in the model; plotted in °C). State spectra $\theta_{S}\left( \lambda\right)$ are temperature-independent; population temperature-dependence follows van’t Hoff with $\Delta C_{p}= 0$: $K_{12}\left( T \right)=e^{-\Delta G_{12}\left( T \right)/RT},$ $K_{23}\left( T \right)=e^{-\Delta G_{23}\left( T \right)/RT},$ $\Delta G_{ij}\left( T \right)=\Delta H_{ij}^{\circ}\left( 1-\frac{T}{T_{m,ij}} \right)$. Shaded envelopes show 95% bootstrap Cls (resampling wavelengths, B = 300; re-fitting $T_{m,23}$ and $\Delta H_{23}^{\circ}$ on a coarse grid; the first step is fixed to the well-determined values $T_{m,12}= 315.25 K$ and $\Delta H_{12}^{\circ}= 167.5 KJ {mol}^{-1}$). (B) *Thermodynamic derivative* (*native vs rest*). Window-wise slope of the “native vs rest” stability, ${\Delta G}_{\text{fold(nat vs rest)}}\left( T \right)$ = $-RT\ln\left[ p_{F}\left( T \right)/\left( p_{I}\left( T \right)+p_{U}\left( T \right) \right) \right]$, reported as ${\Delta G}_{\text{fold(nat vs rest)}}/\Delta T (KJ {mol}^{-1}K^{-1})$ across adjacent temperature windows. Orange bars show the mean values; vertical yellow error bars represent 95% bootstrap confidence intervals obtained by resampling wavelengths (B=300) and re-fitting values $T_{m,23}= 315.25 K$ and $\Delta H_{23}^{\circ}= 167.5 KJ {mol}^{-1}$).

**Dimensionality reduction**

Multivariate analyses were performed on buffer-subtracted CD spectra (205–330 nm, normalized to Δε). For PCA, spectra were mean-centered and z-scored. PCA ^[4]^ was performed with prcomp (R 4.4.2). t-SNE ^[5]^ used Rtsne (v0.17; seed = 42, perplexity = 2, θ = 0.2). k-means clustering (seed = 42) on PC1–PC2 scores resolved k = 3 states for 2MFU and k = 2 for 2JSL. In parallel, spectral decomposition used truncated SVD^[6]^ (scikit-learn v1.3.2) applied directly to the buffer-subtracted Δε matrices over 205–330 nm. For 2MFU the retained rank was 3; for 2JSL the retained rank was 2. For comparison of individual spectra with the retained low-rank subspace, each measured spectrum was projected onto the retained components; where population-like weights are shown, spectra were further fitted as non-negative linear combinations of the retained components and renormalized to sum = 1. These low-rank weights are descriptive coordinates within the retained spectral subspace and should not be interpreted as thermodynamic state populations from the global three-state model. For overlays, TD-DFT/ECD spectra were affinely aligned to the 205–330 nm window (λ′ = sλ + Δ; intensities not fitted). Diagnostic plots for scree behavior, first-order autocorrelation, and rank-truncated reconstruction/residual analyses are provided below.


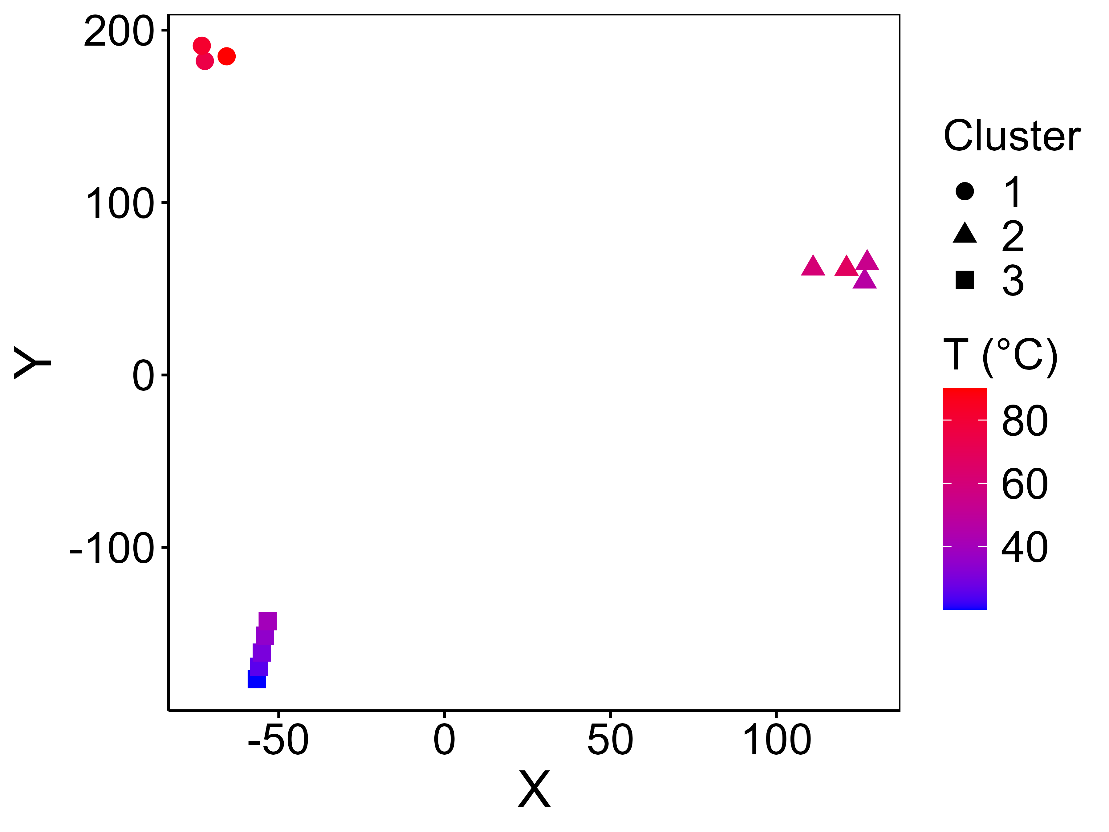


**Figure S7.1.** Unfolding intermediates of 2MFU resolved by t-SNE clustering of dichroic spectra. Circular dichroism spectra were projected using t-SNE and grouped by K-means clustering (seed fixed at 42, as in the PCA analysis), identifying three ensembles across the temperature range 4.5–90 °C: S1 (folded), S2 intermediate-associated ensemble, and S3 (unfolded). Temperature increments were ~5 °C between 4.5 and 40.1 °C and ~7 °C thereafter. Fixing the seed ensured reproducible cluster representation; only the graphical appearance of clusters differs from earlier plots, without altering the results.

**SVD rank-selection, autocorrelation, and reconstruction diagnostics**

To document the dimensionality choices used in **Figure 2**, additional diagnostics were evaluated for the truncated SVD decomposition of the buffer-subtracted SRCD matrices (205–330 nm). Scree plots of the singular values and explained variance showed a clear elbow after the third component for 2MFU and after the second component for 2JSL. For 2MFU, the first five singular values were 760.16, 300.46, 92.41, 33.25, and 24.29, corresponding to 86.62, 10.51, 2.24, 0.29, and 0.16% of the variance, respectively; the retained rank-3 model captured 99.37% of the total variance. For 2JSL, the first five singular values were 1991.22, 286.10, 51.40, 33.84, and 19.80, corresponding to 93.09, 6.39, 0.27, 0.12, and 0.04% of the variance; the retained rank-2 model captured 99.48% of the total variance.

First-order (lag-1) autocorrelation coefficients were computed for both the temperature-side singular vectors and the wavelength-side singular vectors as r1 = corr(x1:n-1, x2:n). For 2MFU, the temperature-side coefficients for components 1–5 were 0.978, 0.975, 0.595, 0.368, and 0.371; for 2JSL they were 0.970, 0.967, 0.687, 0.485, and −0.390. The wavelength-side coefficients remained high for all low-order components (2MFU: 0.993, 0.985, 0.984, 0.962, 0.966; 2JSL: 0.996, 0.975, 0.980, 0.970, 0.901), as expected for smooth SRCD basis vectors. Thus, autocorrelation supports the retained dimensionality but is not used in isolation; the primary justification is the combined scree, variance-capture, and reconstruction behaviour.

Reconstruction tests were performed by projecting the experimental matrices onto the retained subspace and comparing rank-truncated reconstructions with the observed data. For 2MFU, rank 3 gave RMSE = 1.26 Δε, relative Frobenius error = 0.059, mean absolute residual = 0.96 Δε, and maximum absolute residual = 5.27 Δε. For 2JSL, rank 2 gave RMSE = 1.85 Δε, relative Frobenius error = 0.036, mean absolute residual = 1.35 Δε, and maximum absolute residual = 7.16 Δε. Across temperatures, the mean residual remained close to zero (within ±0.60 Δε for 2MFU and ±0.37 Δε for 2JSL). The residual matrices do retain local smoothness across wavelength, as expected for spectroscopic data, but they do not reveal omitted high-amplitude bands or an additional temperature-dependent transition. Together, these diagnostics support the use of rank 3 for 2MFU and rank 2 for 2JSL in **Figure 2**. Details are shown in **Figures S7.2-7.7** below.


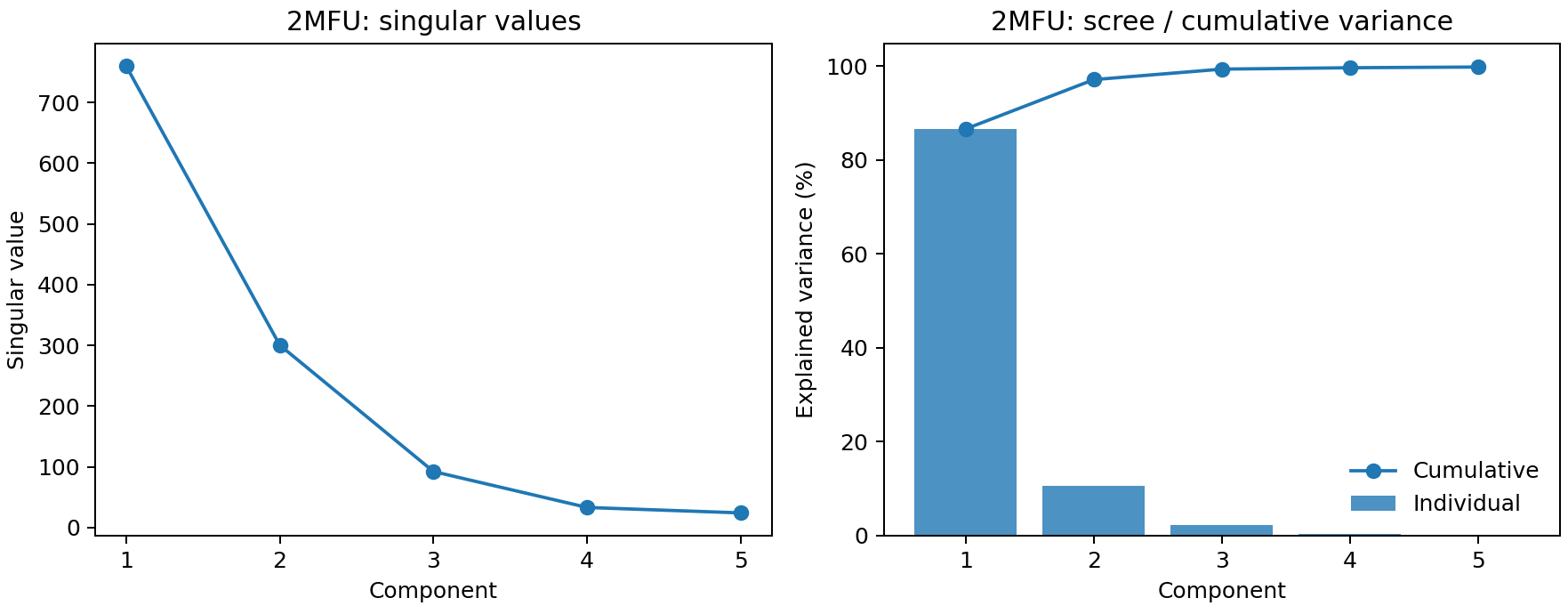


## **Figure S7.2**. Scree plot and cumulative explained variance for 2MFU.

Truncated-SVD diagnostics for the buffer-subtracted 2MFU SRCD matrix (205–330 nm). Left, singular values for the first five components. Right, individual explained-variance contributions (bars) and cumulative explained variance (line). Components 1–3 account for 86.62, 10.51, and 2.24% of the variance (99.37% cumulative), whereas components 4–5 contribute only 0.29 and 0.16%, supporting retention of three components.


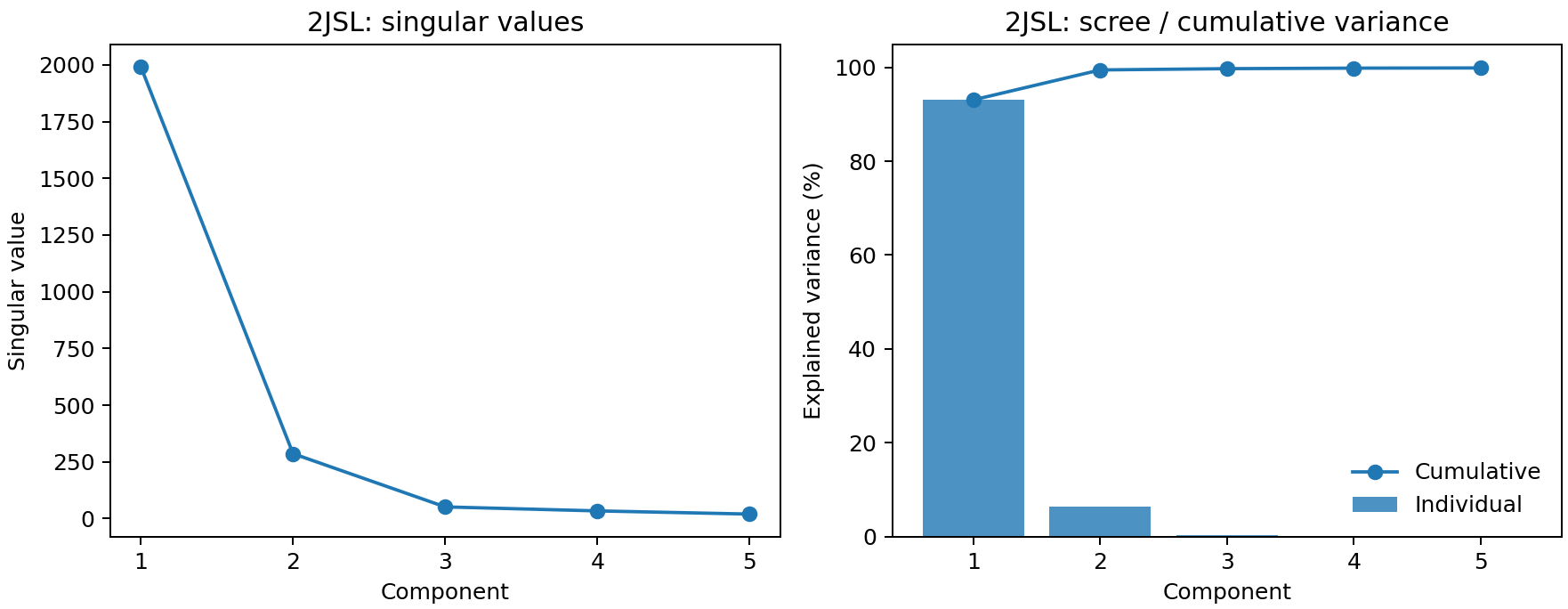


## **Figure S7.3**. Scree plot and cumulative explained variance for 2JSL.

Truncated-SVD diagnostics for the buffer-subtracted 2JSL SRCD matrix (205–330 nm). Left, singular values for the first five components. Right, individual explained-variance contributions (bars) and cumulative explained variance (line). Components 1–2 account for 93.09 and 6.39% of the variance (99.48% cumulative), whereas components 3–5 contribute only 0.27, 0.12, and 0.04%, supporting retention of two components.


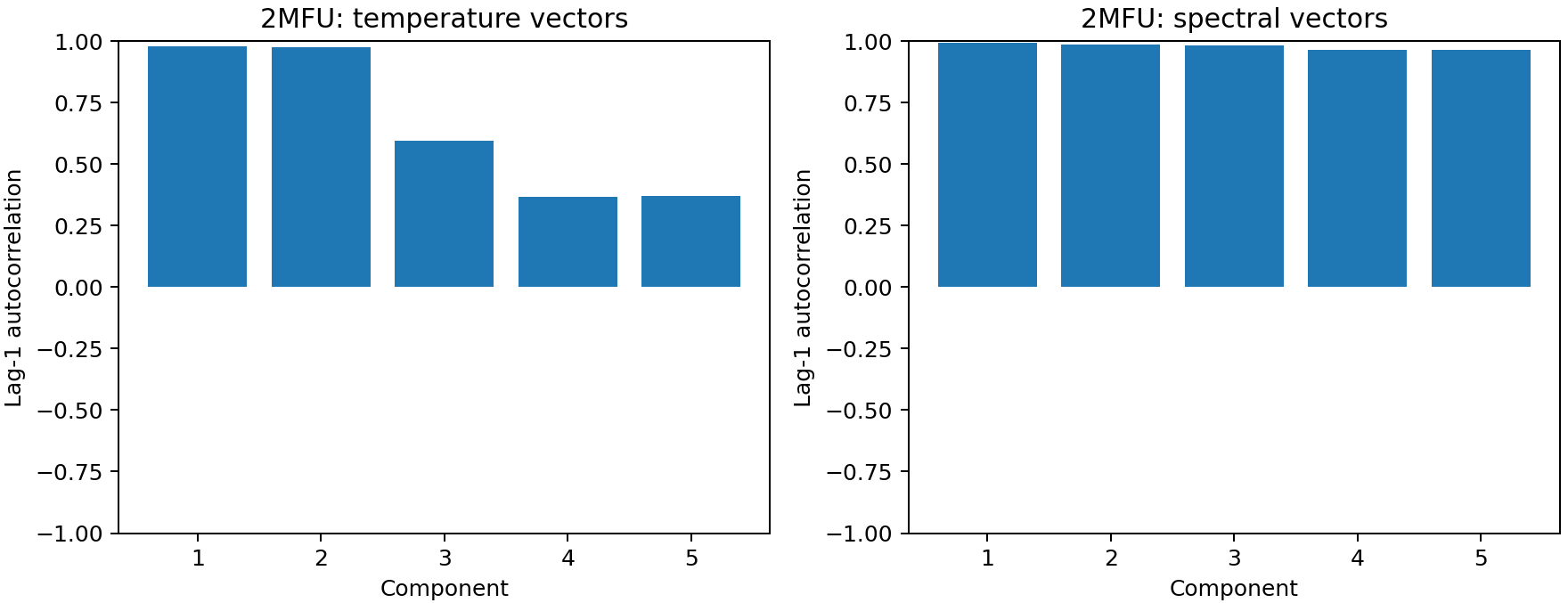


## **Figure S7.4**. Lag-1 autocorrelation coefficients for SVD vectors of 2MFU.

First-order autocorrelation coefficients calculated for the first five SVD vectors of 2MFU. Left, temperature-side singular vectors. Right, wavelength-side singular vectors. The marked reduction in temperature-vector autocorrelation after the retained low-order components is consistent with a three-component description. High wavelength-vector autocorrelation is expected because SRCD basis vectors are intrinsically smooth functions of wavelength.


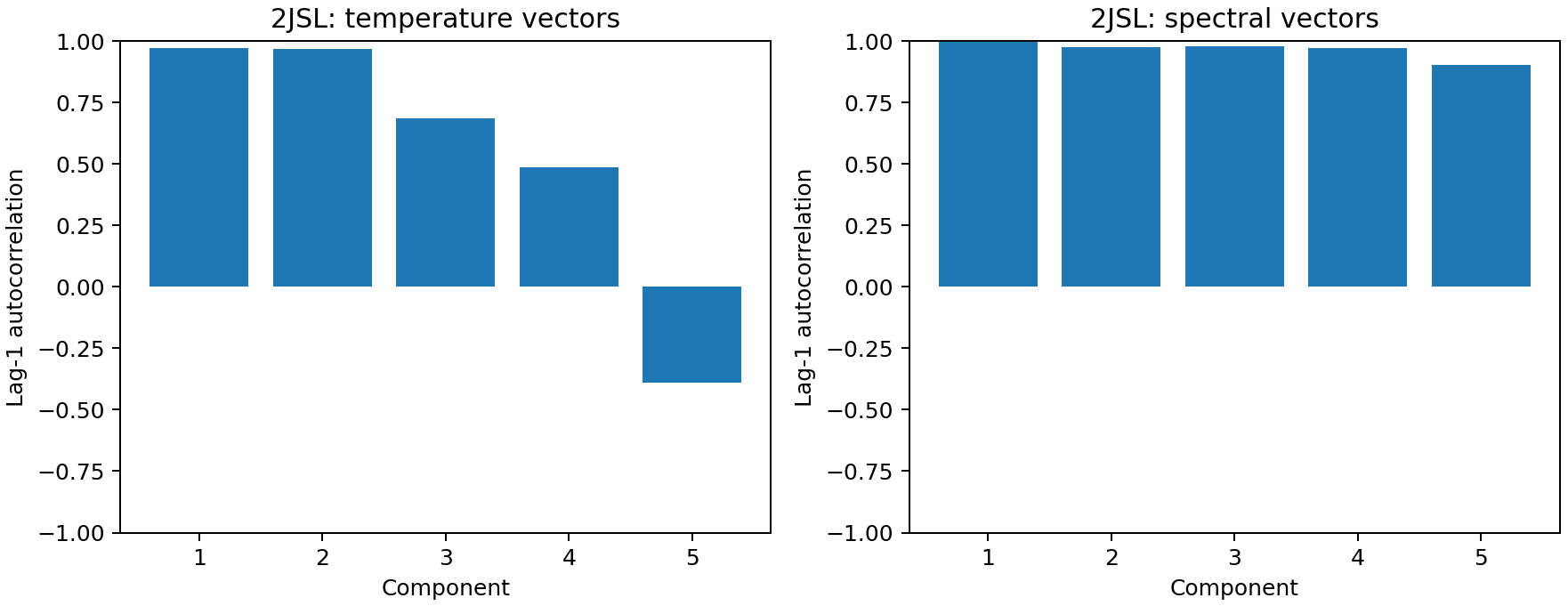


## **Figure S7.5**. Lag-1 autocorrelation coefficients for SVD vectors of 2JSL.

First-order autocorrelation coefficients calculated for the first five SVD vectors of 2JSL. Left, temperature-side singular vectors. Right, wavelength-side singular vectors. The dominant contribution of the first two components and the drop in temperature-vector autocorrelation beyond these components are consistent with a two-component description. As for 2MFU, wavelength-vector autocorrelation remains high because the spectral basis vectors are smooth over wavelength.


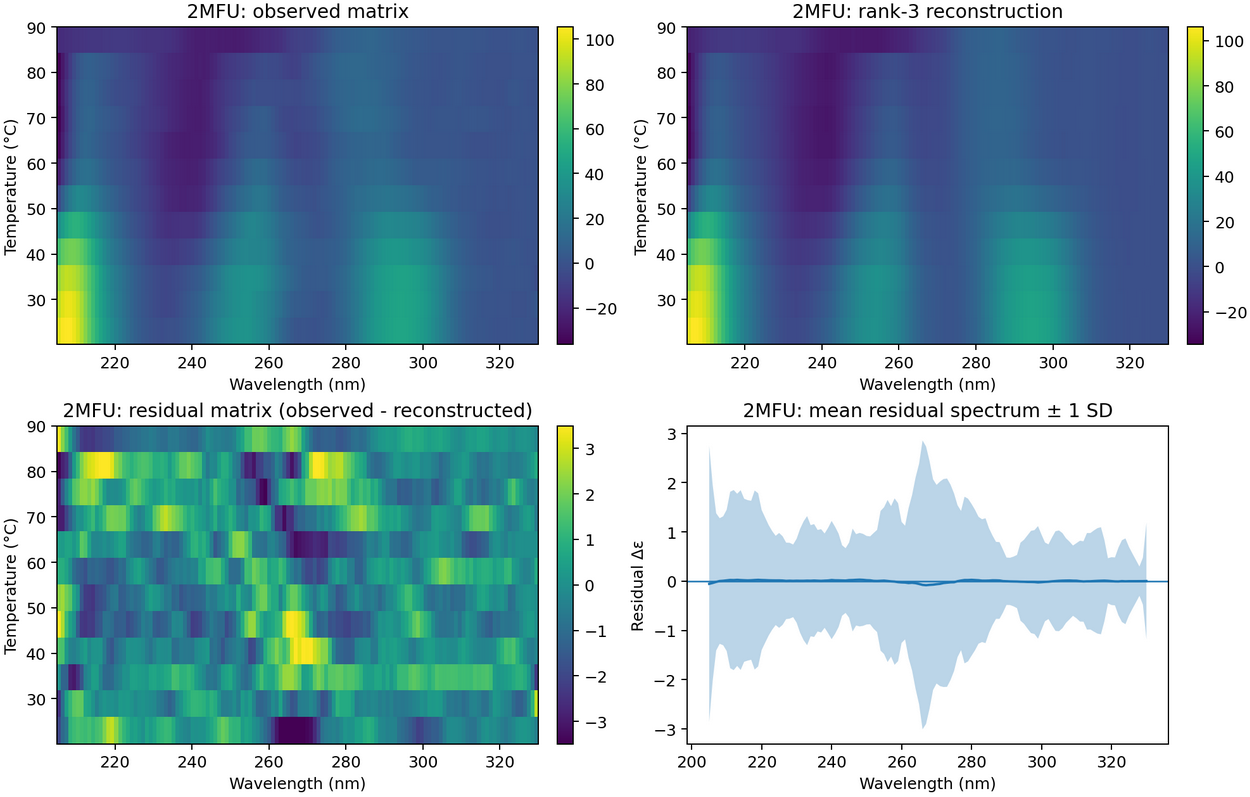


## **Figure S7.6**. Rank-3 reconstruction and residual analysis for 2MFU.

Upper left, observed 2MFU SRCD matrix (205–330 nm; 20.1–90.0 °C). Upper right, rank-3 reconstruction obtained from the retained SVD subspace. Lower left, residual matrix (observed − reconstructed). Lower right, mean residual spectrum ± 1 SD across temperature. The rank-3 model reproduces the major spectral evolution with low error (RMSE = 1.26 Δε; relative Frobenius error = 0.059), and the residuals do not reveal any omitted high-amplitude band or additional thermal transition.


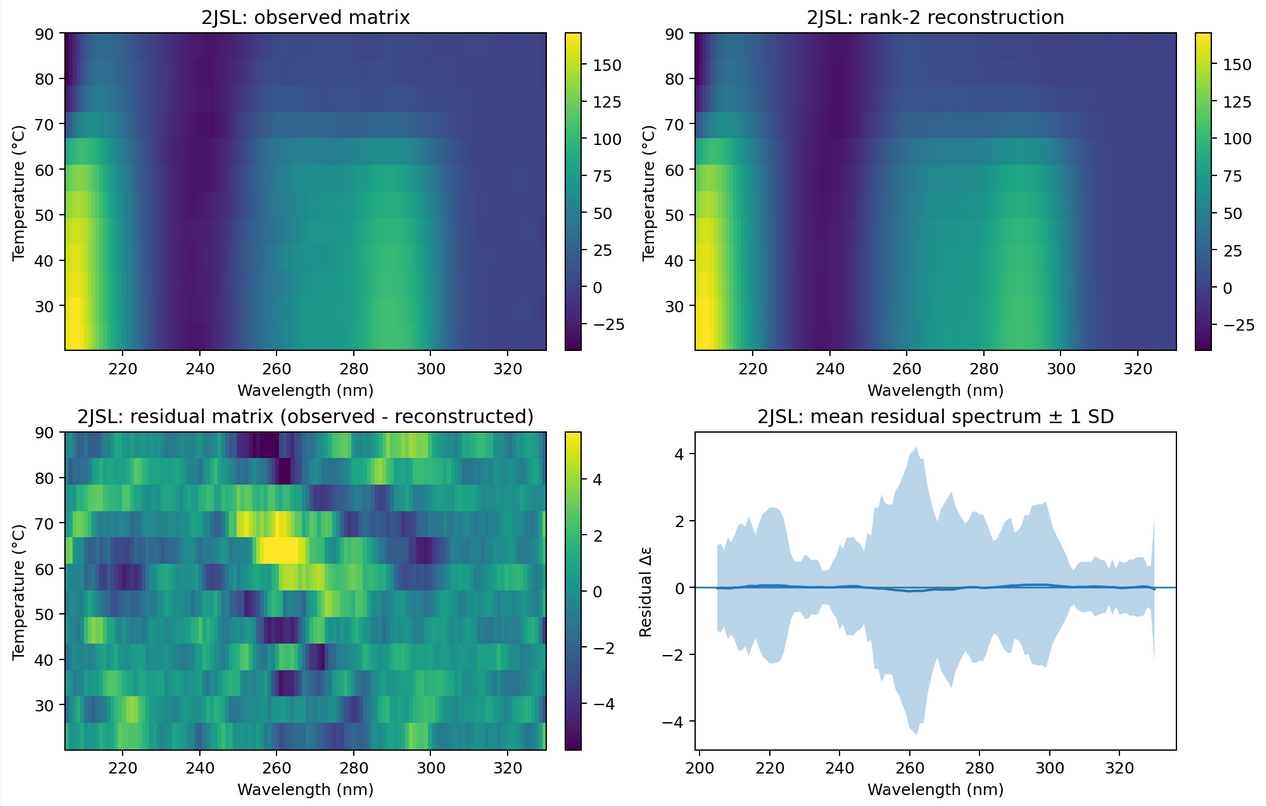


## **Figure S7.7**. Rank-2 reconstruction and residual analysis for 2JSL.

Upper left, observed 2JSL SRCD matrix (205–330 nm; 20.1–90.0 °C). Upper right, rank-2 reconstruction obtained from the retained SVD subspace. Lower left, residual matrix (observed − reconstructed). Lower right, mean residual spectrum ± 1 SD across temperature. The rank-2 model reproduces the major spectral evolution with low error (RMSE = 1.85 Δε; relative Frobenius error = 0.036), and the residuals do not reveal any omitted high-amplitude band or additional thermal transition.

# V. Standard MD simulations

**System preparation.** Simulations began from the lowest-energy conformer of the NMR ensemble of 2MFU. DNA was parameterized with the AMBER OL21 force field (parm99bsc0^[7]^ backbone with χ_OL4_^[8]^, ε/ζ_OL1_^[9]^, β_OL1_^[10]^, α/γ_OL21_^[11]^ refinements), solvated with TIP3P water in a truncated octahedral box (≥25 Å clearance). Ions were added to neutralize and reach 100 mM NaCl, using Joung–Cheatham parameters^[12]^ (3 channel Na⁺, 45 peripheral Na⁺, 28 Cl⁻). System sizes and compositions are summarized in **Table S1**.

**Minimization and equilibration.** Following steepest-descent/conjugate-gradient minimization (Amber^[13]^; restraints 10 kcal·mol⁻¹·Å⁻²), systems were converted to GROMACS 2022.5^[14]^ via ParmEd.^[15]^ Further minimization (10,000 steps) preceded heating from 10→300 K (1 ns, NVT, v-rescale thermostat).^[16]^ Restrained NPT equilibration followed (4 ns; Berendsen thermostat/barostat).^[17]^ Production used 3 × 300 ns replicates (NPT, 300 K, Parrinello–Rahman^[18]^, 2 fs timestep, PME^[19, 20]^ cutoff 10 Å).

**Trajectory analysis.** Global stability was assessed via RMSD, eRMSD^[21]^, Rg, and SASA^[22]^. Local structure was monitored via glycosidic bond angle distributions^[23]^ and π-stacking distances. All analyses used GROMACS^12^, CPPTRAJ^[13]^, MDTraj^[24]^, and Barnaba^[25]^; plots were generated with Matplotlib/Seaborn.^[26, 27]^ All three independent replicates showed equivalent behavior, with the G-stem remaining stable (RMSD < 2.5 Å) and loop residues (e.g., T11, T16, T18) exhibiting higher flexibility (RMSF up to 2.25 Å). A representative analysis is shown in **Fig. S8.1, S8.2**.

**Table S2.** Summary of simulation systems utilized in standard MD, well-tempered bias-exchange metadynamics (WT-BEMetaD), and pre-DFT empirical MD refinements, DFT geometry refinements. Parameters include system composition, solvation, forcefields, basis sets, and ion placement in both MD and quantum chemistry protocols.

| Task | Initial Structure | Forcefield / Basis set | Distance to Box Boundary**^b^ | Salt Conc. | DNA Atom | | Water mol. | Central Cation | Peripheral Cation | Anion |
| --- | --- | --- | --- | --- | --- | --- | --- | --- | --- | --- |
| Standard MD & WT-BEMetaD | G-quadruplex DNA 2MFU | OL21, TIP3P water model, Joung-Cheatham | 25 Å, Octahedral | 100mM NaCl | | 683 | 13498 | 3 Na^+^* | 45 Na^+^ | 28 Cl- |
| Pre-DFT empirical MD refinement | G-quadruplex DNA 2MFU |  | 10 Å, Octahedral | 100mM NaCl | | 683 | 2814 | 3 Na^+^* | 26 Na^+^ | 5 Cl- |
| DFT geometry refinement | 11 Guanosines with K^+^ 2MFU | def2-TZVP56, CAM-B3LYP, RIJCOSX, def2/J, D3BJ, CPCM implicit water model | x | x | | 176 | x | 3 K^+^ | x | x |
|  | 8 Guanosine with K^+^ 2MFU |  | x | x | | 128 | x | 3 K^+^ | x | x |
|  | 11 Guanosine with Na^+^ 2MFU |  | x | x | | 176 | x | 3 Na^+^ | x | x |
|  | 8 Guanosine with Na^+^ 2MFU |  | x | x | | 128 | x | 3 Na^+^ | x | x |

***** 1 central cation Na^+^ escapes from the central channel to the outside of the stem in the post-equilibrium system.

****** Under the periodic boundary conditions (PBC)


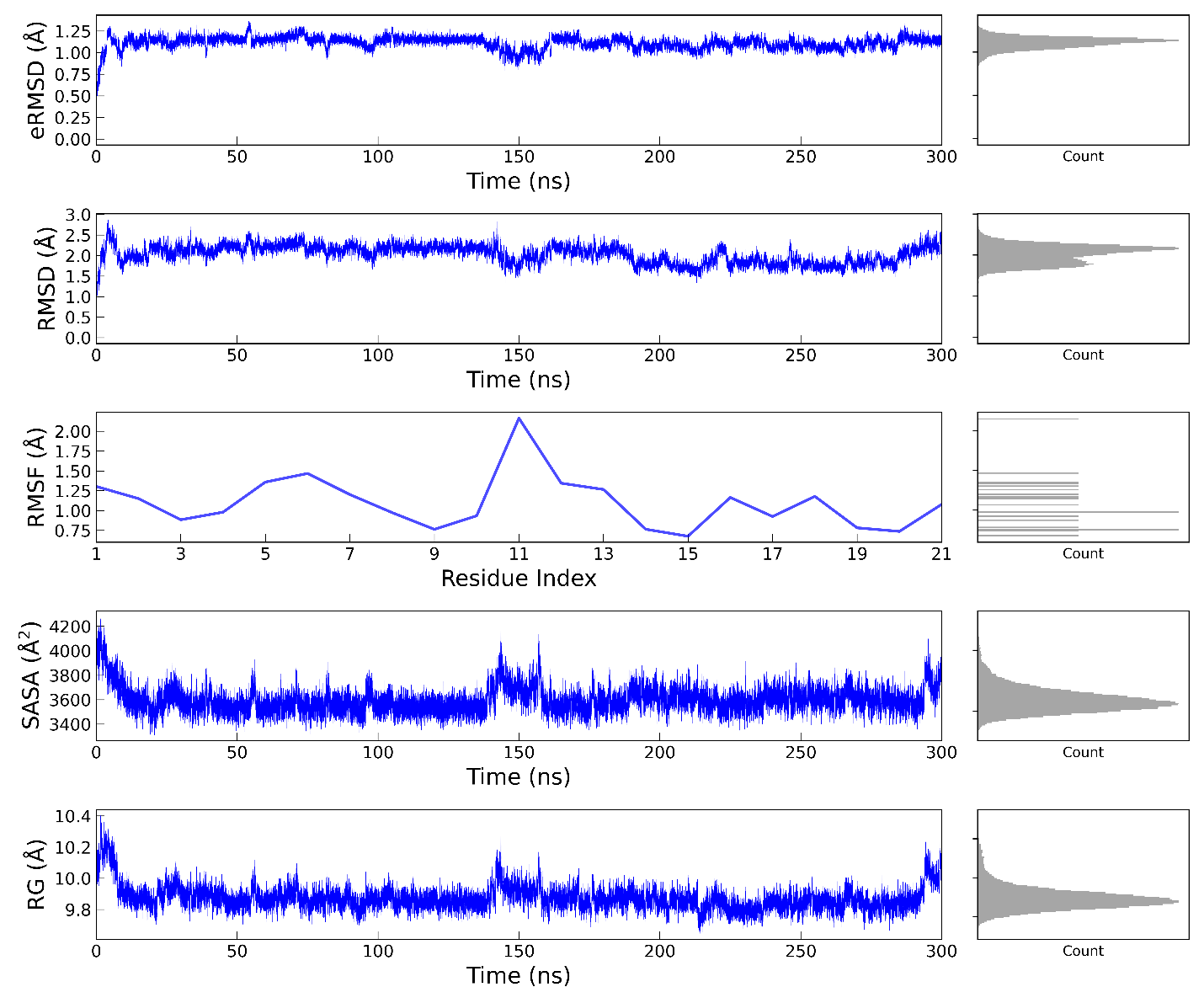
**Figure S8.1.** Global structural and dynamic features during a 300 ns standard MD simulation of 2MFU replica 2. (a) *eRMSD* and (b) *RMSD* of heavy atoms highlight structural stability, with eRMSD ranging from 0.75–1.25 Å and RMSD from 1.5–3.0 Å. (c) *RMSF* reveals flexibility at loop residues T11 up to 2.25 Å, while stem regions remain stable within 1.25 Å. (d) *Solvent-accessible surface area (SASA)* and (e) *Radius of gyration (Rg)* indicate consistent compactness throughout the trajectory, with values distributed within 3400–4200 Å² and 9.7–10.3 Å, respectively.

**
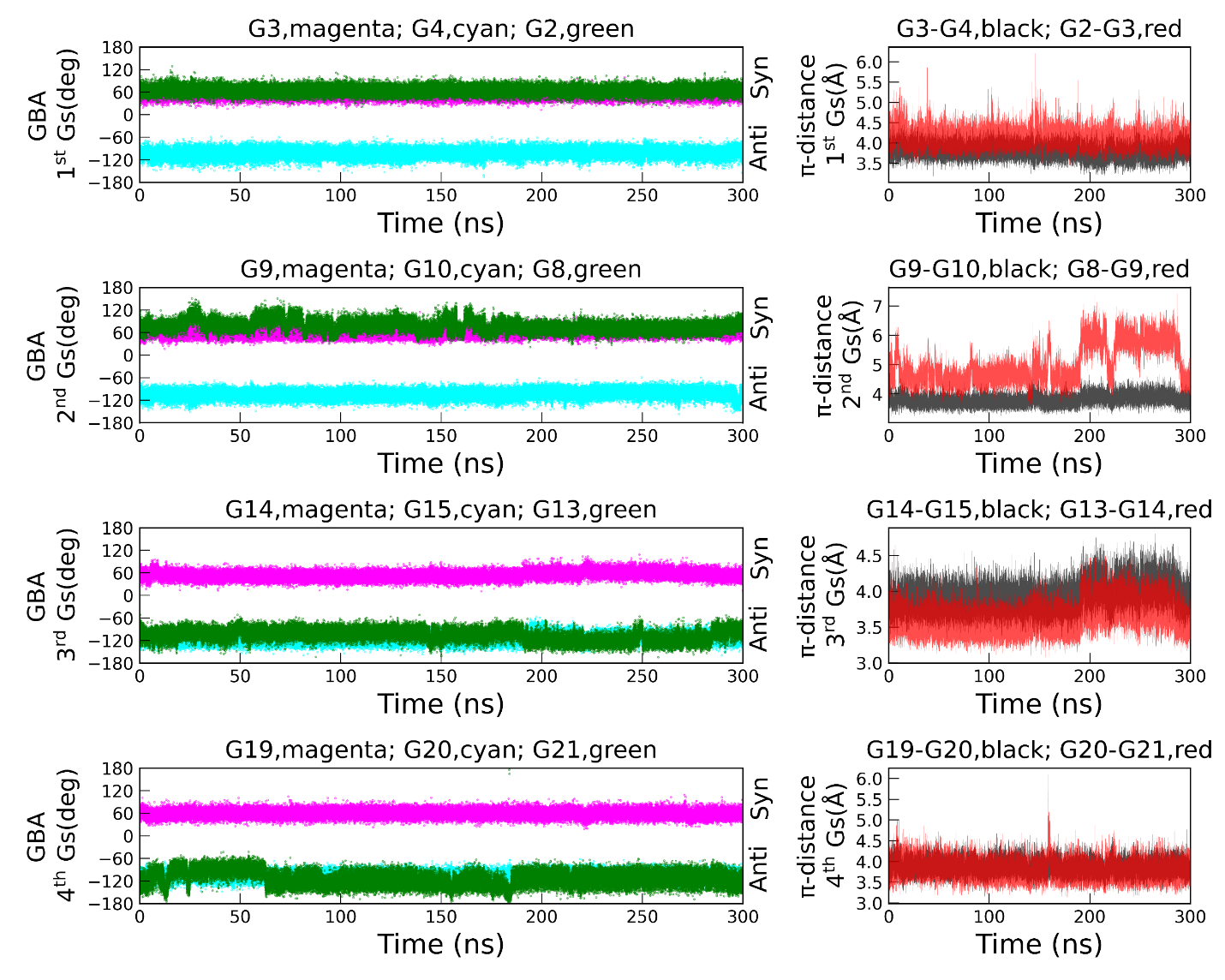
**

**Figure S8.2**. Glycosidic bond angles (GBA) and π-stacking interactions of three consecutive guanines in four G-strand during the 300 ns standard MD simulation of 2MFU replica 2. (a) Time evolution of GBA angles. The first G in the native G-strand adopts a *syn* conformation (+30~90°, magenta), while the second guanine adopts an *anti*-conformation (-90~-150°, cyan). The guanine outside the stem remains *syn* or *anti* (green). (b) π-stacking distances reveal the persistent stacking of native stem guanines (black) below 4.5 Å through the trajectory, and greater fluctuations of the non-stem guanines (red) up to 5.0 Å. Transient disruptions of G8-G9 π-stacking pair occasionally reaches 6.0 Å.

# VI. WT-Bias-Exchange Metadynamics (WT-BEMetaD)

**Strategy.** In replica bias exchange metadynamics^[28]^, unfolding was probed with three replicas (neutral, π-stack-biased, Hoogsteen-biased) exchanging every 30 ps by Metropolis criterion.

**Collective variables (CV).** We mapped inter-guanine distances to smooth contact variables using rational switching functions (m=6, n=12), placing the half-max at R_cut_ (5.2 Å for π-stacking; 5.0 Å for Hoogsteen)- see **Fig. S10, Table S3**. CV definitions are shown in **Fig. S9**, with atom/residue pairs in **Table S3.** Global CVs were defined as the sum of per-pair switches across the native stem to capture cooperative integrity; **Table S4**.

**Biasing protocol.** Gaussians: width σ = 0.05, initial filling rate W = 2.5 kJ·mol⁻¹·ps⁻¹, potential deposition τ_G = 1 ps⁻¹. Bias factors BF = 35, 40, 45, 55 were tested. A stepwise strategy was employed: first, biasing global CVs to probe overall stem stability; subsequently, applying bias only to strand-specific minimal CVs to enhance sampling of 3′ or 5′ triplex formations. All trials are summarized in **Table S4**.

**Sampling.** Each system ran >2 μs. Neutral replicas accumulated 522,826 frames/2.61 μs (3′ triplex) and 554,773 frames/2.77 μs (5′ triplex). BF optimization is shown in **Fig. S10**, where BF = 45 best sampled triplex/triad states while retaining the native G4.


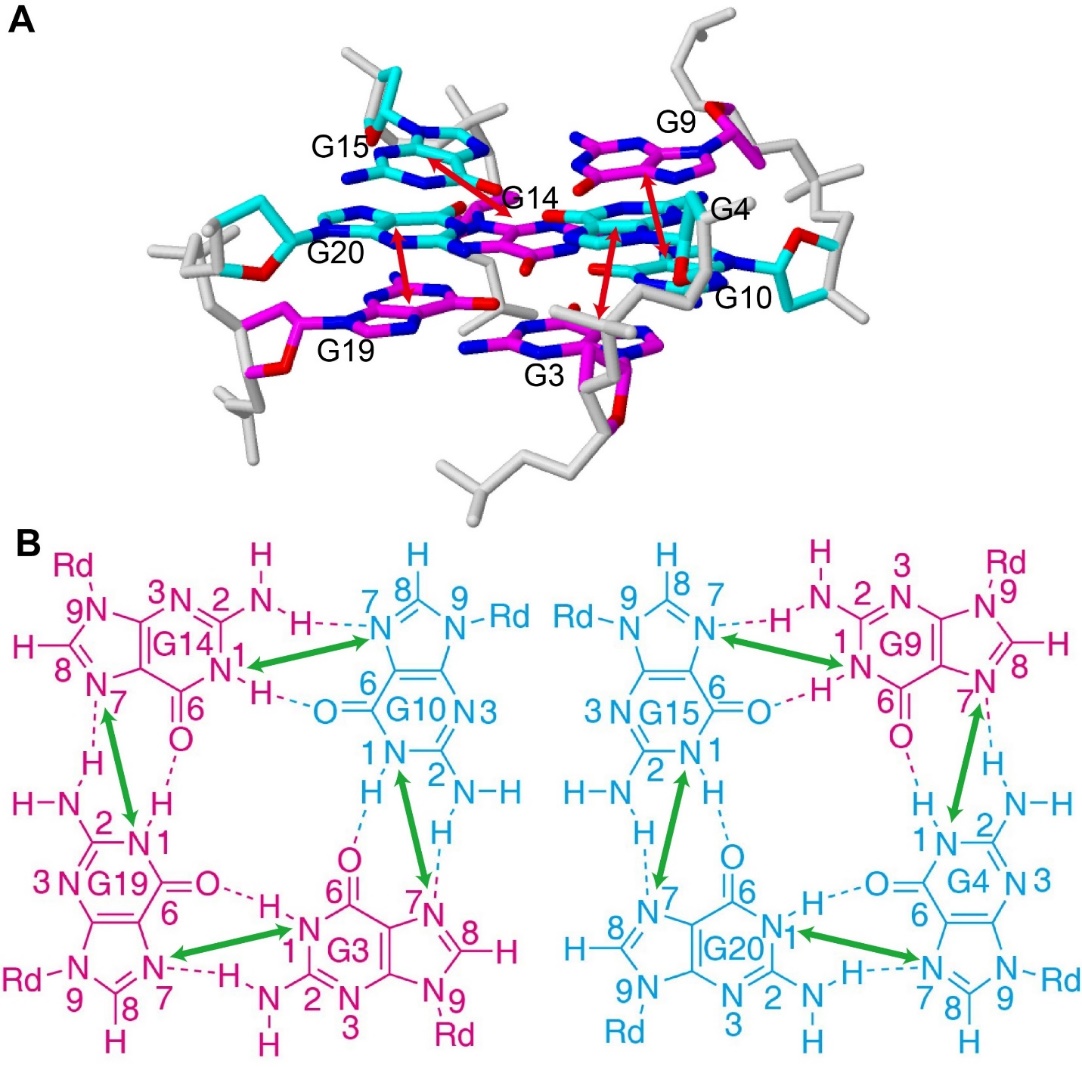


**Figure S9.** Schematic construction of collective variables (CVs) for π-stacking and Hoogsteen base pairing in the native G-stem topology. (A) π-stacking distances are defined as the center-of-mass distances between the C4–C5 bonds of consecutive guanines (red arrows). (B) Hoogsteen base pairs between adjacent guanines in each G-tetrad are simplified to a single heavy-atom distance (N7–N1), representing the combined contribution of two hydrogen bonds: N7–H21-N2 and O6–H1-N1 (green arrows). Guanosines in the *syn* conformation are colored magenta, while those in *anti* are colored cyan.

**Assessing unfolding of stem.** Several bias factors (BF = 35, 40, 45, and 55) were tested in order to identify the most appropriate bias strength for promoting partial unfolding of the native G-stem. Both collective variables (π–π stacking and Hoogsteen base pairing) were biased orthogonally with identical BF values. Each WT-BE-MetaD run consisted of three replicas (neutral, π-stack-biased, Hoogsteen-biased) exchanging every 30 ps under the Metropolis criterion. Gaussian deposition parameters were σ = 0.05, W = 2.5 kJ·mol⁻¹·ps⁻¹, τ_G = 1 ps⁻¹, with 1 ps hill deposition. Simulation lengths were between 2.1 and 2.3 µs for each BF value.

Two-dimensional free-energy surfaces reconstructed from neutral replicas at 300 K (400 × 400 grid) are shown in **Fig. S11A**. At lower bias strength (BF = 35) the trajectories remained largely confined to the G4 basin, whereas very high bias (BF = 55) resulted in excessive unfolding and loss of intermediate structures. Intermediate bias values (BF = 40 and 45) generated distinct and reproducible metastable basins corresponding to triad–tetrad (Tr–Te), triplex (Tp), Hoogsteen-pair (Hp), and unfolded (Unf) states. The corresponding normalized occupancy probabilities over time (**Fig. S11B**) indicate that, for BF = 45, populations were distributed among G4 (≈ 38.4 %), Tr–Te (≈ 39.4 %), Tp (≈ 6.9 %), and Unf (≈ 15.1 %), demonstrating balanced sampling of both native and intermediate basins.

Based on these results, BF = 45 was selected as the optimal bias factor for subsequent targeted WT-BE-MetaD simulations (5′-end- and 3′-end-biased protocols). A schematic overview of the exchange scheme and corresponding bias potential is provided in **Fig. S11C**.

**Table S3.** Definition of residue and atom pairs used for constructing global and minimal collective variables (CVs) in π-stacking and Hoogsteen base pairing. Residue pairs for π-stacking are defined between consecutive guanines in native G-stem, up to 4 pairs in global CV, and 1 pair in minimal CV. Atom pairs for Hoogsteen base pairs are based on N7–N1 interactions within each G-tetrad, up to 8 pairs in global CV, and 4 or 2 pairs in minimal CV.

| **Global CV**  **π-stacking** | | **Global CV**  **Hoogsteen base pair** | |
| --- | --- | --- | --- |
| **Scope** | **Pairs of residues** | **Scope** | **Pairs of residues** |
| ***Native G-stem*** | G3-G4 | ***Native G-stem*** | G3N7-G10N1 |
|  |  |  | G10N7-G14N1 |
|  | G9-G10 |  | G14N7-G19N1 |
|  |  |  | G19N7-G3N1 |
|  | G14-G15 |  | G4N7-G20N1 |
|  |  |  | G20N7-G15N1 |
|  | G19-G20 |  | G15N7-G9N1 |
|  |  |  | G9N7-G4N1 |
| **Minimal CV**  **π-stacking** | | **Minimal CV**  **Hoogsteen base pair** | |
| ***1^st^ G-strand*** | G3-G4 | ***1^st^ G-strand*** | G3N7-G10N1 |
|  |  |  | G19N7-G3N1 |
| ***G3 on 1^st^ G-strand*** | G3-G4 |  | G4N7-G20N1 |
|  |  |  | G9N7-G4N1 |
| ***4^th^ G-strand*** | G19-G20 | ***G3 on 1^st^ G-strand*** | G3N7-G10N1 |
|  |  |  | G19N7-G3N1 |
| ***G19 on 4^th^ G-strand*** | G19-G20 | ***4^th^ G-strand*** | G14N7-G19N1 |
|  |  |  | G19N7-G3N1 |
|  |  |  | G4N7-G20N1 |
|  |  |  | G20N7-G15N1 |
|  |  | ***G19 on 4^th^ G-strand*** | G14N7-G19N1 |
|  |  |  | G19N7-G3N1 |

**
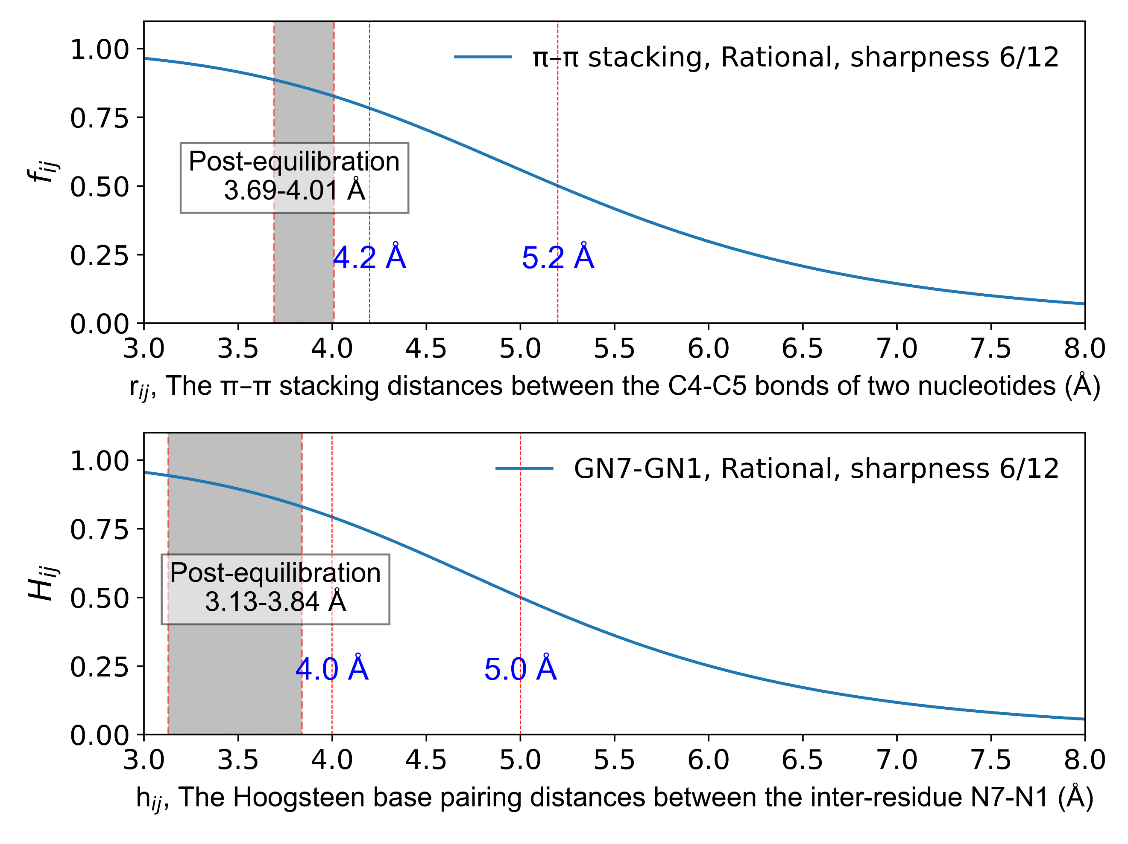
**

$$\boldsymbol{f}_{\boldsymbol{ij}}\boldsymbol{=}\frac{\boldsymbol{1-}\left( \frac{\boldsymbol{r}_{\boldsymbol{ij}}}{\boldsymbol{R}_{\boldsymbol{cut}}} \right)^{\boldsymbol{m}}}{\boldsymbol{1-}\left( \frac{\boldsymbol{r}_{\boldsymbol{ij}}}{\boldsymbol{R}_{\boldsymbol{cut}}} \right)^{\boldsymbol{n}}}$$

$$\boldsymbol{H}_{\boldsymbol{ij}}\boldsymbol{=}\frac{\boldsymbol{1-}\left( \frac{\boldsymbol{h}_{\boldsymbol{ij}}}{\boldsymbol{R}_{\boldsymbol{cut}}} \right)^{\boldsymbol{m}}}{\boldsymbol{1-}\left( \frac{\boldsymbol{h}_{\boldsymbol{ij}}}{\boldsymbol{R}_{\boldsymbol{cut}}} \right)^{\boldsymbol{n}}}$$

**Figure S10**. Rational Switching Functions Defining CVs for WT-Bias-Exchange MetaD of 2MFU (π-Stacking and Hoogsteen Contacts). Distance-based rational switching functions convert inter-guanine distances into smooth [0–1] CV contributions used in WT-BE-MetaD. Post-equilibration MD gave the native distance bands (π–π C4–C5: 3.69–4.01 Å; Hoogsteen N7–N1: 3.13–3.84 Å; gray). Cutoffs were set at the half-max points (R_cut,π_ = 5.2 Å; R_cut,Hg_ = 5.0 Å) with sharpness m = 6, n = 12 to yield gradual decay outside native contact ranges. Global CVs are simple sums over per-pair switches (cooperative coordination); minimal CVs restrict to strand-specific pairs for targeted bias replicas.

## **Table S4.** WT-BEMetaD simulations of all stepwise attempts, differing in bias factors of the global collective variables, and the scopes of the minimal collective variables.

| **Study** | **Scopes of Collective Variables in biased replica** | **Range of biased** **π-stacking CV** | **Range of biased Hoogsteen base pair CV** | **Bias factor** | **Simulation length (ns)** |
| --- | --- | --- | --- | --- | --- |
| 1 | Native G-stem | 0-4 | 0-8 | 35 | 2180.54 |
| 2 | Native G-stem | 0-4 | 0-8 | 40 | 2148.905 |
| 3 | Native G-stem | 0-4 | 0-8 | 45 | 2304.095 |
| 4 | Native G-stem | 0-4 | 0-8 | 55 | 2138.975 |
| 5 | 1^st^ G-strand G3-G4 | 0-1 | 0-4 | 45 | 2614.13 |
| 6 | G3 of 1^st^ G-strand | 0-1 | 0-2 | 45 | 2696.91 |
| 7 | 4^th^ G-strand G19-G20 | 0-1 | 0-4 | 45 | 2773.865 |
| 8 | G19 of 4^th^ G-strand | 0-1 | 0-2 | 45 | 2719.29 |


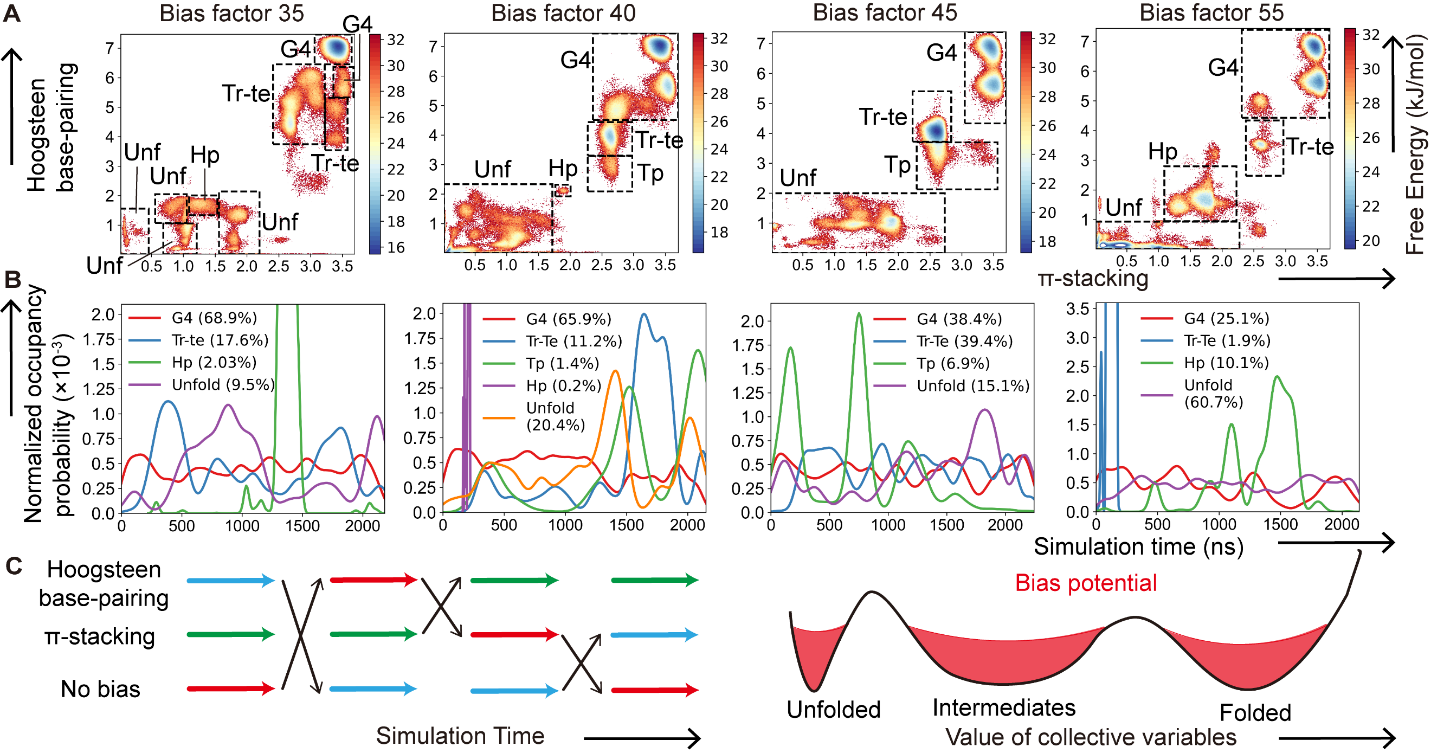


**Figure S11.** Bias-factor optimization in WT-Bias-Exchange MetaD for unfolding of the native G-stem of 2MFU. (A) Two-dimensional free-energy surfaces (FES) obtained from neutral replicas of WT-BE-MetaD simulations in which both collective variables- π-stacking (x-axis) and Hoogsteen base-pairing (y-axis)- were biased orthogonally with identical bias factors (BF = 35, 40, 45, 55). Each FES was reconstructed at 300 K on a 400 × 400 grid using a 2D reweighted histogram. Gaussian parameters: σ = 0.05 (distance), W = 2.5 kJ·mol⁻¹·ps⁻¹, τ_G = 1 ps⁻¹; bias deposition every 1 ps; replica exchanges every 30 ps by the Metropolis criterion. The color scale represents free energy (kJ·mol⁻¹). Dashed boxes delineate metastable basins: G4 (native), Tr–Te (triad–tetrad), Tp (triplex), Hp (Hoogsteen pair), and Unf (unfolded). At low bias (BF = 35), sampling remains confined to the G4 basin; at very high bias (BF = 55), unfolding dominates with loss of structured intermediates. Intermediate bias levels (BF = 40–45) yield clear Tr–Te and Tp basins. (B) Normalized occupancy probabilities of individual basins as a function of simulation time (≈ 2.1–2.3 μs for each BF). Populations were calculated from centroid clustering in the 2D CV plane and expressed as fractions of total frames. For BF = 35 and 40, the G4 basin remains predominant (≈ 66–69%), while for BF = 55 the unfolded basin dominates (≈ 61%). In contrast, BF = 45 produces balanced sampling between native (38.4%), Tr–Te (39.4%), and triplex (Tp, 6.9%) species, with minimal oversampling of completely unfolded conformers (15.1%). This level of bias promotes sufficient disruption of the G-stem to access triplex intermediates without erasing the folded state. (C) Schematic representation of the WT-BE-MetaD exchange protocol and bias potential. Three replicas- π-stacking-biased, Hoogsteen-biased, and neutral- exchange every 30 ps following the Metropolis acceptance rule. Gaussian hills deposited along each collective variable lower free-energy barriers, promoting transitions between basins. The schematic free-energy profile illustrates how the bias potential (red) facilitates movement between the folded (G4), intermediate (Tr–Te/Tp), and unfolded (Unf) states. The bias-factor analysis identifies BF = 45 as the most suitable value for probing partial unfolding of the stem, since it yields significant residency of a triplex-like species consistent with the experimentally observed intermediate-associated basin from SRCD thermodynamics and to triplex minima recovered in DFT optimization. This bias factor was therefore used for all subsequent targeted WT-BE-MetaD simulations of 5′- and 3′-end-directed unfolding.

# VII. Free-energy reconstruction and convergence

**2D free-energy surfaces (FES).** 2D FES from neutral replicas were obtained via a 2D reweighted-histogram at 300 K on a 400 × 400 CV grid ($F = -k_{B}T\ln P(CV1,CV2)$). Basins were assigned by nearest-centroid regions in the 2D CV plane; occupancies and time traces were computed from the neutral-replica trajectory. Basins for G4, and unfolding intermediate states using 5′-end bias (**Figure 5, Fig. S15**), and 3’-end bias used BF=45 (**Fig. S12**).

**1D projections and convergence.** To assess stability, FES were projected onto single CVs in cumulative blocks (0.5→2.6 μs). Overlapping profiles confirmed convergence of basin positions and barrier heights for both 3′ and 5′ triplexes (**Fig. S13**).

**Basin statistics.** Populations, mean energies, and minima are summarized in **Fig. S14**. Energies are in kJ·mol⁻¹ (global min = 0). When biasing underestimated native/non-native ΔG, basin weights were corrected using BF = 45 standard populations for cross-FES comparison.

**CD-Anchored Rescaling of Free-Energy Surfaces at 25 °C**

Neutral-replica, free energy of 2D FES $G_{i}^{\mathrm{MD}}$​were partitioned into three disjoint sets: $G_{F}^{*}$, the native basin minimum; $\left\langle G \right\rangle_{I}^{*}$and $\left\langle G \right\rangle_{U}^{*}$, population-weighted mean over I (Intermediate, the triplex basins) and U (Unfolded, the rest basins). Separate affine maps $G_{i}^{\text{*}}=a G_{i}^{\text{MD}}+b$ were applied to the 5′-end and 3′-end FES using 2D-reweighted histogram energies (no additional correction). The CD-derived free energy was anchored at disjoint sets: Native (F) fixed at the native basin minimum $G_{F}^{*}$= ${\Delta G}_{F \mathrm{vs} U}^{\mathrm{CD}}$​ = -7.49 kJ/mol; Intermediate (I) defined as ‘pooled triplex’ ensembles (Tp) (In 5′-end: 5′-III, 5′-V, 5′-VI; In 3′-end: 3′-II, 3′-III) fixed at$\left\langle G \right\rangle_{I}^{*}$​= ${\Delta G}_{I \mathrm{vs}U}^{\mathrm{CD}}$​= -3.11 kJ/mol. Unfolded (U) is the reference $\left\langle G \right\rangle_{U}^{*}$= ${\Delta G}_{\mathrm{vs} U}^{\mathrm{CD}}$​= ${\Delta G}_{unfolding}$= 0.

In the case of anchoring the intermediate level $\left\langle G \right\rangle_{I}^{*}$to ${\Delta G}_{I \mathrm{vs}U}^{\mathrm{CD}}$, this yields $a=\frac{-3.11-0}{\left\langle G \right\rangle_{I}^{MD}- \left\langle G \right\rangle_{U}^{MD}},b=-a \left\langle G \right\rangle_{U}^{\text{MD}}$. The resulting coefficients were a = 1.096895; b = -28.276167 for the 5′-end and a = -1.572868; b = 27.962441 for the 3′-end bias. The rescaling fixed the zero at U, matches the experimental spacing between F and I, and preserves the triplex ensembles in MD (**Table S5-S6**). Under the same anchoring (F = −7.49 kJ mol⁻¹; pooled triplex I = −3.11 kJ mol⁻¹ at 25 °C), the 5′‑end biasing series yields a positive slope (a = +1.0969; b = −28.2762) that preserves the expected F < I < U stability ordering (ΔG_vsU), whereas the 3′‑end series requires a negative slope (a = −1.572868; b = 27.962441) that inverts relative energies; for this reason we interpret the G-triplex resultant from 5′-end biasing as the experimental intermediate.

**Tables S5–S6 (rescaled energies, BF=45).** 5′-end–biased FES (I = pooled {5′-III, 5′-V, 5′-VI}) and 3′-end–biased FES (I = pooled {3′-II, 3′-III}) are summarized with populations (%), raw minima (kJ mol⁻¹), and rescaled $\Delta G$values versus U (kJ mol⁻¹). Consider that:
(i) Reweighted histogram uses the neutral-replica bias for reweighting only; no additional post-hoc correction was applied.
(ii) Basin centroids were fixed from the full neutral trajectory; assignments per time-slice use nearest-centroid.
(iii) CD anchors and their 95% CIs come from wavelength bootstrap (B=300) under the three-state global fit (Section IV).
(iv) For cross-FES comparisons where the native/non-native spacing is underestimated, we reference BF = 45 populations.

For the 3′ series, the fitted slope is negative ($a<0$) because the raw neutral-replica FES places the pooled triplex mean below the unfolded mean; this preserves the anchored F–I spacing but can render some non-native basins numerically more stable than F. We therefore report both the two-anchor values (**Tables S5–S6**) and the monotone U–F map above.

Below, the SRCD‑anchored two‑anchor fit produces a monotone map for the 5′ series (**Table S5**), but a negative‑slope map for the 3′ series (**Table S6**), which can place some non‑native basins numerically below F; accordingly, we assign the experimental intermediate to the 5′‑strand‑directed triplex ensemble.

## **Table S5**. Re-scaled energies 5′-end–biased FES (BF 45) (I = pooled {5′‑III, 5′‑V, 5′‑VI}).

| **Species** | **Class** | **Topology** | **Pop. (%)** | **Raw (kJ·mol⁻¹)** | **ΔG_vsU (kJ·mol⁻¹)** | **ΔG_unfolding (kJ·mol⁻¹)** |
| --- | --- | --- | --- | --- | --- | --- |
| 5′-Nat | F | G4 | 47.0 | 18.95 | -7.49 | 7.49 |
| 5′-I | I | G4 | 4.8 | 24.62 | -1.27 | 1.27 |
| 5′-II | I | G4 | 10.1 | 22.71 | -3.37 | 3.37 |
| 5′-III | I | Tp | 8.8 | 22.94 | -3.11 | 3.11 |
| 5′-IV | I | Te-Hp | 7.4 | 24.56 | -1.34 | 1.34 |
| 5′-V | I | Tp | 2.7 | 25.93 | 0.17 | -0.17 |
| 5′-VI | I | Tp | 8.9 | 22.04 | -4.10 | 4.10 |
| 5′-VII | I | Te-Tr | 2.9 | 25.63 | -0.16 | 0.16 |

**Table S6**. Re-scaled energies 3′-end–biased FES (BF 45) (I = pooled {3′‑II, 3′‑III}). Classed ‘G4’ by centroid in CV plane; geometry shows partial stem rearrangement. Lower mapped energy than F arises from two-anchor rescaling with $a<0$

| **Species** | **Class** | **Topology** | **Pop. (%)** | **Raw (kJ·mol⁻¹)** | **ΔG_vsU (kJ·mol⁻¹)** | **ΔG_unfolding (kJ·mol⁻¹)** |
| --- | --- | --- | --- | --- | --- | --- |
| 3′-Nat | F | G4 | 7.4 | 22.54 | -7.49 | 7.49 |
| 3′-I | I | G4 | 1.3 | 26.24 | -13.31 | 13.31 |
| 3′-II | I | Tp | 14.3 | 21.29 | -5.52 | 5.52 |
| 3′-III | I | Tp | 32.5 | 19.08 | -2.05 | 2.05 |
| 3′-IV | I | G4 | 30.5 | 18.25 | -0.74 | 0.74 |
| 3′-V | I | G4 | 2.4 | 25.06 | -11.45 | 11.45 |

Notes. (i) 2D-reweighted histogram outputs were used directly; no extra 'corrected' energies were applied. (ii) Tp ensemble anchoring ensures the CD-observed S2 lies between F and U; any Tp microstate slightly above U reflects a marginal/partially formed triplex.


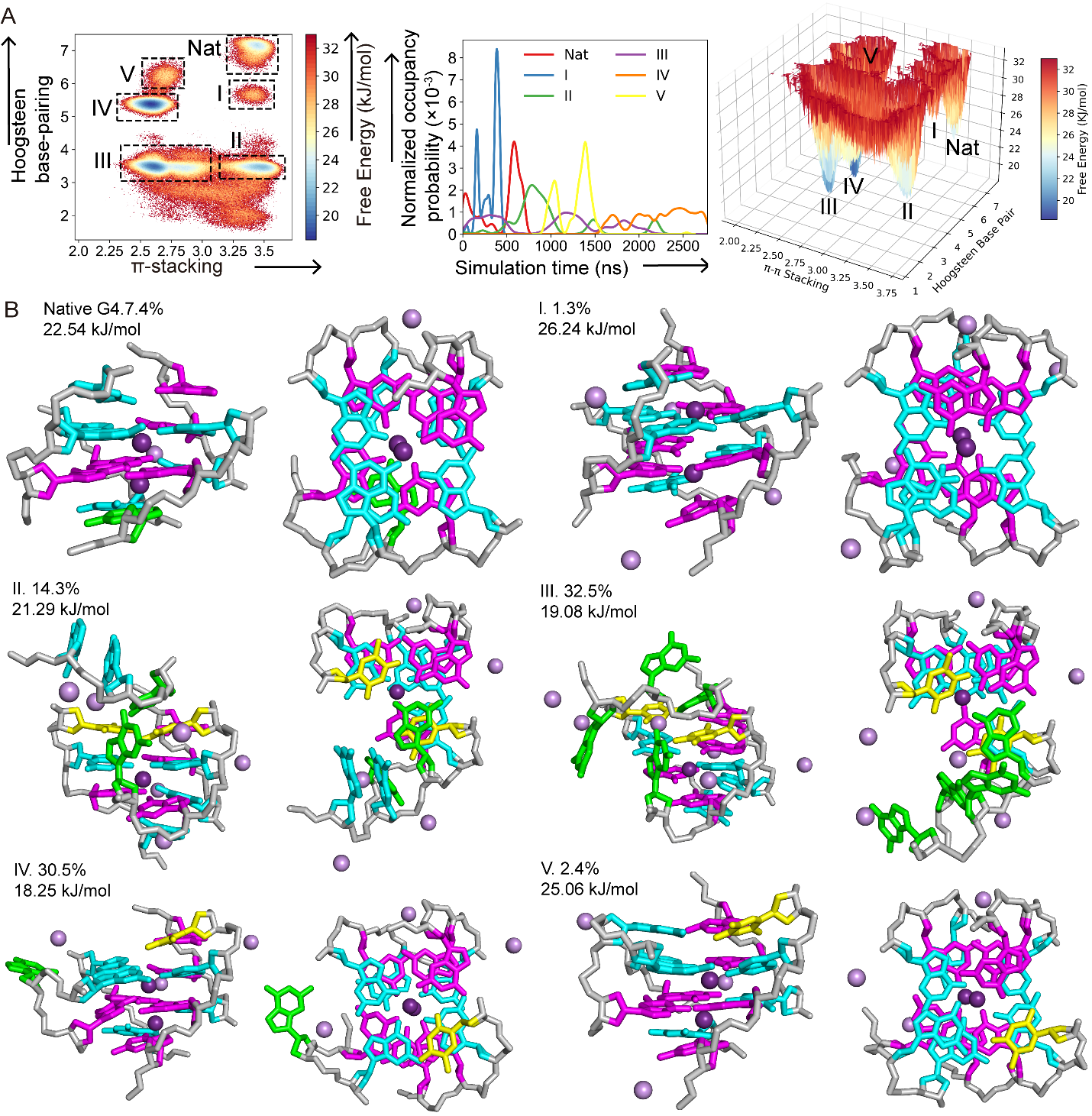


**Figure S12**. 3′-end-directed unfolding of 2MFU at optimal bias factor (BF = 45) in WT-Bias-Exchange MetaD. (A) Two-dimensional free-energy surface (FES) reconstructed from the neutral replica of a WT-BE-MetaD simulation in which both collective variables, π–π stacking (x-axis) and Hoogsteen base-pairing (y-axis), were biased orthogonally with identical bias factor BF = 45. Gaussian deposition parameters: σ = 0.05 Å, W = 2.5 kJ·mol⁻¹·ps⁻¹, τ_G = 1 ps⁻¹, hill deposition every 1 ps; replica exchanges occurred every 30 ps by the Metropolis criterion. The FES (400 × 400 grid, 300 K) shows basins corresponding to the native quadruplex (Nat) and five metastable intermediates (I–V). Free-energy minima (kJ·mol⁻¹, color-coded) are labeled according to centroid clustering in the two-dimensional CV space. The middle panels show the temporal evolution of basin occupancies and the 3-D representation of the FES, confirming the stability of a triplex-like intermediate (basin III). Populations were normalized over 554,773 frames (2.77 µs total simulation). (B) Representative structures of the main basins obtained from clustering of neutral-replica frames. Populations and mean free energies (kJ·mol⁻¹) are reported for each basin. The following is observed: (i) Native G4 (7.4 %, 22.54 kJ·mol⁻¹): intact 4-tetrad stem with canonical Hoogsteen geometry; (ii) I (1.3 %, 26.24 kJ·mol⁻¹): partially disrupted 3′ triad with frayed top tetrad; (iii) II (14.3 %, 21.29 kJ·mol⁻¹): triplex precursor with one guanine partially detached from the top layer; (iv) III (32.5 %, 19.08 kJ·mol⁻¹): dominant triplex-like basin (Tp) showing a stable 3-stack arrangement capped by loop thymines; a structurally plausible triplex state, but not the experimentally assigned counterpart used for the final CD-anchored interpretation; (v) IV (30.5 %, 18.25 kJ·mol⁻¹): triad–tetrad exchange (Te/Tr) with lateral loop rearrangement; (vi) V (2.4 %, 25.06 kJ·mol⁻¹): partially unfolded conformation retaining a Hoogsteen triad. Colour scheme: *syn* guanines = magenta, *anti* guanines = cyan, loop thymines = yellow, extrastem guanines = green, central sodium = deep purple, groove sodium = light purple.


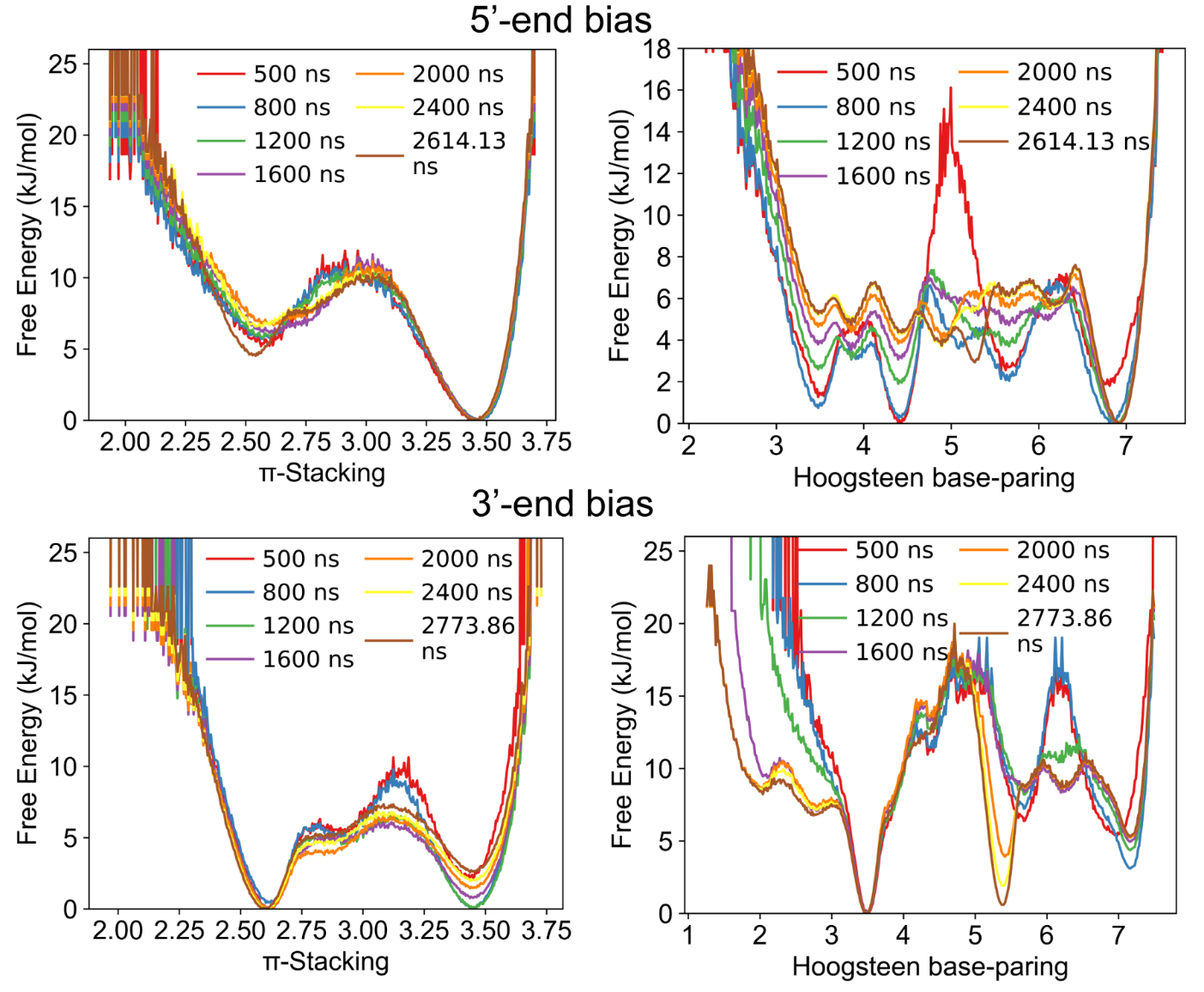


**Figure S13**. The 1D projections and convergence of WT-BEMetaD strategies on the neutral replicas of minimal CV for exploring 5′-end and 3′-end bias mechanisms (bias factor 45). Simulation times: 2614.13 ns (5′-end bias) or 2773.865 ns (3′-end bias). X-axis: π-stacking, or Hoogsteen base-pairing. Y-axis: Free energy, units in kJ/mol.


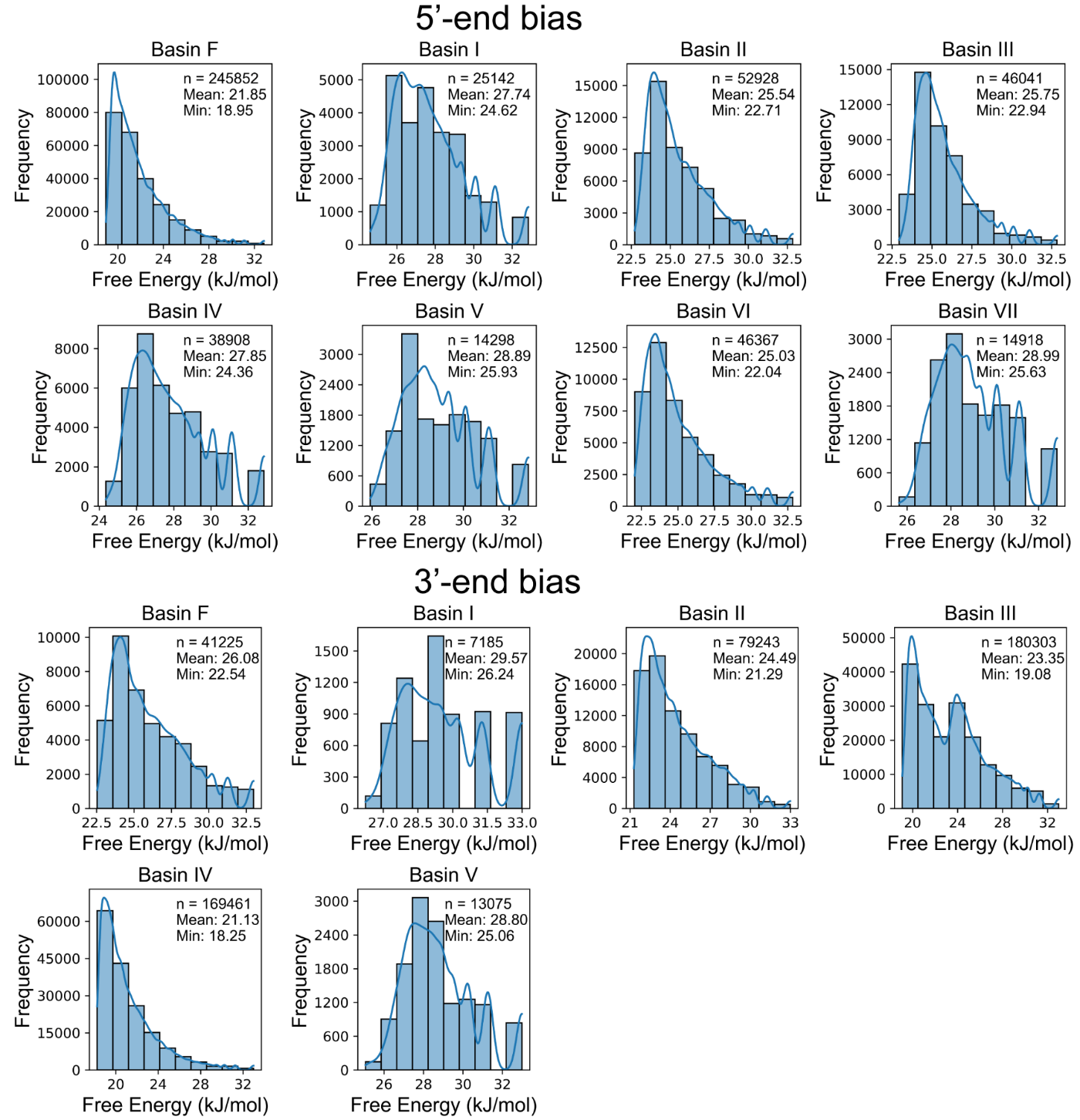


**Figure S14**. The free energy statistics of WT-BEMetaD strategies in each basin of minimal CV for exploring 5′-end and 3′-end bias mechanisms (Bias factor 45). Total population (n) in neutral replica: 522826 (2614.13 ns, 5′-end bias), or 554773 (2773.865 ns, 3′-end bias).


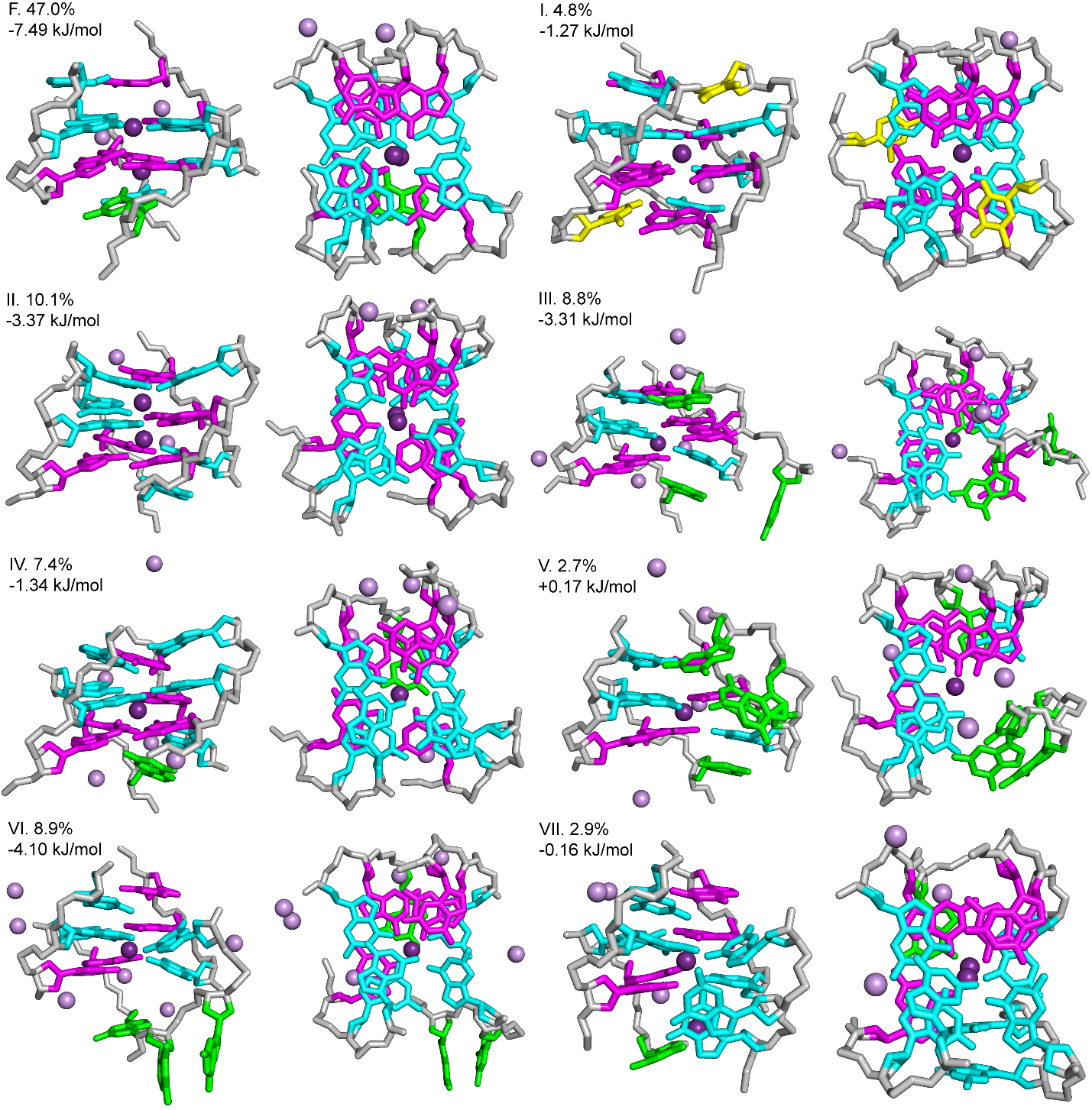


**Figure S15**. Structures of step-wised unfolding mechanism of 5′-end bias. Structural views of metastable states are shown, with bias CV applied to the first G-strand (G3-G4) and simulated for 2614.13 ns, detecting 7 non-native basins. Color scheme: *Syn*, magenta; *Anti*, cyan; Thymine, yellow; guanosine outside G-stem, green; central sodium, deep purple; groove sodium, light purple.

# VIII. TD-DFT/ECD calculations

## **1. 2MFU model preparation and TD‑DFT calculations**

**NMR-restrained refinement.** To prepare solution-like starting structures, NMR-restrained MD refinements were performed with empirical distance/planarity restraints (10 Å solvent box; 100 mM NaCl). Statistics are shown in **Table S7**.

**QM levels and models**. Electronic structure calculations were carried out with ORCA 5.0.4^[29, 30]^ at the CAM-B3LYP^[31]^/def2-TZVP^[32]^ level, including CPCM implicit solvent (water)^[33]^ and D3BJ dispersion corrections with RIJCOSX^[34]^ acceleration. Models consisted of truncated stems of 8 or 11 guanosine bases (atom H replace C1'), each with three channel ions (Na⁺ or K⁺). Hydrogen-bond constraints are listed in **Table S8**.

**Excitations**. 300 singlet excited states (TDA^[35]^) were computed per model. Each excitation yields a wavelength λᵢ (nm) and a rotatory strength $R_{i}$ (in 10⁻⁴⁰ esu²·cm²).

**Adaptive convolution of TD-DFT spectra**. To convert discrete TD-DFT^[36]^ excitations into continuous spectra directly comparable with experimental CD, we applied a four-step protocol.
1. Gaussian line shape
Each excitation is represented by a normalized Gaussian line shape:

$g_{i}\left( \lambda_{i} \right)=\frac{1}{\sigma_{i}\sqrt{2\pi}}\exp\left( -\frac{\left( \lambda-\lambda_{i} \right)^{2}}{2\sigma_{i}^{2}} \right)$ (**Eq. 1**)
where λ is wavelength (nm) and σᵢ is the wavelength-dependent broadening.

2. Dynamic Gaussian broadening
To account for spectral congestion at longer wavelengths, $\sigma_{i}$ varies linearly across the spectral domain:

$\sigma_{i}=\sigma_{\text{m}\text{in}}+\left( \frac{\lambda_{i}-\lambda_{\text{min}}}{\lambda_{\text{max}}-\lambda_{\text{min}}} \right)\left( \sigma_{\text{max}}-\sigma_{\text{min}} \right)$ (**Eq. 2**)
where *λ_min_* and *λ_max_* are the minimum and maximum excitation wavelengths in the raw TD-DFT output, *σ_min_* = 3.5 nm and *σ_max_* = 5.5 nm.

3. Wavelength alignment
To place the computed spectrum in the experimental window, the discrete excitations were affinely transformed:

${\lambda'}_{i}=s\cdot\lambda_{i}+\Delta$  **(Eq. 3)**
Parameters *s* and Δ were chosen so that ${\lambda'}_{\text{max}}$ = 350 nm and ${\lambda'}_{\text{m}\text{in}}$ = 205 nm.

4. Unit conversion
The molar circular dichroism $\Delta\varepsilon\left( \lambda\right)$ in M⁻¹·cm⁻¹ is obtained as:

$$\Delta\varepsilon\left( \lambda\right)=\left( \pi/\left( Nₐhcln\left( 10 \right) \right) \right)\sum Rᵢgᵢ\left( \lambda^{'} \right) (\mathbf{Eq. 4})$$

where Nₐ is Avogadro’s constant, h Planck’s constant, and c the speed of light. The factor of π normalizes Gaussian vs Lorentzian conventions, following Stephens–Devlin–Frisch.^[37]^ The empirical formula is physically equivalent for deriving optical rotatory dispersion, with an normalized π factor:^[38, 39]^

$\Delta\varepsilon\left( \lambda_{i} \right)=Rᵢgᵢ\left( \lambda_{i} \right)\cdot\frac{\lambda_{i}}{22.94\cdot\sqrt{\pi}\cdot\sigma_{i}}\cdot{10}^{40}\cdot\pi$ $(\mathbf{Eq. 5})$

**Final spectrum.** The continuous broadened spectrum is:
$S\left( \lambda\right)=$ $\sum{\Delta\varepsilon}_{i}\left( \lambda\right)$ (**Eq. 6**)
Spectra were computed on a uniform grid (Δ*λ* = 1 nm) spanning 205–350 nm.

**Spectral overlays.** Final broadened spectra **(Eq. 6)** were compared with experimental SRCD spectra in the 205–350 nm range **(Figs. S16–S18).** Excitation wavelengths for overlay are listed in **Table S9**.

**Implementation notes**
- Software: Python 3.11 (NumPy, SciPy).^[40]^
- Interpolation: Excitations broadened directly onto the uniform grid.
- Truncation: Gaussian tails included across the full domain; outside 205–350 nm spectra were clipped.

All scripts and input files (Python 3.11; NumPy/SciPy; ORCA input sets; plotting notebooks) are available at our public repository. Trajectories of WT-BEMetaD simulation are exclusive in Zenodo.

Zenodo: <https://doi.org/10.5281/zenodo.15832342>

GitHub: <https://github.com/hlyqian-y1/Project_MD-DFT_2MFU-2MKM_Github>

**Table S7**. Pre-DFT optimization on 2MFU G-quadruplex DNA with empirical distance restraints in Amber MD simulation. The π-stacking distances between the C4-C5 bonds of two nucleotides are independently described as the distance between inter-guanosine C4-C4 and C5-C5 in the distance restraint, featured with Syn-Anti as the GBA step. The Hoogsteen base pairs of coplanar inter-residues are described as the distances between the separated N7-H21, N7-N2, O6-H1, and O6-N1. The lower and outer boundaries are allowed with 0.15 Å. In the guanosines outside the 2-tetreds G-stem, G8, G13, and G21 lower and outer boundaries are stacked and are allowed with 0.25 Å while G2 was detached.

| **Residues(2MFU)** | **Empirical Restraints** | | **Refined Structures** | | |
| --- | --- | --- | --- | --- | --- |
| **π-stacking Between Guanosines** | C4-C4(Å) | C5-C5(Å) | | C4-C4(Å) | C5-C5(Å) |
| G3(C)-G4(C), **Syn-Anti** | 4.21 ± 0.15 | 3.38 ± 0.15 | | 4.1401 | 3.4706 |
| G8(C)-G9(C), **Syn-Syn** | **4.42 ± 0.25** | **3.99 ± 0.25** | | **4.9411** | **4.6425** |
| G9(C)-G10(C), **Syn-Anti** | 4.21 ± 0.15 | 3.38 ± 0.15 | | 4.2829 | 3.5108 |
| G13(C)-G14(C), **Anti-Syn** | **4.45 ± 0.25** | **4.96 ± 0.25** | | **4.9513** | **5.5932** |
| G14(C)-G15(C), **Syn-Anti** | 4.21 ± 0.15 | 3.38 ± 0.15 | | 4.5599 | 3.5414 |
| G19(C)-G20(C), **Syn-Anti** | 4.21 ± 0.15 | 3.38 ± 0.15 | | 4.3695 | 3.6529 |
| G20(C)-G21(C), **Anti-Anti** | **4.08 ± 0.25** | **3.75 ± 0.25** | | **4.0837** | **3.8919** |
| **Hydrogen bond within G-stem** | N7/O6-H21/H1(Å) | N7/O6-N2/N1(Å) | | N7/O6-H21/H1(Å) | N7/O6-N2/N1(Å) |
| **G3:G10:G14:G19 tetrad** |  |  | |  |  |
| G3(N-7)-G10(H-21) | 1.99 ± 0.15 | 2.9 ± 0.15 | | 1.9312 | 2.9006 |
| G3(O-6)-G10(H-1) | 1.95 ± 0.15 | 2.95 ± 0.15 | | 1.7638 | 2.7638 |
| G10(N-7)-G14(H-21) | 1.99 ± 0.15 | 2.9 ± 0.15 | | 1.8689 | 2.8756 |
| G10(O-6)-G14(H-1) | 1.95 ± 0.15 | 2.95 ± 0.15 | | 1.8449 | 2.8100 |
| G14(N-7)-G19(H-21) | 1.99 ± 0.15 | 2.9 ± 0.15 | | 1.9548 | 2.9212 |
| G14(O-6)-G19(H-1) | 1.95 ± 0.15 | 2.95 ± 0.15 | | 1.9707 | 2.9701 |
| G19(N-7)-G3(H-21) | 1.99 ± 0.15 | 2.9 ± 0.15 | | 1.9002 | 2.8490 |
| G19(O-6)-G3(H-1) | 1.95 ± 0.15 | 2.95 ± 0.15 | | 1.7964 | 2.8061 |
| **G4:G20:G15:G9 tetrad** |  |  | |  |  |
| G4(N-7)-G20(H-21) | 1.99 ± 0.15 | 2.9 ± 0.15 | | 1.9803 | 2.9527 |
| G4(O-6)-G20(H-1) | 1.95 ± 0.15 | 2.95 ± 0.15 | | 1.8246 | 2.8337 |
| G20(N-7)-G15(H-21) | 1.99 ± 0.15 | 2.9 ± 0.15 | | 1.9462 | 2.9300 |
| G20(O-6)-G15(H-1) | 1.95 ± 0.15 | 2.95 ± 0.15 | | 1.9049 | 2.8096 |
| G15(N-7)-G9(H-21) | 1.99 ± 0.15 | 2.9 ± 0.15 | | 1.8394 | 2.8468 |
| G15(O-6)-G9(H-1) | 1.95 ± 0.15 | 2.95 ± 0.15 | | 2.0841 | 3.0735 |
| G9(N-7)-G4(H-21) | 1.99 ± 0.15 | 2.9 ± 0.15 | | 2.0070 | 2.9888 |
| G9(O-6)-G4(H-1) | 1.95 ± 0.15 | 2.95 ± 0.15 | | 1.8162 | 2.8217 |

**Table S8**. Constrained hydrogen-bond distances used in DFT geometry optimization of 2MFU G-quadruplex DNA structures. Distance constraints were applied to coplanar guanine residues in 4 DFT systems of 2MFU (11 guanosines with 3 K^+^; 8 guanosines with 3 K^+^; 11 guanosines with 3 Na^+^; 8 guanosines with 3 Na^+^) using the ORCA quantum chemistry package. Hoogsteen hydrogen bonds were defined via fixed inter-residue distances (N7–H21, N7–N2, O6–H1, O6–N1), consistent with values from post-equilibrated MD structures. Target distances were adopted from X-ray data in study^[41]^: N7–H21, 1.99 ± 0.15 Å; N7–N2, 2.90 ± 0.15 Å; O6–H1, 1.95 ± 0.15 Å; O6–N1, 2.95 ± 0.15 Å. Distances exceeding boundaries were re-constrained to the nearest limit (values in **bold** and *parentheses*). Planarity and π-stacking between guanines were unconstrained to allow geometric relaxation during optimization.

| **Residues(2MFU)** | **2MFU initial structure** | |
| --- | --- | --- |
| **Hydrogen bond within G-tetrads** |  |  |
| **G3:G10:G14:G19 tetrad** | N7/O6-H21/H1(Å) | N7/O6-N2/N1(Å) |
| G3(N-7)-G10(H-21) | 1.9877 | 2.9840 |
| G3(O-6)-G10(H-1) | **2.2221 (2.10)** | **3.1332 (3.10)** |
| G10(N-7)-G14(H-21) | 1.9825 | 2.9613 |
| G10(O-6)-G14(H-1) | **2.2392 (2.10)** | **3.1310 (3.10)** |
| G14(N-7)-G19(H-21) | 1.9057 | 2.8978 |
| G14(O-6)-G19(H-1) | **2.2371 (2.10)** | **3.1330 (3.10)** |
| G19(N-7)-G3(H-21) | 1.9895 | 2.9665 |
| G19(O-6)-G3(H-1) | **2.2543 (2.10)** | **3.1384 (3.10)** |
| **G4:G20:G15:G9 tetrad** | N7/O6-H21/H1(Å) | N7/O6-N2/N1(Å) |
| G4(N-7)-G20(H-21) | 1.9805 | 2.9580 |
| G4(O-6)-G20(H-1) | **2.2869 (2.10)** | **3.1551 (3.10)** |
| G20(N-7)-G15(H-21) | 1.9877 | 2.9677 |
| G20(O-6)-G15(H-1) | **2.2261 (2.10)** | **3.1194 (3.10)** |
| G15(N-7)-G9(H-21) | 1.9559 | 2.9311 |
| G15(O-6)-G9(H-1) | **2.2475 (2.10)** | **3.1352 (3.10)** |
| G9(N-7)-G4(H-21) | 2.0572 | 3.0288 |
| G9(O-6)-G4(H-1) | **2.2325 (2.10)** | **3.1274 (3.10)** |


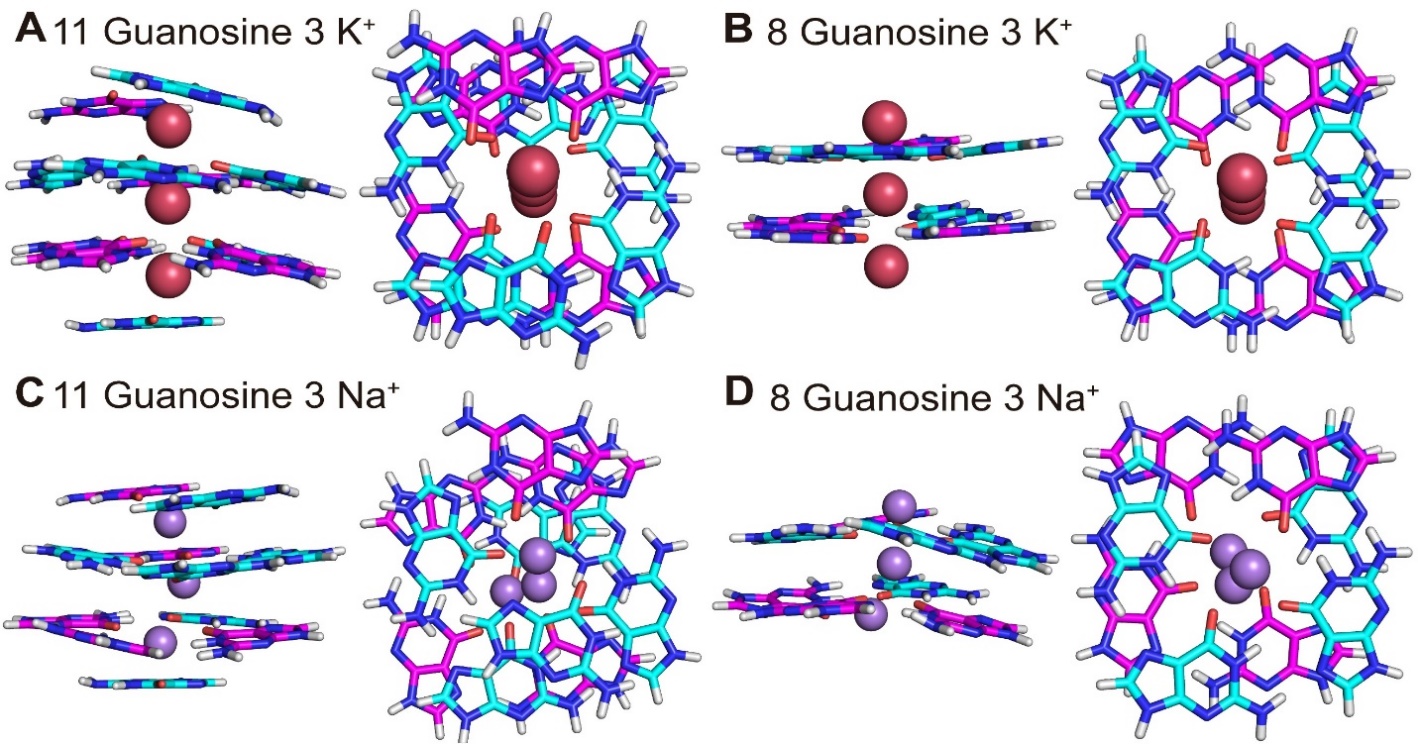


**Figure S16**. K⁺ stabilizes G-quadruplex π-stacking and central ion channels more effectively than Na⁺ in DFT-optimized 2MFU ground states. Each panel displays the optimized geometry (main and top views) of 2MFU-derived quadruplexes with 8 or 11 guanosines and 3 central cations. The sequential π-stacked guanines are indicated from bottom to top in parentheses: (a) 11 guanosines with 3 K⁺ (G3–G4; G10–G8; G13–G15; G19–G21); (b) 8 guanosines with 3 K⁺ (G3–G4; G10–G9; G14–G15; G19–G20); (c) 11 guanosines with 3 Na⁺ (G3–G4; G10–G8; G13–G15; G19–G21); (d) 8 guanosines with 3 Na⁺ (G3–G4; G10–G9; G14–G15; G19–G20). K⁺-containing systems show superior ion-channel alignment and π-stacking order relative to their Na⁺ counterparts, especially in the 11-guanine constructs.

**
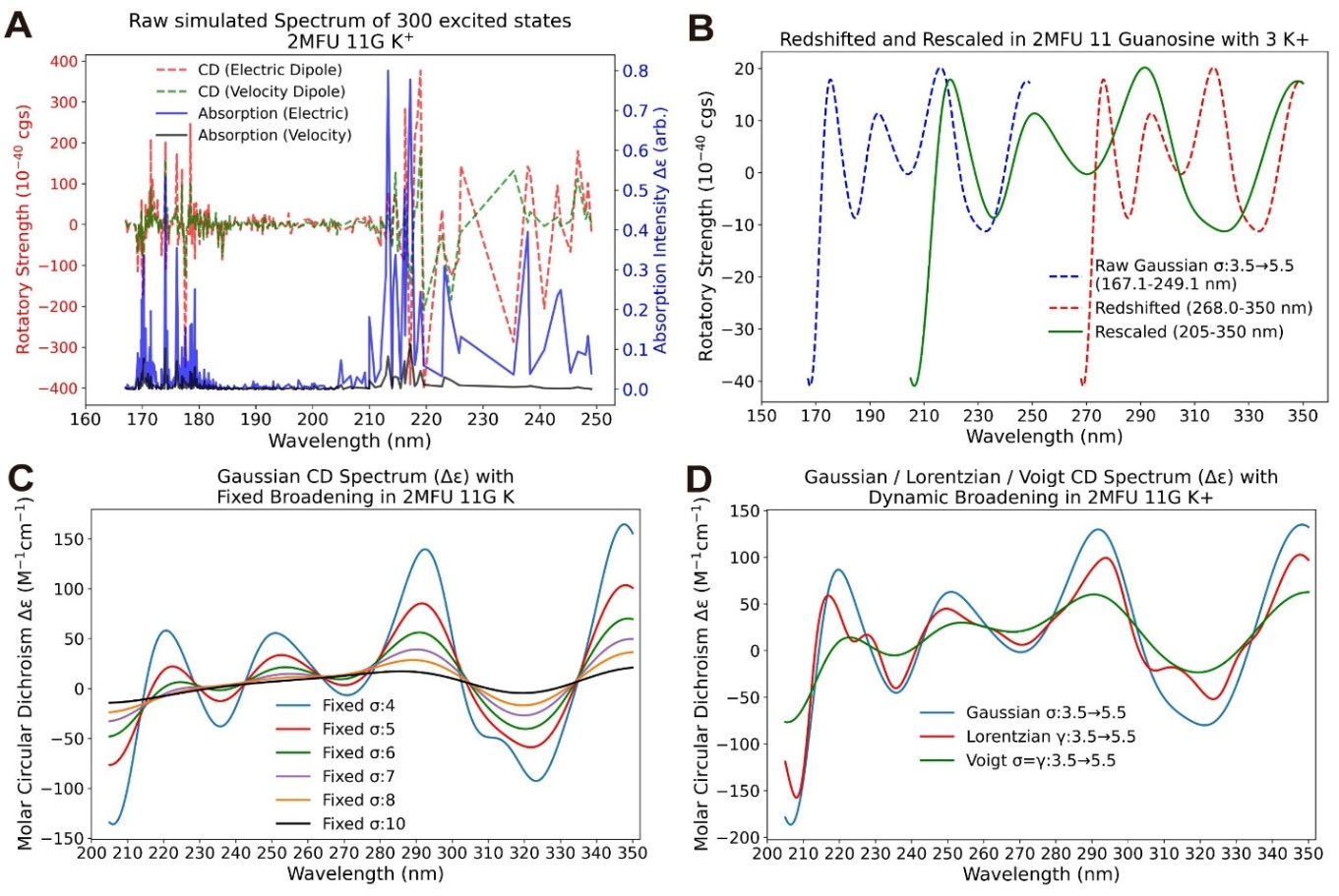
**

**Figure S17**. Optimization of CD spectral broadening highlights Gaussian dynamic broadening as optimal for 2MFU 11G K⁺ system. (a) Raw TD-DFT simulated circular dichroism (CD) and absorption spectra of 2MFU 11G with 3 K⁺ ions (167.1–249.1 nm) computed from 300 excited states exhibit sharp features. (b) Redshifted (268.0–350.0 nm) and rescaled (205–350 nm) spectra from Gaussian convolution (σ = 3.5–5.5 nm), prior to unit conversion. (c CD spectra computed using fixed Gaussian broadening with various widths (σ = 4–10 nm) reveal that excessive smoothing flattens spectral features. (d Final spectra using dynamic broadening (σ = 3.5–5.5) show that Gaussian convolution provides superior agreement with expected peak position and amplitude compared to Lorentzian or Voigt profiles.

**
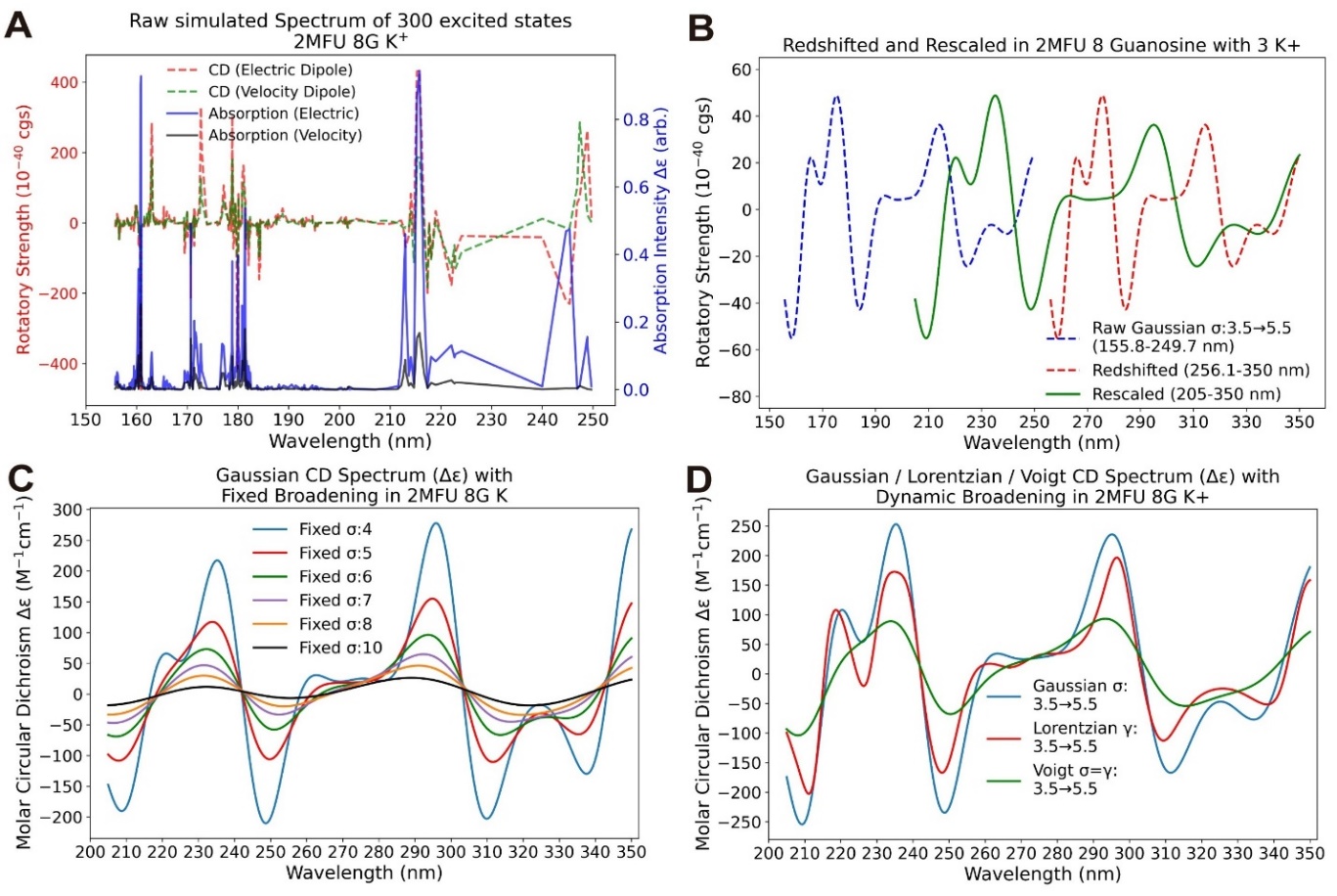
**

**Figure S18**. Optimization of CD spectral broadening highlights Gaussian dynamic broadening as optimal for 2MFU 8G K⁺ system. (a) Raw TD-DFT simulated circular dichroism (CD) and absorption spectra of 2MFU 8G with 3 K⁺ ions (155.8.1–249.7 nm) computed from 300 excited states exhibit sharp features. (b) Redshifted (256.1–350.0 nm) and rescaled (205–350 nm) spectra from Gaussian convolution (σ = 3.5–5.5 nm), prior to unit conversion. (c) CD spectra computed using fixed Gaussian broadening with various widths (σ = 4–10 nm) reveal that excessive smoothing flattens spectral features. (d) Final spectra using dynamic broadening (σ = 3.5–5.5) show that Gaussian model preserves peak and amplitude in signals well.

**Table S9**. Wavelengths of selected excited states (in nm) computed from TD-DFT for 2 DFT 2MFU systems with potassium. The table lists excitation states for specific electronic transitions across 2 optimized geometries: 2MFU 11 Guanosines with 3 K^+^, 2MFU 8 Guanosines with 3 K^+^. Data reflects the spectral range and sampling depth used in the simulated CD and absorption spectra.

| Excitation | Wavelength(nm) in each excitation of structures | |
| --- | --- | --- |
|  | 11 Guanosines with 3 K^+^ | 8 Guanosines with 3 K+ |
| 1 | 249.1 | 249.7 |
| 10 | 237.9 | 222.9 |
| 25 | 215.0 | 212.6 |
| 50 | 203.2 | 197.2 |
| 100 | 191.4 | 184.4 |
| 150 | 183.4 | 177.2 |
| 200 | 179.3 | 165.4 |
| 250 | 173.1 | 160.4 |
| 300 | 167.1 | 155.8 |

## **2. Triplex model preparation and TD‑DFT calculations**

To assign the structural identity of the stable intermediate S₂ observed in the thermal unfolding of 2MFU, we constructed a minimal model of a G‑triplex based on the solution NMR structure of an intramolecular G‑triplex (PDB 2MKM). The model comprised six guanines forming two stacked G‑triads. Pre‑DFT empirical refinement in explicit solvent (100 mM NaCl) was performed with distance restraints derived from the NMR ensemble (**Table S10**) to ensure a starting geometry consistent with experimental coordinates. Hydrogen‑bond distances within the two triads were subsequently constrained during DFT geometry optimisation to preserve Hoogsteen pairing (**Table S11**). TD‑DFT calculations (CAM‑B3LYP/def2‑TZVP, CPCM water) were carried out for 250 excited states; the raw computed rotatory strengths and oscillator strengths are shown in **Figure S19** and the corresponding excitation wavelengths in **Table S12**. To enable direct comparison with experimental SRCD spectra, the discrete excitations were converted to continuous spectra using dynamic Gaussian broadening, followed by affine wavelength alignment and intensity scaling (**Figures S20, S21**). The resulting simulated CD spectrum shows close qualitative agreement with the experimentally derived intermediate-associated spectral signature, supporting assignment of the intermediate to a triplex-like ensemble.

**Table S10.** Pre-DFT optimization on G-triplex with empirical distance restraints in Amber MD simulation derived from the architecture of 2MKM. The π-stacking distances between the C4-C5 bonds of two nucleotides are independently described as the distance between inter-guanosine C4-C4 and C5-C5 in the distance restraint, featured with Syn-Anti as the GBA step. The Hoogsteen base pairs of coplanar inter-residues are described as the distances between the separated N7-H21, N7-N2, O6-H1, and O6-N1. The lower and outer boundaries are allowed at a distance of 0.15 Å. Notably, the hydrogen bond between G10N7 and G6H21 is not constrained to faithfully reflect the NMR structure in publication for the 2MKM deposition.^[42]^

| **Residues of 2MKM** | **Distance Restraints** | | **Pre-DFT Refined Structures** | |
| --- | --- | --- | --- | --- |
| **Pi-stacking Between Guanosines** | C4-C4(Å) | C5-C5(Å) | C4-C4(Å) | C5-C5(Å) |
| G1(C)-G2(C), **Syn-Anti** | 4.36 ± 0.15 | 3.53 ± 0.15 | 4.3278 | 3.4350 |
| G5(C)-G6(C), **Syn-Anti** | 4.36 ± 0.15 | 3.53 ± 0.15 | 4.2390 | 3.5249 |
| G10(C)-G11(C), **Syn-Anti** | 4.36 ± 0.15 | 3.53 ± 0.15 | 4.4355 | 3.5771 |
| **Hydrogen bond within G-tetrads** | N7/O6-H21/H1(Å) | N7/O6-N2/N1(Å) | N7/O6-H21/H1(Å) | N7/O6-N2/N1(Å) |
| **G10:G6:G1 triad** |  |  |  |  |
| G10(N-7)-G6(H-21) | x | x | 1.9430 | 2.9421 |
| G10(O-6)-G6(H-1) | 1.95 ± 0.15 | 2.95 ± 0.15 | 2.0904 | 2.9928 |
| G6(N-7)-G1(H-21) | 1.99 ± 0.15 | 2.9 ± 0.15 | 1.8690 | 2.8713 |
| G6(O-6)-G1(H-1) | 1.95 ± 0.15 | 2.95 ± 0.15 | 2.1420 | 3.0683 |
| **G2:G5:G11 triad** |  |  |  |  |
| G2(N-7)-G5(H-21) | 1.99 ± 0.15 | 2.9 ± 0.15 | 1.9498 | 2.9178 |
| G2(O-6)-G5(H-1) | 1.95 ± 0.15 | 2.95 ± 0.15 | 2.2681 | 3.1480 |
| G5(N-7)-G11(H-21) | 1.99 ± 0.15 | 2.9 ± 0.15 | 1.9725 | 2.9678 |
| G5(O-6)-G11(H-1) | 1.95 ± 0.15 | 2.95 ± 0.15 | 2.2737 | 3.2014 |

**Table S11.** Constrained hydrogen-bond distances used in DFT geometry optimization of triplex and hybrid triplex structures. Distance constraints were applied to coplanar guanine residues in triplex (6 bases) using the ORCA quantum chemistry package. Hoogsteen hydrogen bonds were defined via fixed inter-residue distances (N7–H21, N7–N2, O6–H1, O6–N1), consistent with values from post-equilibrated MD structures. Target distances were adopted from X-ray data in study^[41]^: N7–H21, 1.99 ± 0.15 Å; N7–N2, 2.90 ± 0.15 Å; O6–H1, 1.95 ± 0.15 Å; O6–N1, 2.95 ± 0.15 Å. Distances exceeding boundaries were re-constrained to the nearest limit (values in **bold** and *parentheses*). Planarity and π-stacking between guanines were unconstrained to allow geometric relaxation during optimization.

| **Pre-DFT structure (2mkm)** | **Triplex Stem** | |
| --- | --- | --- |
| **Hydrogen bond within triplex** | 6 Guanosine with planarity | |
| **G10:G6:G1 triad** | N7/O6-H21/H1(Å) | N7/O6-N2/N1(Å) |
| G10(N-7)-G6(H-21) | 1.943 | 2.9421 |
| G10(O-6)-G6(H-1) | 2.0904 | 2.9928 |
| G6(N-7)-G1(H-21) | 1.869 | 2.8713 |
| G6(O-6)-G1(H-1) | **2.142 (2.10)** | 3.0683 |
| **G2:G5:G11 triad** | N7/O6-H21/H1(Å) | N7/O6-N2/N1(Å) |
| G2(N-7)-G5(H-21) | 1.9498 | 2.9178 |
| G2(O-6)-G5(H-1) | **2.2681(2.10)** | **3.148 (3.10)** |
| G5(N-7)-G11(H-21) | 1.9725 | 2.9678 |
| G5(O-6)-G11(H-1) | **2.2737(2.10)** | **3.2014 (3.10)** |

**
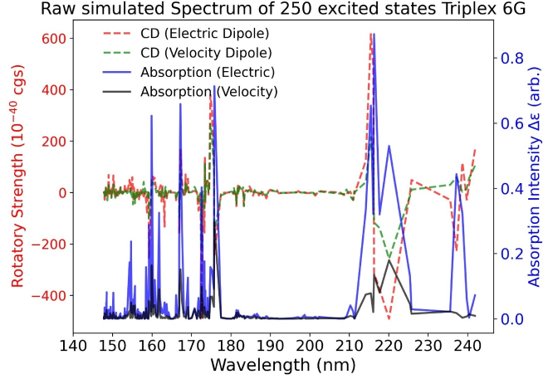
**

**Figure S19.** Raw simulated circular dichroism (CD) and absorption spectrum from TD-DFT excited states for G-triplex**.** Spectra were computed for 140–250 excited states before applying broadening, red-shift, or intensity rescaling. Shown are CD spectra (electric and velocity dipole forms) and electronic absorption spectra (electric and velocity formulations) for DFT-optimized conformation for G-triplex with 6 guanosines. X-axis: wavelength (nm). Left Y-axis: rotatory strength (×10⁻⁴⁰ cgs). Right Y-axis: absorption intensity (arb. units).

**Table S12.** Wavelengths of selected excited states (in nm) computed from TD-DFT for G-triplex structure. The table lists excitation states for specific electronic transitions across the optimized geometry for G-triplex (6G, 250 states). Data reflects the spectral range and sampling depth used in the simulated CD and absorption spectra.

| Excitation |  |
| --- | --- |
|  | Triplex 6G |
| 1 | 241.9 |
| 10 | 217.7 |
| 25 | 201.4 |
| 50 | 185.6 |
| 100 | 170.8 |
| 150 | 158.8 |
| 200 | 151.5 |
| 250 | 147.8 |

**
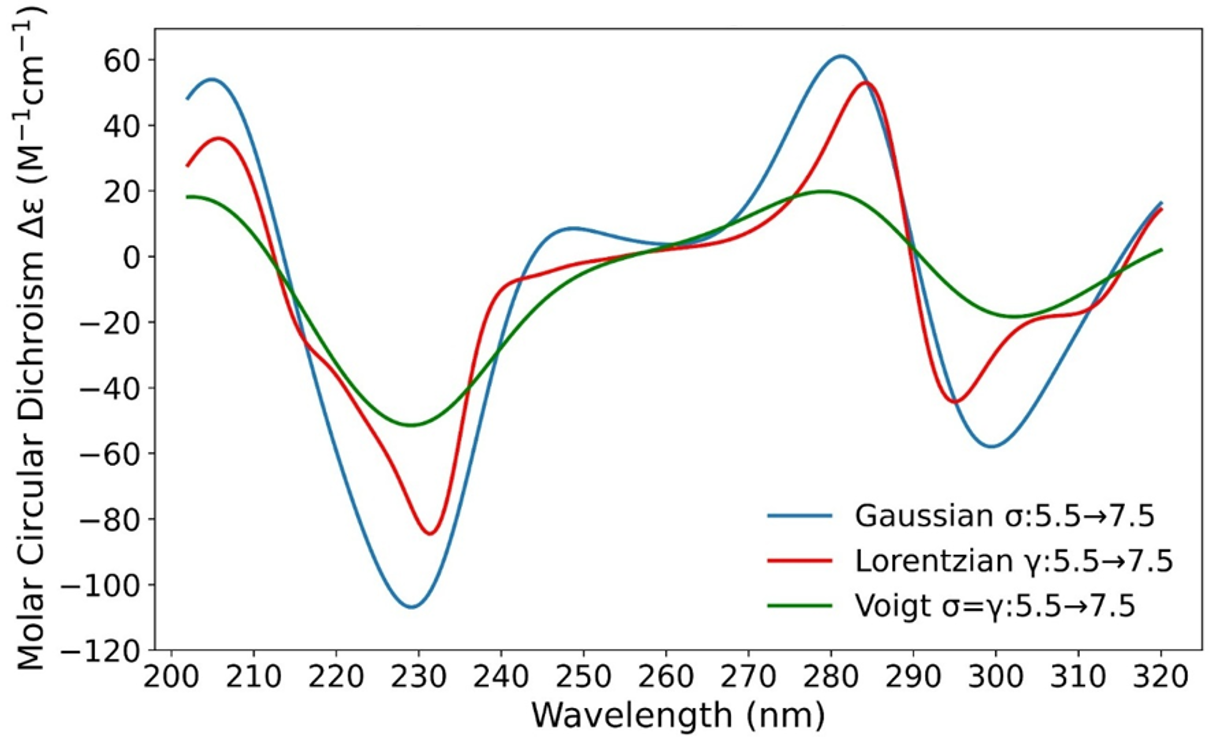
**

**Figure S20**. Final simulated circular dichroism (CD) spectra of representative intermediate structures using optimized dynamic broadening widths for G-triplex. Simulated CD spectra were by convolving TD-DFT molar circular dichroism with wavelength-dependent (dynamic) Gaussian or Lorentzian or Voigt functions, to assess the spectral resolution and optimal broadening. Shown are broadened CD spectra with parameters of dynamic width (σ, γ) from 5.5 to 7.5 nm for Triplex with 6 guanosines. X-axis: wavelength (nm); Y-axis: molar circular dichroism *Δε* (M^-1^·cm^-1^ or L·mol^-1^·cm^-1^).

**
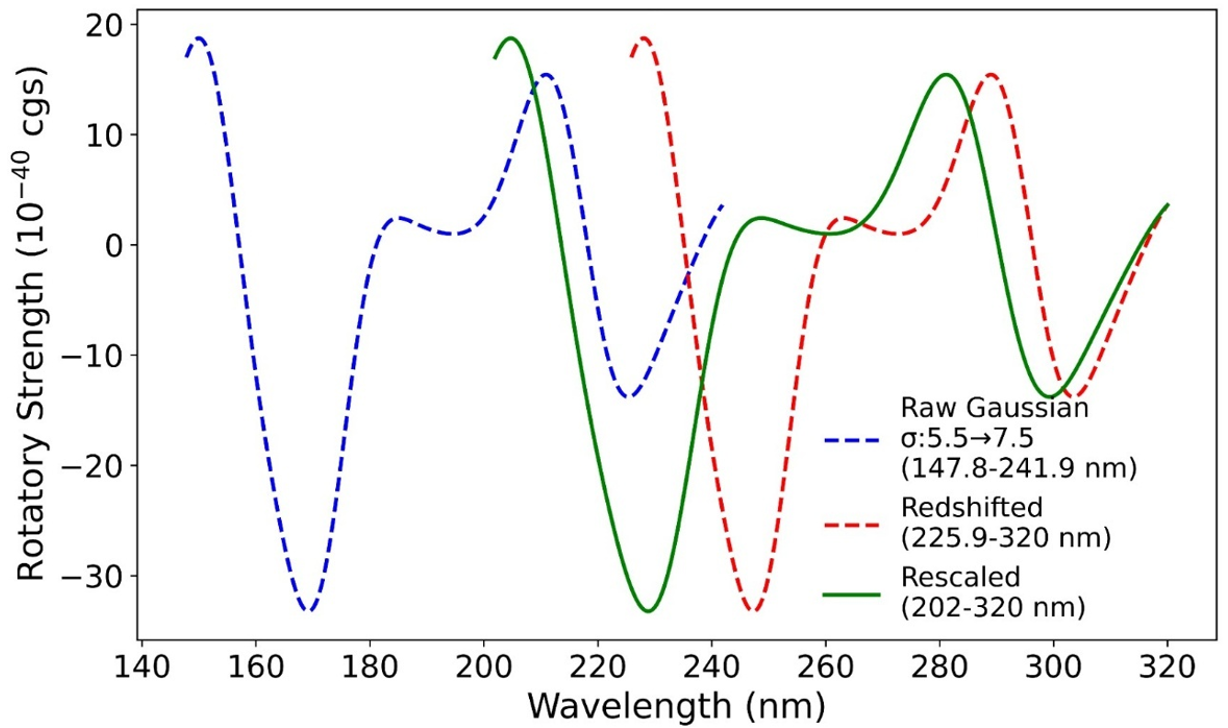
**

**Figure S21.** Broadening, redshifted and rescaled circular dichroism (CD) spectra of representative intermediate structures before conversion of units of G-triplex. To enable comparison with experimental spectrum, simulated CD signals were post-processed sequentially by (1) Guassian broadening with dynamic width σ from 5.5 to 7.5 nm (blue dashed), (2) redshifted till the maximum to 320 nm (red dashed), (3) rescaled till the minimum to 202 nm (green solid). Shown are redshifted (by 78.1 nm) and rescaled (scaling factor 1.254) CD spectra, before conversion of units. X-axis: wavelength (nm); Y-axis: rotatory strength (×10⁻⁴⁰ cgs).

# IX. Integrative summary

Key stacking poses across guanine steps for 2MFU, integrating NMR ensemble, MetaD basins, and DFT models, are summarized in **Table S13**

**Table S13**. Summary of stacking poses across Guanosine step among NMR-derived, metadynamic basin, and the DFT ground state. Stacking poses are evaluated based on glycosidic bond angle (Syn/Anti), and base stacking modes. G8-G9 *Syn-Syn* base step in DFT ground state is poorly stacked.

|  | **NMR structure** | **Basin Native** | **DFT ground state** |
| --- | --- | --- | --- |
| **G2-G3** | x | Syn-Syn | x |
| **Outside** | x | Partial 5/6-ring | x |
| **G3-G4** | Syn-Anti | Syn-Anti | Syn-Anti |
| **Stem** | 5-ring | 5-ring | 5-ring |
| **G8-G9** | Syn-Syn | Syn-Syn | Syn-Syn |
| **Outside** | Partial 5/6-ring | Partial 5/6-ring | **Poor*** |
| **G9-G10** | Syn-Anti | Syn-Anti | Syn-Anti |
| **Stem** | 5-ring | 5-ring | 5-ring |
| **G13-G10** | x | x | x |
| **snap-back** | x | x | x |
| **G13-G14** | Anti-Syn | Anti-Syn | Anti-Syn |
| **Outside** | Partial 6-ring | Partial 6-ring | Partial 6-ring |
| **G14-G15** | Syn-Anti | Syn-Anti | Syn-Anti |
| **Stem** | 5-ring | 5-ring | 5-ring |
| **G19-G20** | Syn-Anti | Syn-Anti | Syn-Anti |
| Stem | 5-ring | 5-ring | 5-ring |
| **G20-G21** | Anti-Anti | Anti-Anti | Anti-Anti |
| **Outside** | Partial 5/6-ring | Partial 5/6-ring | Partial 5/6-ring |
| **G19-G21** | x | x | x |
| **snap-back** | x | x | x |

# References

[1] S. A. Dvorkin, A. I. Karsisiotis, M. Webba da Silva, *Encoding canonical DNA quadruplex structure,* *Science advances* **2018**, *4*, eaat3007, <https://doi.org/10.1126/sciadv.aat3007>.

[2] R. Hussain, T. Javorfi, G. Siligardi, *8.23 Spectroscopic analysis: synchrotron radiation circular dichroism,* *Comprehensive Chirality* **2012**, *8*, 438-448, <https://doi.org/10.1016/B978-0-08-095167-6.00841-7>.

[3] R. Hussain, T. Jávorfi, G. Siligardi, *Circular dichroism beamline B23 at the Diamond Light Source,* *J. Synchrotron Radiat.* **2012**, *19*, 132-135, <https://doi.org/10.1107/s0909049511038982>.

[4] H. Abdi, L. J. Williams, *Principal component analysis,* *WIREs Computational Statistics* **2010**, *2*, 433-459, <https://doi.org/10.1002/wics.101>.

[5] L. v. d. M. a. G. Hinton, *Visualizing Data using t-SNE,* *J. Mach. Learn. Res.* **2008**, *9*, 2579--2605, <http://jmlr.org/papers/v9/vandermaaten08a.html>

[6] P. C. Hansen, *The truncatedSVD as a method for regularization,* *BIT Numer. Math.* **1987**, *27*, 534-553, <https://doi.org/10.1007/BF01937276>.

[7] W. D. Cornell, P. Cieplak, C. I. Bayly, I. R. Gould, K. M. Merz, D. M. Ferguson, D. C. Spellmeyer, T. Fox, J. W. Caldwell, P. A. Kollman, *A Second Generation Force Field for the Simulation of Proteins, Nucleic Acids, and Organic Molecules,* *Journal of the American Chemical Society* **1995**, *117*, 5179-5197, <https://doi.org/10.1021/ja00124a002>.

[8] M. Krepl, M. Zgarbová, P. Stadlbauer, M. Otyepka, P. Banáš, J. Koča, T. E. Cheatham, III, P. Jurečka, J. Šponer, *Reference Simulations of Noncanonical Nucleic Acids with Different χ Variants of the AMBER Force Field: Quadruplex DNA, Quadruplex RNA, and Z-DNA,* *Journal of Chemical Theory and Computation* **2012**, *8*, 2506-2520, <https://doi.org/10.1021/ct300275s>.

[9] M. Zgarbová, F. J. Luque, J. Sponer, T. E. Cheatham, 3rd, M. Otyepka, P. Jurečka, *Toward Improved Description of DNA Backbone: Revisiting Epsilon and Zeta Torsion Force Field Parameters,* *J Chem Theory Comput* **2013**, *9*, 2339-2354, <https://doi.org/10.1021/ct400154j>.

[10] M. Zgarbová, J. Šponer, M. Otyepka, T. E. Cheatham, 3rd, R. Galindo-Murillo, P. Jurečka, *Refinement of the Sugar-Phosphate Backbone Torsion Beta for AMBER Force Fields Improves the Description of Z- and B-DNA,* *J Chem Theory Comput* **2015**, *11*, 5723-5736, <https://doi.org/10.1021/acs.jctc.5b00716>.

[11] M. Zgarbová, J. Šponer, P. Jurečka, *Z-DNA as a Touchstone for Additive Empirical Force Fields and a Refinement of the Alpha/Gamma DNA Torsions for AMBER,* *J. Chem. Theory Comput.* **2021**, *17*, 6292-6301, <https://doi.org/10.1021/acs.jctc.1c00697>.

[12] I. S. Joung, T. E. Cheatham, 3rd, *Determination of alkali and halide monovalent ion parameters for use in explicitly solvated biomolecular simulations,* *J. Phys. Chem. B* **2008**, *112*, 9020-9041, <https://doi.org/10.1021/jp8001614>.

[13] D. A. Case, H. M. Aktulga, K. Belfon, D. S. Cerutti, G. A. Cisneros, V. W. D. Cruzeiro, N. Forouzesh, T. J. Giese, A. W. Götz, H. Gohlke, S. Izadi, K. Kasavajhala, M. C. Kaymak, E. King, T. Kurtzman, T. S. Lee, P. Li, J. Liu, T. Luchko, R. Luo, M. Manathunga, M. R. Machado, H. M. Nguyen, K. A. O'Hearn, A. V. Onufriev, F. Pan, S. Pantano, R. Qi, A. Rahnamoun, A. Risheh, S. Schott-Verdugo, A. Shajan, J. Swails, J. Wang, H. Wei, X. Wu, Y. Wu, S. Zhang, S. Zhao, Q. Zhu, T. E. Cheatham, 3rd, D. R. Roe, A. Roitberg, C. Simmerling, D. M. York, M. C. Nagan, K. M. Merz, Jr., *AmberTools,* *J. Chem. Inf. Model* **2023**, *63*, 6183-6191, <https://doi.org/10.1021/acs.jcim.3c01153>.

[14] M. J. Abraham, T. Murtola, R. Schulz, S. Páll, J. C. Smith, B. Hess, E. Lindahl, *GROMACS: High performance molecular simulations through multi-level parallelism from laptops to supercomputers,* *SoftwareX* **2015**, *1-2*, 19-25, <https://doi.org/10.1016/j.softx.2015.06.001>.

[15] M. R. Shirts, C. Klein, J. M. Swails, J. Yin, M. K. Gilson, D. L. Mobley, D. A. Case, E. D. Zhong, *Lessons learned from comparing molecular dynamics engines on the SAMPL5 dataset,* *J. Comput. Aided. Mol. Des.* **2017**, *31*, 147-161, <https://doi.org/10.1007/s10822-016-9977-1>.

[16] G. Bussi, D. Donadio, M. Parrinello, *Canonical sampling through velocity rescaling,* *J. Chem. Phys.* **2007**, *126*, 014101, <https://doi.org/10.1063/1.2408420>.

[17] H. J. C. Berendsen, J. P. M. Postma, W. F. van Gunsteren, A. DiNola, J. R. Haak, *Molecular dynamics with coupling to an external bath,* *J. Chem. Phys.* **1984**, *81*, 3684-3690, <https://doi.org/10.1063/1.448118>.

[18] M. Parrinello, A. Rahman, *Polymorphic transitions in single crystals: A new molecular dynamics method,* *J. Appl. Phys.* **1981**, *52*, 7182-7190, <https://doi.org/10.1063/1.328693>.

[19] B. A. Wells, A. L. Chaffee, *Ewald Summation for Molecular Simulations,* *J. Chem. Theory Comput.* **2015**, *11*, 3684-3695, <https://doi.org/10.1021/acs.jctc.5b00093>.

[20] T. Darden, D. York, L. Pedersen, *Particle mesh Ewald: An N⋅log(N) method for Ewald sums in large systems,* *J. Chem. Phys.* **1993**, *98*, 10089-10092, <https://doi.org/10.1063/1.464397>.

[21] S. Bottaro, F. Di Palma, G. Bussi, *The role of nucleobase interactions in RNA structure and dynamics,* *Nucleic Acids Res.* **2014**, *42*, 13306-13314, <https://doi.org/10.1093/nar/gku972>.

[22] F. Eisenhaber, P. Lijnzaad, P. Argos, C. Sander, M. Scharf, *The double cubic lattice method: Efficient approaches to numerical integration of surface area and volume and to dot surface contouring of molecular assemblies,* *J. Comput. Chem.* **1995**, *16*, 273-284, <https://doi.org/10.1002/jcc.540160303>.

[23] M. Webba da Silva, M. Trajkovski, Y. Sannohe, N. Ma'ani Hessari, H. Sugiyama, J. Plavec, *Design of a G-quadruplex topology through glycosidic bond angles,* *Angew Chem Int Ed Engl* **2009**, *48*, 9167-9170, <https://doi.org/10.1002/anie.200902454>.

[24] J. D. Hunter, *Matplotlib: A 2D Graphics Environment,* *Comput. Sci. Eng.* **2007**, *9*, 90-95, <https://doi.org/10.1109/MCSE.2007.55>.

[25] S. Bottaro, G. Bussi, G. Pinamonti, S. Reißer, W. Boomsma, K. Lindorff-Larsen, *Barnaba: software for analysis of nucleic acid structures and trajectories,* *RNA* **2019**, *25*, 219-231, <https://doi.org/10.1261/rna.067678.118>.

[26] R. T. McGibbon, K. A. Beauchamp, M. P. Harrigan, C. Klein, J. M. Swails, C. X. Hernández, C. R. Schwantes, L. P. Wang, T. J. Lane, V. S. Pande, *MDTraj: A Modern Open Library for the Analysis of Molecular Dynamics Trajectories,* *Biophys. J.* **2015**, *109*, 1528-1532, <https://doi.org/10.1016/j.bpj.2015.08.015>.

[27] M. L. Waskom, *seaborn: statistical data visualization,* *J. Open Source Softw.* **2021**, *6*, 3021, <https://doi.org/10.21105/joss.03021>

[28] S. Piana, A. Laio, *A bias-exchange approach to protein folding,* *J. Phys. Chem. B* **2007**, *111*, 4553-4559, <https://doi.org/10.1021/jp067873l>.

[29] F. Neese, *The ORCA program system,* *WIREs Comput. Mol. Sci.* **2012**, *2*, 73-78, <https://doi.org/10.1002/wcms.81>.

[30] F. Neese, *Software update: The ORCA program system—Version 5.0,* *WIREs Comput. Mol. Sci.* **2022**, *12*, e1606, <https://doi.org/10.1002/wcms.1606>.

[31] T. Yanai, D. P. Tew, N. C. Handy, *A new hybrid exchange–correlation functional using the Coulomb-attenuating method (CAM-B3LYP),* *Chem. Phys. Lett.* **2004**, *393*, 51-57, <https://www.sciencedirect.com/science/article/pii/S0009261404008620>

[32] F. Weigend, R. Ahlrichs, *Balanced basis sets of split valence, triple zeta valence and quadruple zeta valence quality for H to Rn: Design and assessment of accuracy,* *Phys. Chem. Chem. Phys.* **2005**, *7*, 3297-3305, <https://doi.org/10.1039/b508541a>.

[33] V. Barone, M. Cossi, *Quantum Calculation of Molecular Energies and Energy Gradients in Solution by a Conductor Solvent Model,* *J. Phys. Chem. A* **1998**, *102*, 1995-2001, <https://doi.org/10.1021/jp9716997>.

[34] F. Neese, F. Wennmohs, A. Hansen, U. Becker, *Efficient, approximate and parallel Hartree–Fock and hybrid DFT calculations. A ‘chain-of-spheres’ algorithm for the Hartree–Fock exchange,* *Chem. Phys.* **2009**, *356*, 98-109, <https://doi.org/10.1016/j.chemphys.2008.10.036>.

[35] S. Hirata, M. Head-Gordon, *Time-dependent density functional theory within the Tamm–Dancoff approximation,* *Chem. Phys. Lett.* **1999**, *314*, 291-299, <https://doi.org/10.1016/S0009-2614(99)01149-5>.

[36] E. Runge, E. K. U. Gross, *Density-Functional Theory for Time-Dependent Systems,* *Phys. Rev. Lett.* **1984**, *52*, 997-1000, <https://doi.org/10.1103/PhysRevLett.52.997>.

[37] P. J. Stephens, F. J. Devlin, C. F. Chabalowski, M. J. Frisch, *Ab Initio Calculation of Vibrational Absorption and Circular Dichroism Spectra Using Density Functional Force Fields,* *J. Phys. Chem.* **1994**, *98*, 11623-11627, <https://doi.org/10.1021/j100096a001>.

[38] A. Jiemchooroj, P. Norman, *Electronic circular dichroism spectra from the complex polarization propagator,* *J. Chem. Phys.* **2007**, *126*, <https://doi.org/10.1063/1.2716660>.

[39] P. L. Polavarapu, *Kramers−Kronig Transformation for Optical Rotatory Dispersion Studies,* *J. Phys. Chem. A* **2005**, *109*, 7013-7023, <https://doi.org/10.1021/jp0524328>.

[40] C. R. Harris, K. J. Millman, S. J. van der Walt, R. Gommers, P. Virtanen, D. Cournapeau, E. Wieser, J. Taylor, S. Berg, N. J. Smith, R. Kern, M. Picus, S. Hoyer, M. H. van Kerkwijk, M. Brett, A. Haldane, J. F. del Río, M. Wiebe, P. Peterson, P. Gérard-Marchant, K. Sheppard, T. Reddy, W. Weckesser, H. Abbasi, C. Gohlke, T. E. Oliphant, *Array programming with NumPy,* *Nature* **2020**, *585*, 357-362, <https://doi.org/10.1038/s41586-020-2649-2>.

[41] G. Laughlan, A. I. Murchie, D. G. Norman, M. H. Moore, P. C. Moody, D. M. Lilley, B. Luisi, *The high-resolution crystal structure of a parallel-stranded guanine tetraplex,* *Science* **1994**, *265*, 520-524, <https://doi.org/10.1126/science.8036494>.

[42] L. Cerofolini, J. Amato, A. Giachetti, V. Limongelli, E. Novellino, M. Parrinello, M. Fragai, A. Randazzo, C. Luchinat, *G-triplex structure and formation propensity,* *Nucleic Acids Research* **2014**, *42*, 13393-13404, <https://doi.org/10.1093/nar/gku1084>.
